# Palladium-Catalyzed C-H Functionalization and Flame-Retardant Properties of Isophosphinolines

**DOI:** 10.3390/molecules29215104

**Published:** 2024-10-29

**Authors:** Karen-Pacelye Mengue Me Ndong, Mina Hariri, Gabin Mwande-Maguene, Jacques Lebibi, Fatemeh Darvish, Christine Safi, Kouceila Abdelli, Adam Daïch, Claire Negrell, Rodolphe Sonnier, Loic Dumazert, Abdou Rachid Issaka Ibrahim, Ilagouma Amadou Tidjani, David Virieux, Tahar Ayad, Jean-Luc Pirat

**Affiliations:** 1 ICGM, Univ Montpellier, ENSCM, CNRS, 34090 Montpellier, France; guemenkaren@gmail.com (K.-P.M.M.N.); mina.hariri67@yahoo.com (M.H.); claire.negrell@enscm.fr (C.N.); david.virieux@enscm.fr (D.V.); 2 Université des Sciences et Techniques de Masuku, Franceville BP 942, Gabon; gabin.maguene@gmail.com (G.M.-M.); jlebibi@hotmail.com (J.L.); 3 Department of Chemistry, K. N. Toosi University of Technology, Tehran 19991-43344, Iran; darvish@kntu.ac.ir; 4 Université Le Havre Normandie, Normandie Univ, URCOM UR 3221, FR CNRS 3038, 76600 Le Havre, France; christine.safi@univ-lehavre.fr (C.S.); kouceila.abdelli@etu.univ-lehavre.fr (K.A.); adam.daich@univ-lehavre.fr (A.D.); 5 Polymers Composites and Hybrids (PCH), IMT Mines Ales, 30319 Ales, France; rodolphe.sonnier@mines-ales.fr (R.S.); loic.dumazert@mines-ales.fr (L.D.); 6 University Abdou Moumouni of Niamey, Niamey BP 10896, Niger; abdrachid2i@gmail.com (A.R.I.I.); ilagoumat@gmail.com (I.A.T.)

**Keywords:** C-H functionalization, isophosphinoline oxide, flame retardant, regioselective palladium-catalyzed

## Abstract

C-H activation is a powerful strategy for forming C-C bonds without the need for prefunctionalization. In this paper, we present a general, direct, and regioselective palladium-catalyzed functionalization of a phosphorus heterocycle, 2-phenyl-1*H*-isophosphinoline 2-oxide. The mild reaction conditions enabled the introduction of various functionalized alkenes. Moreover, the flame-retardant properties of selected products clearly highlighted the synergy between the phosphine oxide and another heteroatom-based group, even in the condensed phase.

## 1. Introduction

Cross-coupling reactions, discovered over 50 years ago, have evolved into a mature technology with significant impact and impressive advancements both in academia and industry [1]. Current research focuses on enhancing the efficiency of these reactions and broadening their scope, with the understanding that even minor improvements in catalyst efficiency can lead to substantial economic benefits [2,3,4]. Typically, C–C bond formation via cross-coupling reactions involves a transition metal-catalyzed reaction between a halogenated derivative and an organometallic species. However, the need for multiple synthetic steps to prepare the halogenated derivative and/or organometallic reagents represents one of the few limitations of these otherwise robust synthetic tools. In contrast, transition-metal-catalyzed C–H activation has recently emerged as an efficient and streamlined alternative to traditional coupling reactions [5]. By directly functionalizing the C–H bond, these new strategies circumvent the need for cumbersome pre-functionalization steps, thereby offering greater atom economy, reduced waste, and time efficiency [6,7,8]. Notably, recent advances in direct C(sp^2^) C–H functionalization have facilitated the modification of *N*- and *O*-heterocycles [9].

Otherwise, in the field of heteroatom chemistry, organophosphorus compounds constitute a diverse class of molecules that have attracted the attention of synthetic chemists and industry alike. They are versatile molecules with plentiful applications across domains as diverse as organic synthesis [10,11], including agrochemicals [12], pharmaceuticals [13,14], and cosmetics [15]. Moreover, they have found drawn interest in the field of material science as building blocks to elaborate functional polymers [16], metal organic frameworks (MOFs) [17,18], and electroluminescent materials [19]. Even when organophosphorus compounds are not the primary constituent of a material, they contribute significantly. For instance, they can act as photo-initiators [20] or impart key properties, enabling the recovery of strategic metals [21], or functioning as flame retardants [22,23,24,25].

Despite their importance, the design of novel phosphorus-based molecules remains highly desirable. Phosphorus heterocycles, where phosphorus atoms are embedded within the heterocyclic core have received less attention, aside from DOPO, which is one of the most recent commercial successes in the flame-retardant industry. Furthermore, the post-functionalization of such building blocks remains an under-explored area. Within the scope of our ongoing project dedicated to the development of isophosphinoline building blocks, recently reported by our group [26], we describe herein their regioselective direct C-2 functionalization through atom-economic C–H activation reactions. We envision achieving this task through reactions with different Michael acceptors. To the best of our knowledge, there are no reported examples of direct regioselective C-2 functionalization reactions of vinylphosphine oxide derivatives or phosphorus-containing heterocycles, including isophosphinolines. In 2021, Zhang first reported the synthesis of P-stereogenic phosphinates through allylic alkylation of *H*-phosphinates [27]. To illustrate the potential of these new molecules, alongside the development of a novel C–H coupling reaction, we will explore their applications as flame retardants.

## 2. Results and Discussion

### 2.1. C-H Functionalization of Isophosphinolines Catalyzed with Palladium Acetate

**Scheme 1:**
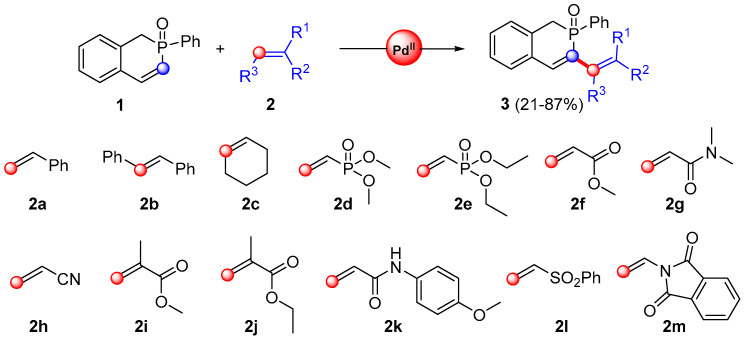
C–H functionalization of 1*H*-isophosphinoline 2-oxide (**1**) catalyzed with Pd(OAc)_2_.

For the preliminary study, we employed 2-phenyl-1*H*-isophosphinoline 2-oxide (**1**) as model substrate. This compound had been previously synthesized by our group [26]. Under the conditions described by one of us [6], we engaged isophosphinoline 2-oxide (**1**) with various terminal, internal, and functionalized alkenes in the presence of palladium acetate (10 mol%) as a catalyst, silver carbonate (1.17 eq.) as an oxidant, and pivalic acid (4 mL) as an additive (Scheme 1). The reactions were heated at 90 °C for 20 h. Based on the results presented in Table 1, it is clear that the effectiveness of the C–H functionalization is strongly influenced by the nature of the vinyl partners.

### 2.2. C-H Functionalization Discussion

**Scheme 2:**
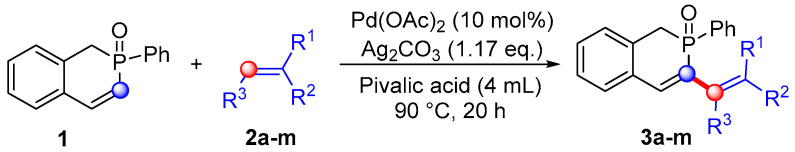
Regioselective C2 functionalization of 2-phenyl-1*H*-isophosphinoline 2-oxide (**1**).

When alkenes such as styrene (Table 1, entry 1, **2a**) or trans-stilbene (Table 1, entry 2, **2b**) were used, the corresponding coupling products **3a** and **3b** were isolated after treatment and purification by column chromatography, with yields of 57% and 28%, respectively. Surprisingly, the reaction of cyclohexene (Table 1, entry 3, **2c**) resulted in the formation of a regioisomer **3c** of the expected product in 38% isolated yield. Such isomerization is not so rare and typically occurs during the reductive β-elimination step [28,29,30]. Alkenes bearing electron-withdrawing groups, such as methyl or ethyl vinylphosphonates, (**2d**) and (**2e**), and methyl acrylate (**2f**), afforded the corresponding adducts in yields ranging from 50% up to 87%. The reactions were totally regioselective, as only coupling at the C2 position of the isophosphinoline 2-oxide (**1**) was observed. For these unsymmetrical alkenes, the reactions also occurred regio- and stereoselectively, forming (*E*)-isomers (Table 1, entries 4–6). Similarly, *N,N*-dimethylacrylamide (**2g**) and acrylonitrile (**2h**) furnished the corresponding products **3g** and **3h** in isolated yields of 25% and 44%, respectively (Table 1, entries 7 and 8). The same regio- and stereoselectivity was observed, as described by Hong group [26]. From these reactions, it becomes evident that the phosphoryl group (P=O) acts as an effective “ortho-directing group”, thereby promoting the reaction exclusively at the C2 position. In contrast, when methyl methacrylate (**2i**) and ethyl methacrylate (**2j**) were used as reagents, a mixture of two regioisomers **3ia-b** and **3ja-b**, along with compounds **3ic** and **3jc** resulting from double activation, were formed (Table 1, entries 9 and 10). Following isolation through column chromatography, the structures of these compounds were assigned using proton NMR analysis. They are consistent with the observations reported by Hong in the studies involving the regioselective palladium-catalyzed olefination of coumarins via aerobic oxidative Heck reactions [31]. Of interest, isophosphinolines **3ib** and **3jb** were alkene-regioisomers of **3ia** and **3ja**, respectively, as previously observed for product **3c**. The double adducts **3ic** and **3jc** resulted, for their part, from a double C–H activation. They were directly formed from **3ib** and **3jb** by reaction with isophosphinoline (**1**). Primary amides, such as *N*-(4-methoxybenzyl)acrylamide (**2k**) or phenylvinylsulfone (**2l**) also afforded the corresponding adducts **3k** and **3l**, respectively, without reaction alteration with NH amide in the case of **3k**. Finally, an electron-rich alkene, 2-vinylisoindoline-1,3-dione (**2m**), was engaged successfully in this reaction, furnishing a single isomer **3m**, albeit in low yield. The structure of **3** was also confirmed by X-ray analysis of one representative example, compound **3l** (see Scheme 2, Figure 3 and ESI).

## 3. Flame-Retardant Potential of Certain Molecules

Despite the moderate yields obtained in certain cases, we decided to investigate the potential flame-retardant properties of some of our new phosphorus-containing heterocycles.

### Flammability of Functional Isophosphinolines Results and Discussion

To assess the flame-retardant effect of one molecule, it is essential to test it within a polymer matrix. However, assessing the thermal decomposition of the molecule alone can provide useful information if it undergoes pyrolysis rather than vaporization, which is effectively the case for isophosphinolines due to their high molecular mass and the poor volatility associated with phosphoryl-based molecules.

**Figure 1:**
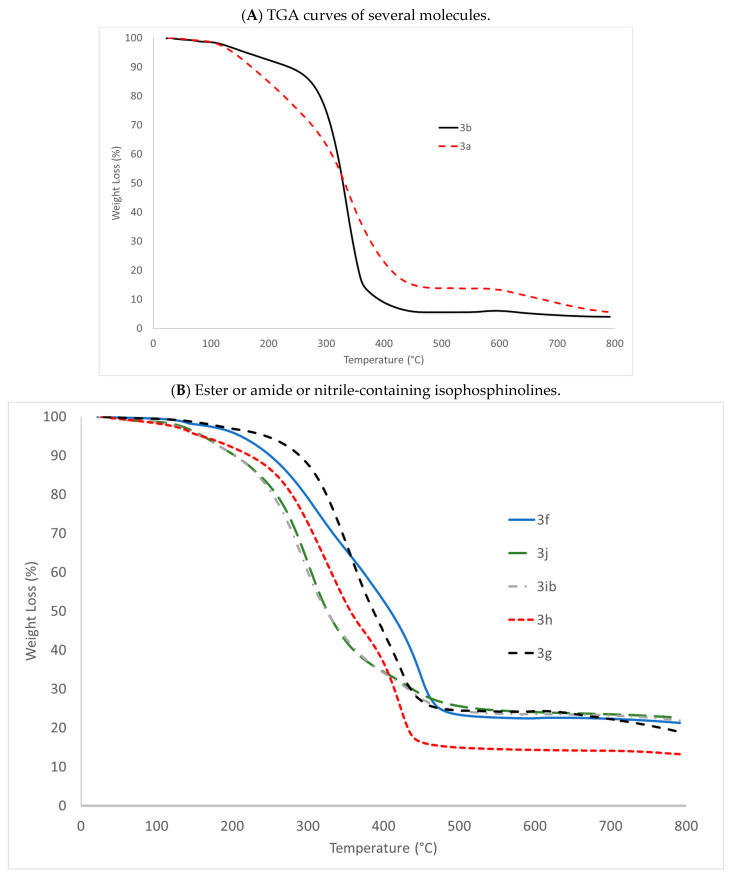

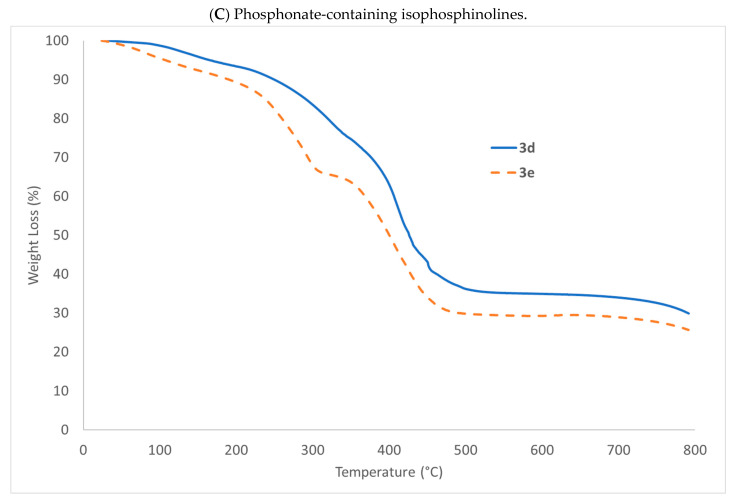
TGA curves of several molecules **3**: (**A**) Phenyl-containing isophosphinolines, (**B**) ester or amide or nitrile-containing isophosphinolines, and (**C**) phosphonate-containing isophosphinolines.

**Figure 2:**
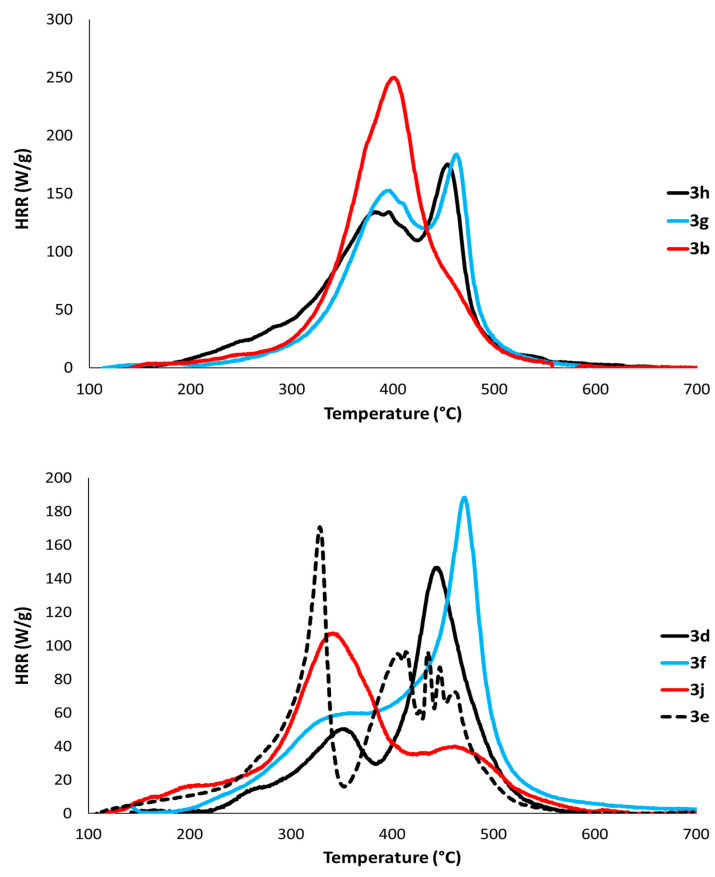
HRR curves of several molecules measured in MCC.

Thermogravimetric analyses under nitrogen flow (anaerobic pyrolysis) and a microscale combustion calorimeter were carried out to assess the pyrolysis and flammability behavior of several isophosphinolines. The data are listed in Table 2 and Table 3, and some thermogravimetric (TG) and heat release rate (HRR) curves are shown in Figure 1 and Figure 2, respectively.

Based on these analyses, three distinct groups of isophosphinolines can be considered. The first group includes compounds **3a** and **3b**, which are molecules with only aromatic substituents. These isophosphinoline derivatives exhibit very low residue at 750 °C in thermogravimetric analysis (TGA) and microscale combustion calorimetry (MCC) around 5 wt%, although their residues are slightly superior compared to triphenylphosphine oxide (TPPO), which was used as a phosphine oxide reference. Consequently, the total heat release was high (23.8 kJ.g^−1^ for **3b**). This observation aligns with previous findings [30] about the contributions of different groups to fire properties. Aromatic groups have effective flame-retardant properties only when they contain a heteroatom or are linked to specific oxygen, nitrogen, or sulfur-bearing groups (such as esters, ketones, amides, or sulfonyls). A phenyl group alone significantly contributes to heat release (35 kJ.g^−1^) and heat release capacity (900 J.g^−1^.K). This explains why the pHRR of compound **3b** was also relatively high (230 W.g^−1^), while its rate of degradation in TGA was higher than for **3a**.

These molecules, **3a** and **3b**, decomposed into one main peak centered at 334–338 °C in TGA and 400 °C in MCC. The heating rate was higher in MCC than in TGA; therefore, peak temperatures were usually delayed. For comparison, the thermal decomposition of TPPO also occurred in a single step with a maximum temperature of 358 °C, close to the temperatures observed for **3a** and **3b**, which were higher than the decomposition temperatures of more oxidized phosphorus-containing analogues (phosphates or phosphonates). The temperature for 5 wt% of mass loss was around 150 °C, and was slightly higher for **3b**, probably due to a higher molecular weight and steric hindrance.

Most articles indicate that phosphine oxides are poor char promoters and have high activity in the gas phase, which is confirmed here. The flame inhibition effect cannot be observed in TGA (where only pyrolysis is measured), nor in MCC (where combustion is forced to be complete). Char promotion may be enhanced by adding functional groups to contribute to a dual mode of action (combining effects in both condensed and gas phases). Increasing residues can limit the formation of combustible gas, and the barrier effect due to a char layer is more expected when the char content is high.

For all molecules tested, except for **3a** and **3b**, the decomposition was more complex with at least two peaks, and the residue content was much higher. Molecules containing substituents such as nitrile, ester, or amide groups exhibited residue contents higher than 20 wt% (except for **3h**–13.8 wt%) in TGA. The residue content in MCC was also significantly improved (20.4 wt% for **3g** and **3ja**).

The Td5% of these molecules was higher, ranging from 160 °C to 245 °C for **3g**. The first main peak of decomposition in TGA was usually close to 300 °C, which was lower than the decomposition peak of **3a** and **3b**. However, the second peak occurred at a much higher temperature than the main decomposition peak of TPPO (at 400 °C). This temperature was increased by a few dozen degrees for other molecules with functional groups carrying heteroatoms.

In MCC, the HRR curves also exhibited two peaks for these nitrogen-containing molecules. The first peak occurred near 400 °C for **3h** and **3g** (pHRR = 128–150 W.g^−1^), and a higher second peak (177–195 W.g^−1^) was observed at a high temperature (452–462 °C). These two peaks were also visible in TGA curves, but were slightly lower compared to those observed in MCC ones. Molecules **3f** and **3ja**, which contained an ester group, exhibited lower thermal stability, with a first peak in the range of 300–350 °C. The residue contents were similar to those obtained in TGA (20 wt%). The THR remained high (22–22.5 kJ.g^−1^), and the heat of complete combustion reached around 28 kJ/g, except for molecule **3ja**, which exhibited the lowest THR. However, its decomposition started at a very low temperature (before 150 °C), which may explain why the heat release was not fully recorded in MCC. For the two first groups, an acceleration of degradation was observed from 300 °C, with a pMLR greater than ≈ 0.4%/°C (based on TGA data).

The third group consisted of molecules **3d** and **3e**, which contained a phosphonate group. Both molecules exhibited the highest residue content in both TGA and MCC (around 30 wt%), confirming that phosphonate groups, with a higher degree of oxidation of the phosphorus atom, enhance charring. Thus, examining products **3e** and **3d**, our team has already observed and confirmed the difference in degradation temperature Td5% between the two phosphonate-containing isophosphinolines [31]. Indeed, the methoxy group is more stable than the ethoxy group. This aligns with the lowest THR observed for both molecules (excluding **3ja**, as previously discussed). Both molecules also exhibited a two-step decomposition, but their thermal stability was relatively lower compared to other isophosphinolines, which is typical for molecules containing phosphate or phosphonate groups. Indeed, in MCC, the first peak was observed in the range 300–350 °C, while the second peak appeared above 400 °C. Moreover, compound **3e**, which had ethyl phosphonate groups, showed premature decomposition with a Td5% close to 100 °C. In MCC, the HRR increased from the beginning of the test, similar to **3ja**.

An interesting observation was made concerning residue. Phosphonate-based isophosphinolines were the only molecules leaving expanded residue at the end of the MCC test. These molecules should be able to promote intumescence, a mode of action very effective in flame retardancy: the formation of a porous, expanded, and thermally stable char layer allows drastic limitations to the heat diffusion from the flame to the underlying material.

Note that the first peak was higher for **3e** compared to **3d**, and for **3ja** compared to **3f**. This tendency was reversed for the second peak, which was higher for **3d** and **3f**. This difference can be ascribed to the presence of ethoxy groups in **3e** and **3ja**, as opposed to the methyl groups found in **3d** and **3f**. These groups likely do not participate in char formation and are probably eliminated at lower temperatures. According to our previous work, the contributions to HRC (heat release capacity, which is equal to the pHRR divided by the heating rate) were 1200 and 1400 J.g^−1^.K for the methylene (CH_2_) and methyl (CH_3_) groups, respectively. Therefore, considering that these groups were involved in the first decomposition peak, the first peak in both TGA and MCC was higher for compounds **3e** and **3j**. Moreover, ethyl groups are generally much less thermally stable than methyl groups, as evidenced by the much lower thermal stability of molecules **3e** and **3ja**.

Some conclusive remarks can be drawn from these results. Isophosphinoline heterocycles are not able to engage in self-charring, despite their highly aromatic structure. However, the presence of functional groups such as esters, amides, nitriles, and especially phosphonates as substituents of the phospha-heterocycle significantly enhances charring. Seibold’s group, when studying the 2,8-dimethyl-phenoxa-phosphin-10-oxide (DPPO) thermal decomposition, have already highlighted the high residue content for phosphine oxides with oxygen synergy [32]. The association of a phosphine oxide with another heteroatom-carrying group induces a synergy that enables action in the condensed phase. This phenomenon is not typically observed in pure phosphine oxide-based *flame*-retardants (FRs). This property makes these molecules promising candidates for future use as FRs in polymers such as epoxies, polyesters, etc. However, this synergy often leads to a more complex decomposition pathway, involving multiple steps at low temperatures. Specifically, the presence of ethyl groups on esters or phosphonates seems to be detrimental to thermal stability. This difference can be attributed to a facilitated internal elimination mechanism (Ei) [27]. Syn-elimination, a thermal process often observed during pyrolysis, occurs through a cyclic 6-membered ring transition state, which is only possible for ethyl-substituted phosphonates.

## 4. X-Ray Crystallography

Single-crystal X-ray diffraction data were collected on a Rigaku XtaLAB Synergy-S, Dualflex diffractometer equipped with a HyPix-6000HE Hybrid Photon Counting (HPC) detector. The unit cell determination and data integration were carried out using the CrysAlisPro package (Version CrysAlisPro 1.171.39.29c, Rigaku OD, 2017) from Oxford Diffraction [33]. Multi-scan correction for absorption was applied. The structures were solved with the SHELXT 2018/2 structure solution program using the Intrinsic Phasing method and refined by the full-matrix least-squares method on F2 with SHELXL 2018/3 [34,35]. Olex2 was used as an interface to the SHELX programs [36]. Non-hydrogen atoms were refined anisotropically. Hydrogen atoms were added in idealized positions and refined using a riding model. Crystal data, data collection, and structure refinement details are provided in the electronic supporting information and the corresponding CIF files. CCDC-2386195 contains the supplementary crystallographic data for this paper. These data can be obtained free of charge from The Cambridge Crystallographic Data Centre via “https://www.ccdc.cam.ac.uk/mystructures/structuredetails/81620710-4b7a-ef11-96c9-00505695281c (deposited and accessed on 24 September 2024)”. The monocrystals, which generally were difficult to obtain, were in the case of compound **3l** grown from anhydrous ethyl acetate and DCM (3:7) by slow evaporation over approximately ten days after a large screening of gradients of all the usual solvents. Unfortunately, the application of similar conditions to other derivatives reported in this paper, specifically **3d**, **3k**, and **3m**, failed to obtain their X-ray crystal structure. The solid compounds obtained in all the latter cases were found to be amorphous.

**Figure 3:**
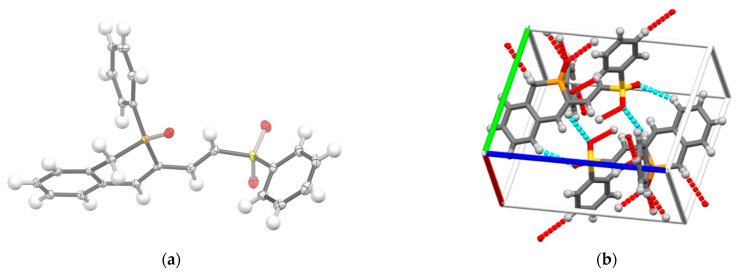
(**a**) X-ray molecular structure with atom labelling and thermal ellipsoids at 50% level for **3l**. (**b**) Packing structure in the crystal, together with the crystal unit cell, indicating 4 H-bonds in blue-sky between two molecules of **3l**.

For crystal, structural, and refinement statistics, see the ESI. The important facts are shown in part (a) and (b) of Figure 3. The crystal of the titled molecule **3l** is governed by an intermolecular O_1_(of SO_2_ group)–H(C_6_ of monosubstituted phenyl group) and O_2_(of SO_2_ group)–H(of C_18_ of monosubstituted phenyl group) interactions. There are four of these H-bonds (S=O–H-Csp^2^) in the crystal unit cell, but only two types, which ensures the cohesion of only two molecules of **3l**.

## 5. Conclusions

These preliminary results represent the first investigation on the regioselective C2-functionalization of a phospha-heterocycle, 2-phenyl-1*H*-isophosphinoline 2-oxide (**1**) with olefins containing different substituents. The coupling products **3a**–**m** were obtained using a catalytic system comprising both Ag_2_CO_3_ and palladium(II) acetate, with yields ranging from 21% to 87%. The C-H functionalization occurred selectively at the alpha position of the 2-phenyl-1*H*-isophosphinoline 2-oxide core forming from monosubstituted alkenes exclusively, then *E*-isomers proved by X-ray analysis of product **3l** (CCDC-2386195) as representative example. In a second investigation, the flame-retardant properties of selected molecules were examined. It was clearly demonstrated that the association of a phosphine oxide with another heteroatom-carrying group has positive synergistic effects enabling action in the condensed phase that is not seen in pure phosphine oxide FRs.

## 6. Material and Methods

### 6.1. Analytical Data

^1^H, ^13^C{1H}, and ^31P^{1H} NMR spectra were recorded with a Bruker Avance 400 MHz spectrometer. The chemical shifts were recorded as follows: the chemical shift was measured in parts per million (ppm), and the coupling constants (*J*) were reported in Hz. Multiplicities were reported as singlet (s), broad singlet (bs), doublet (d), triplet (t), quartet (q), quintet (quin), and multiplet (m). High resolution mass spectroscopic (HRMS) analysis was performed on a Xevo G2 Q TOF spectrometer using the electrospray method by the Laboratoire de Mesures Physiques of the University of Montpellier. All reactions were run under an atmosphere of nitrogen using standard Schlenk techniques otherwise stated.

### 6.2. Thermogravimetric Analysis (TGA)

Thermogravimetric analyses (TGA) were performed using a TGA Q50 (TA instrument) at a heating rate of 20 °C.min^−1^. Two to eight milligrams of each sample was placed on a platinum pan and heated from room temperature to 800 °C under nitrogen flow (60 mL.min^−1^). The temperature at 5% weight loss, the temperature at the maximum rate of weight loss, the rate at the maximum degradation temperature, and the char yield at 750 °C were measured.

### 6.3. Microscale Combustion Calorimetry (MCC)

A microscale combustion calorimeter (also called a pyrolysis-combustion flow calorimeter or PCFC) from Fire Testing Technologies was used to assess the flammability of some isophosphinolines. The samples (2–5 mg) first underwent an anaerobic pyrolysis step (1 K/s) from 100 to 750 °C under nitrogen flow (100 mL/min). The gases from pyrolysis were sent to a combustor, where they were fully oxidized at 900 °C in the presence of a nitrogen–oxygen mixture (80/20, with oxygen in excess). The heat release rate was calculated according to the well-known Huggett relation (1 g of oxygen consumed corresponds to 13.1 kJ of energy released). The heat release rate was plotted versus the pyrolysis temperature. The residue content was also measured at the end of the test, and the heat of complete combustion (Δh in kJ.g^−1^) was calculated as the ratio between the total heat release (i.e., the area under the HRR curve versus temperature of pyrolysis) and the residue fraction.

### 6.4. General Procedures

#### A Typical Procedure for the Synthesis of C-H Alkenated Products **3a**–**m**

In a sealed tube, the 2-phenyl-1*H*-isophosphinoline oxide (**1**, 0.416 mmol, 1 eq.), a suitable alkene (0.486 mmol, 1.17 eq.) and silver carbonate (0.486 mmol, 1.17 eq.) were dissolved in pivalic acid 4 mL (solid at rt, and heated with a hit gun in order to sample). Pd(OAc)_2_ (10 mol%, 0.0486 mmol, 0.117 eq.) was then added, and the reaction mixture was stirred at 90 °C for 20 h. After cooling to rt, the reaction mixture was poured into a saturated solution of K_2_CO_3_ (20 mL) and left to stir for 20 h. Then, after extraction of the solution with DCM (3 × 15 mL), the collected organic layers were dried over MgSO_4_, filtered, and evaporated under reduced pressure. Purification on flash chromatography of the residue was performed to provide the expected compounds **3a**–**m** in appreciable to good yields.

### 6.5. Experimental Data

(*E*)-2-Phenyl-3-styryl-1*H*-isophosphinoline 2-oxide (**3a**). ^31^P NMR (162 MHz, CDCl_3_) δ 23.09. ^1^H NMR (400 MHz, CDCl_3_) δ 7.98–7.64 (m, 1H), 7.57–7.06 (m, 9H), 6.90 (ddd, *J* = 20.3, 16.4, 0.9 Hz, 1H), 3.76 (ddd, *J* = 22.2, 17.1, 1.1 Hz, 1H), 3.57–3.19 (m, 1H). ^13^C NMR (101 MHz, CDCl_3_) δ 141.89 (d, *J* = 6.1 Hz), 137.14, 134.13 (d, *J* = 5.5 Hz), 132.70 (d, *J* = 13.6 Hz), 132.20 (d, *J* = 2.8 Hz), 130.56 (d, *J* = 6.1 Hz), 130.49 (d, *J* = 2.7 Hz), 130.34 (d, *J* = 10.1 Hz), 128.82 (d, *J* = 12.0 Hz), 128.07 (d, *J* = 13.1 Hz), 127.72 (d, *J* = 187.3 Hz), 126.75, 35.23 (d, *J* = 70.6 Hz). HRMS: *m*/*z* calcd for C_23_H_20_OP 343.12463 [M + H]^+^, found 343.12466. Yield: 57%; 82 mg; eluent: ethyl acetate/petroleum ether (7:3).

(*Z*)-3-(1,2-Diphenylvinyl)-2-phenyl-1*H*-isophosphinoline 2-oxide (**3b**). ^31^P NMR (162 MHz, CDCl_3_) δ 22.68. ^1^H NMR (400 MHz, CDCl_3_) δ 8.19–6.35 (m, 21H), 3.76 (dd, *J* = 21.1, 17.1 Hz, 1H), 3.44 (dd, *J* = 17.2, 12.0 Hz, 1H). ^13^C NMR (101 MHz, CDCl_3_) δ 142.87 (d, *J* = 6.5 Hz), 136.73, 132.95 (d, *J* = 5.9 Hz), 132.04, 130.94, 130.58, 130.48, 130.30, 130.16, 129.90, 129.57, 129.44, 129.36, 129.06, 128.84, 128.72, 128.38, 128.01, 127.84, 127.15, 35.75 (d, *J* = 71.0 Hz). HRMS: *m*/*z* calcd for C_29_H_23_OP 419.15613 [M + H]^+^, found 419.15601. Yield: 29%; 51 mg; eluent: ethyl acetate/petroleum ether (7:3).

3-(Cyclohex-2-en-1-yl)-2-phenyl-1H-isophosphinoline 2-oxide (**3c**). ^31^P NMR (162 MHz, CDCl_3_) 2 diastereomers: δ 24.17 (36%), 22.9 (64%). ^1^H NMR (400 MHz, CDCl_3_) δ 7.69 (m, 10H), 6.09–5.35 (m, 2H), 3.81–3.52 (m, 1H), 3.41–3.20 (m, 1H), 2.15–0.85 (m, 7H). ^13^C NMR (101 MHz, CDCl_3_) δ 141.26 (d, *J* = 6.7 Hz), 140.84 (d, *J* = 6.8 Hz), 137.63 (d, *J* = 87.2 Hz), 137.01 (d, *J* = 88.0 Hz), 133.97–132.32 (m), 132.45–127.58 (m), 36.55 (d, *J* = 8.0 Hz), 35.67 (d, *J* = 7.4 Hz), 34.79 (d, *J* = 69.6 Hz), 34.73 (d, *J* = 69.5 Hz), 30.23 (d, *J* = 2.2 Hz), 29.82, 29.25 (d, *J* = 2.5 Hz), 24.99, 20.03, 19.96. HRMS: *m*/*z* calcd for C_21_H_22_OP 321.14028 [M + H]^+^, found 321.14038. Yield: 38; 51 mg; eluent: ethyl acetate/petroleum ether (9:1).

Dimethyl (*E*)-(2-(2-oxido-2-phenyl-1*H*-isophosphinolin-3-yl)vinyl)phosphonate (**3d**). ^31^P NMR (162 MHz, CDCl_3_) δ 21.34 (d, *J* = 3.4 Hz), 21.05 (d, *J* = 3.4 Hz). ^1^H NMR (400 MHz, CDCl_3_) δ 7.66–7.55 (m, 2H), 7.49–7.09 (m, 10H), 6.27 (td, *J* = 17.7, 1.3 Hz, 1H), 3.75–3.65 (m, 1H), 3.61 (d, *J* = 11.2 Hz, 3H), 3.42 (d, *J* = 11.2 Hz, 3H), 3.35 (dd, *J* = 17.3, 12.4 Hz, 1H). ^13^C NMR (101 MHz, CDCl_3_) δ 148.61 (d, *J* = 5.9 Hz), 147.09, 132.50 (d, *J* = 2.9 Hz), 132.39, 131.51 (d, *J* = 13.3 Hz), 130.95, 130.90, 130.82, 130.71, 130.39, 130.29, 128.99, 128.87, 128.60, 128.25, 127.84 (d, *J* = 25.3 Hz), 118.90 (d, *J* = 4.5 Hz), 117.05 (d, *J* = 4.4 Hz). HRMS: *m*/*z* calcd for C_19_H_21_O_4_P_2_ 375.09096 [M + H]^+^, found 375.09100. Yield: 50%; 78 mg; eluent: ethyl acetate/petroleum ether (97.5:2.5).

Diethyl (*E*)-(2-(2-oxido-2-phenyl-1*H*-isophosphinolin-3-yl)vinyl)phosphonate (**3e**). ^31^P NMR (162 MHz, CDCl_3_) δ 21.21 (d, *J* = 3.4 Hz), 18.05 (d, *J* = 3.4 Hz). ^1^H NMR (400 MHz, CDCl_3_) δ 7.79–7.62 (m, 2H), 7.55–7.09 (m, 9H), 6.53–6.23 (m, 1H), 4.09–3.96 (m, 2H), 3.89–3.66 (m, 3H), 3.40 (m, *J* = 12.3, 0.9 Hz, 1H), 1.29 (td, *J* = 7.1, 0.6 Hz, 3H), 1.10 (td, *J* = 7.0, 0.5 Hz, 3H). ^13^C NMR (101 MHz, CDCl_3_) δ 148.27, 146.27 (t, *J* = 6.5 Hz), 132.67, 132.45, 131.74 (d, *J* = 2.3 Hz), 131.56, 130.98, 130.46 (d, *J* = 10.0 Hz), 128.93 (d, *J* = 12.2 Hz), 128.26, 120.66 (d, *J* = 4.5 Hz), 118.82 (d, *J* = 4.4 Hz), 61.92 (dd, *J* = 23.4, 5.3 Hz), 35.23 (d, *J* = 70.7 Hz), 16.28 (s). HRMS: *m*/*z* calcd for C_21_H_25_OP 403.12226 [M + H]^+^, found 403.12234. Yield: 87%; 145 mg; eluent: ethyl acetate/MeOH (95:5).

Methyl (*E*)-3-(2-oxido-2-phenyl-1*H*-isophosphinolin-3-yl)acrylate (**3f**). ^31^P NMR (162 MHz, CDCl_3_) δ 21.20. ^1^H NMR (400 MHz, CDCl_3_) δ 7.78–7.63 (m, 2H), 7.58–7.04 (m, 9H), 6.55 (d, *J* = 16.1 Hz, 1H), 3.76 (d, *J* = 1.2 Hz, 1H), 3.69 (s, 3H), 3.41 (d, *J* = 17.3 Hz, 1H). ^13^C NMR (101 MHz, CDCl_3_) δ^13^C NMR (101 MHz, CDCl_3_) δ 167.29, 148.57 (d, *J* = 5.8 Hz), 142.26 (d, *J* = 5.8 Hz), 132.53 (d, *J* = 2.8 Hz), 131.81 (d, *J* = 13.1 Hz), 131.65 (d, *J* = 2.2 Hz), 131.20 (d, *J* = 7.1 Hz), 130.81, 130.80 (d, *J* = 11.2 Hz), 130.28 (d, *J* = 10.1 Hz), 128.99 (d, *J* = 12.2 Hz), 128.42 (d, *J* = 88.9 Hz), 128.27, 51.78, 35.14 (d, *J* = 70.8 Hz). HRMS: *m*/*z* calcd for C_19_H_18_O_3_P 325.09881 [M + H]^+^, found 325.09885. Yield: 59%; 81 mg; eluent: ethyl acetate/petroleum ether (9:1).

(*E*)-*N,N*-Dimethyl-3-(2-oxido-2-phenyl-1*H*-isophosphinolin-3-yl)acrylamide (**3g**). ^31^P NMR (162 MHz, CDCl_3_) δ 21.94. ^1^H NMR (400 MHz, CDCl_3_) δ 7.67 (ddd, *J* = 12.2, 8.4, 1.4 Hz, 2H), 7.53–7.20 (m, 8H), 7.15 (d, *J* = 7.3 Hz, 1H), 7.02 (dd, *J* = 15.4, 1.3 Hz, 1H), 3.74 (dd, *J* = 21.9, 17.2 Hz, 1H), 3.40 (dd, *J* = 17.2, 12.6 Hz, 1H), 2.93 (s, 3H), 2.87 (s, 3H). ^13^C NMR (101 MHz, CDCl_3_) δ 147.71 (d, *J* = 6.1 Hz), 139.77 (d, *J* = 5.7 Hz), 132.55 (d, *J* = 88.3 Hz), 132.36 (d, *J* = 2.9 Hz), 131.99, 131.42 (d, *J* = 2.6 Hz), 130.62, 128.91 (d, *J* = 12.1 Hz), 128.57 (d, *J* = 11.8 Hz), 128.19, 122.72 (d, *J* = 4.1 Hz), 36.58 (d, *J* = 144.0 Hz), 35.17 (d, *J* = 70.6 Hz). HRMS: *m*/*z* calcd for C_20_H_21_O_2_NP 338.13044 [M + H]^+^, found 338.13052. Yield: 25%; 35 mg; eluent: ethyl acetate/MeOH (95:5).

(*E*)-3-(2-Oxido-2-phenyl-1*H*-isophosphinolin-3-yl)acrylonitrile (**3h**). ^31^P NMR (162 MHz, CDCl_3_) δ 21.75. 1H NMR (400 MHz, CDCl_3_) δ 7.69–7.49 (m, 3H), 7.47–7.30 (m, 6H), 7.23–7.01 (m, 2H), 6.24 (d, J = 16.9 Hz, 1H), 3.82 (ddd, J = 23.0, 17.3, 1.0 Hz, 1H), 3.43 (dd, J = 17.3, 13.1 Hz, 1H). ^13^C NMR (101 MHz, CDCl_3_) δ 148.93 (d, *J* = 5.3 Hz), 148.09 (d, *J* = 5.8 Hz), 132.87 (d, *J* = 2.9 Hz), 132.02 (d, *J* = 2.3 Hz), 131.59 (d, *J* = 64.3 Hz), 131.37, 131.11 (d, *J* = 6.9 Hz), 130.89 (d, *J* = 11.5 Hz), 130.02 (d, *J* = 10.1 Hz), 129.12 (d, *J* = 12.3 Hz), 128.47, 128.06, 127.19, 118.00, 101.65 (d, *J* = 4.9 Hz), 34.80 (d, *J* = 71.1 Hz). HRMS: *m*/*z* calcd for C_18_H_15_ONP 292.08858 [M + H]^+^, found 292.08859. Yield: 44%; 54 mg; eluent: DCM/MeOH (95:5).

Methyl 2-methyl-3-(2-oxido-2-phenyl-1*H*-isophosphinolin-3-yl)acrylate (**3ia**). ^31^P NMR (162 MHz, CDCl_3_) δ 21.29 (s). ^1^H NMR (400 MHz, CDCl_3_) δ 7.70–7.65 (m, 2H), 7.50 (d, *J* = 7.5 Hz, 1H), 7.41 (t, *J* = 7.4 Hz, 2H), 7.36–7.26 (m, 5H), 7.20 (d, *J* = 7.0 Hz, 1H), 3.79 (d, *J* = 17.3 Hz, 1H), 3.70 (s, 3H), 3.44–3.39 (m, 1H), 2.05 (d, *J* = 1.5 Hz, 3H). ^13^C NMR (101 MHz, CDCl_3_) δ 184.02 (s), 168.16 (s), 145.12 (d, *J* = 5.1 Hz), 133.59 (d, *J* = 6.9 Hz), 132.51 (d, *J* = 2.8 Hz), 131.73 (dd, *J* = 12.0, 6.2 Hz), 131.13 (d, *J* = 2.5 Hz), 131.00 (d, *J* = 10.9 Hz), 130.63 (d, *J* = 9.9 Hz), 130.58 (s), 130.21 (s), 129.44 (d, *J* = 7.0 Hz), 128.90 (d, *J* = 12.2 Hz), 128.16 (s), 52.19 (s), 36.44–32.84 (m), 14.88 (s). HRMS: *m*/*z* calcd for C_20_H_19_O_3_P 339,1145 [M + H]^+^, found 339,1144. Yield: 4%; 63 mg; eluent: ethyl acetate/MeOH (97.5:2.5).

Methyl 2-((2-oxido-2-phenyl-1*H*-isophosphinolin-3-yl)methyl)acrylate (**3ib**). ^31^P NMR (162 MHz, CDCl_3_) δ 23.13. ^1^H NMR (400 MHz, CDCl_3_) δ 7.64 (ddd, *J* = 11.9, 8.3, 1.4 Hz, 2H), δ 7.53–7.34 (m, 3H), 7.31–7.17 (m, 4H), 7.15–6.92 (m, 2H), 6.23 (d, *J* = 1.2 Hz, 1H), 5.68 (d, *J* = 1.3 Hz, 1H), 3.60 (s, 3H), 3.40 (d, *J* = 10.6 Hz, 2H), 3.33 (dd, *J* = 17.2, 12.5 Hz, 1H). ^13^C NMR (101 MHz, CDCl_3_) δ 142.19 (d, *J* = 6.4 Hz), 132.24 (d, *J* = 2.9 Hz), 130.81, 130.71, 130.62, 130.29, 129.61, 129.36, 128.72 (d, *J* = 12.0 Hz), 127.95, 51.97, 34.40 (d, *J* = 70.1 Hz), 33.50 (d, *J* = 8.2 Hz). HRMS: *m*/*z* calcd for C_20_H_20_O_3_P 339.11446 [M + H]^+^, found 339.11450. Yield: 10%; 143 mg; eluent: ethyl acetate/MeOH (97.5:2.5).

Methyl 3-(2-oxido-2-phenyl-1*H*-isophosphinolin-3-yl)-2-((2-oxido-2-phenyl-1*H*-isophosphinolin-3-yl)methyl)acrylate (**3ic**). ^31^P NMR (162 MHz, Chloroform-*d*) δ 22.84 (d, *J* = 24.9 Hz), 21.86 (d, *J* = 18.3 Hz). ^1^H NMR (400 MHz, CDCl_3_) δ 7.80–7.52 (m, 5H), 7.52–7.30 (m, 6H), 7.30–7.08 (m, 7H), 6.86 (dd, *J* = 72.0, 33.0 Hz, 1H), 3.76–3.63 (m, 3H), 3.64–3.54 (m, 2H), 3.55–3.22 (m, 3H), 1.24 (t, *J* = 7.1 Hz, 3H).^13^C NMR (101 MHz, CDCl_3_) δ 167.14 (d, *J* = 10.1 Hz), 144.80 (dd, *J* = 42.8, 4.2 Hz), 140.41 (dd, *J* = 12.0, 6.1 Hz), 136.47 (dd, *J* = 24.4, 7.7 Hz), 132.60–132.21 (m), 132.22–131.96 (m), 132.27–131.41 (m), 131.11 (dd, *J* = 12.9, 6.6 Hz), 131.01–130.24 (m), 130.40–129.64 (m), 129.53 (s), 129.23–128.59 (m), 128.36 (s), 128.23 (d, *J* = 11.4 Hz), 127.89 (d, *J* = 10.7 Hz), 127.21 (d, *J* = 52.4 Hz), 60.43 (s), 52.19 (d, *J* = 1.9 Hz), 35.21–33.21 (m), 29.87–28.84 (m). HRMS: *m*/*z* calcd for C_35_H_30_O_4_P_2_ 577.1692 [M + H]^+^, found.577.1692. Yield: 25%; 343 mg; eluent: ethyl acetate/MeOH (97.5:2.5).

Ethyl 2-((2-oxido-2-phenyl-1*H*-isophosphinolin-3-yl)methyl)acrylate (**3ja**). ^31^P NMR (162 MHz, CDCl_3_) δ 21.11 (s). ^1^H NMR (400 MHz, CDCl_3_) δ 7.73–7.64 (m, 2H), 7.52 (ddt, *J* = 6.7, 4.2, 1.4 Hz, 1H), 7.47–7.39 (m, 2H), 7.37–7.26 (m, 5H), 7.21 (d, *J* = 6.9 Hz, 1H), 4.22–4.06 (m, 2H), 3.72 (dd, *J* = 20.8, 17.2 Hz, 1H), 3.40 (dd, *J* = 17.2, 11.7 Hz, 1H), 2.06 (t, *J* = 1.6 Hz, 3H), 1.24 (t, *J* = 7.1 Hz, 3H). ^13^C NMR (101 MHz, CDCl_3_) δ 167.86 (s), 145.01 (d, *J* = 4.9 Hz), 133.62 (d, *J* = 6.9 Hz), 132.56 (d, *J* = 2.7 Hz), 132.37–131.82 (m), 131.16 (d, *J* = 12.5 Hz), 130.85 (d, *J* = 9.8 Hz), 130.66 (s), 130.28 (s), 130.14 (s), 129.25 (s), 129.02 (d, *J* = 12.1 Hz), 128.29 (s), 61.22 (s), 34.89 (d, *J* = 70.1 Hz), 14.75 (d, *J* = 56.1 Hz). HRMS: *m*/*z* calcd for C_21_H_22_O_3_P 353.13011 [M + H]^+^, found 353.13019. Yield: 4%; 12 mg; eluent: ethyl acetate/MeOH (97.5:2.5).

Ethyl 2-((2-oxido-2-phenyl-1*H*-isophosphinolin-3-yl)methyl)acrylate (**3jb**). ^31^P NMR (162 MHz, Chloroform-*d*) δ 23.10. ^1^H NMR (400 MHz, Chloroform-*d*) δ 7.65 (ddd, *J* = 11.9, 8.3, 1.4 Hz, 2H), 7.47 (m, 1H), 7.43–7.34 (m, 2H), 7.31–7.16 (m, 3H), 7.15–6.99 (m, 2H), 6.23 (d, *J* = 1.3 Hz, 1H), 5.68 (d, *J* = 1.2 Hz, 1H), 4.08 (m, 2H), 3.67 (dd, *J* = 21.7, 17.1 Hz, 1H), 3.41 (d, *J* = 10.6 Hz, 2H), 3.33 (dd, *J* = 17.1, 12.4 Hz, 1H), 1.19 (t, *J* = 7.1 Hz, 3H). ^13^C NMR (101 MHz, Chloroform-*d*) δ 142.00 (d, *J* = 6.3 Hz), 132.42 (d, *J* = 14.8 Hz), 132.16, 131.55, 130.80, 130.71, 130.67 (d, *J* = 72.1 Hz), 130.62, 130.21, 129.07, 129.01, 128.76, 128.64, 127.93, 60.87, 35.24–33.11 (m), 14.27. HRMS: *m*/*z* calcd for C_21_H_22_O_3_P 353.13011 [M + H]^+^, found 353.13019. Yield: 31%; 91 mg; eluent: ethyl acetate/MeOH (97.5:2.5).

Ethyl 3-(2-oxido-2-phenyl-1*H*-isophosphinolin-3-yl)-2-((2-oxido-2-phenyl-1*H*-isophosphinolin-3-yl)methyl)acrylate (**3jc**). ^31^P NMR (162 MHz, CDCl_3_) δ 22.69 (d, *J* = 25.4 Hz), 21.70 (d, *J* = 8.4 Hz). ^1^H NMR (400 MHz, CDCl_3_) δ 7.80–7.58 (m, 4H), 7.58–7.46 (m, 2H), 7.46–7.27 (m, 7H), 7.26–7.10 (m, 4H), 6.87 (dd, *J* = 63.3, 33.0 Hz, 1H), 4.12 (dd, *J* = 14.4, 7.2 Hz, 2H), 4.08–4.01 (m, 1H), 3.67 (ddd, *J* = 21.2, 17.8, 8.3 Hz, 3H), 3.55–3.29 (m, 2H), 1.28–1.22 (m, 3H), 1.16 (dt, *J* = 9.1, 7.1 Hz, 3H). ^13^C NMR (101 MHz, CDCl_3_) δ 166.71 (d, *J* = 9.9 Hz), 144.84 (t, *J* = 19.1 Hz), 144.52 (d, *J* = 4.2 Hz), 140.80–139.93 (m), 136.21 (dd, *J* = 26.1, 7.7 Hz), 132.28 (d, *J* = 5.6 Hz), 132.11 (d, *J* = 3.3 Hz), 132.05 (s), 131.08–130.07 (m), 129.83 (dt, *J* = 14.5, 7.2 Hz), 129.27–128.59 (m), 128.24 (d, *J* = 11.9 Hz), 128.02 (d, *J* = 3.9 Hz), 127.89 (d, *J* = 10.2 Hz), 127.41 (d, *J* = 53.6 Hz), 61.12 (d, *J* = 4.2 Hz), 60.41 (s), 35.30–33.62 (m), 29.91–29.01 (m), 21.08 (s), 15.11–13.39 (m). HRMS: *m*/*z* calcd for C_36_H_33_O_4_P_2_ 591.1849.13011 [M + H]^+^, found 591.1847. Yield: 27%; 78 mg; eluent: ethyl acetate/ MeOH (97.5:2.5).

(*E*)-*N*-(4-Methoxybenzyl)-3-(2-oxido-2-phenyl-1H-isophosphinolin-3-yl)acrylamide (**3k**). ^31^P NMR (162 MHz, CDCl_3_) δ 23.57. ^1^H NMR (400 MHz, CDCl_3_) δ 7.59 (dd, *J* = 12.3, 7.6 Hz, 2H), 7.47 (td, *J* = 15.4, 14.9, 10.2 Hz, 3H), 7.34 (m, 5H), 7.16 (d, *J* = 8.2 Hz, 2H), 7.11 (d, *J* = 7.5 Hz, 1H), 6.80 (d, *J* = 8.2 Hz, 2fH), 6.73 (d, *J* = 15.5 Hz, 1H), 6.29 (s, 1H), 4.45 (dd, *J* = 14.7, 5.8 Hz, 1H), 4.35 (dd, *J* = 14.6, 5.4 Hz, 1H), 3.75 (s, 3H), 3.71–3.58 (m, 1H), 3.30 (dd, *J* = 17.2, 13.5 Hz, 1H). ^13^C NMR (101 MHz, CDCl_3_) δ 165.6, 159.0, 147.8–147.7 (d), 138.7 (d), 132.5 (d), 132.1–132 (d), 131.40 (d), 131.0–130.9 (d), 130.6, 130.5, 130.40, 130.2–130.1 (d), 129.4, 129.0–128.8 (d), 128.2, 125.9, 114.1, 55.33, 43.37, 29.83. HRMS (APPI+): Calculated for C_26_H_24_NO_3_P [M+H]+ 430.1566 found 430.1561. Yield 31%, 28 mg; eluent: ethyl acetate/dichloromethane (1:1).

(*E*)-2-Phenyl-3-(2-(phenylsulfonyl)vinyl)-1*H*-isophosphinoline 2-oxide (**3l**). ^31^P NMR (162 MHz, CDCl_3_) δ 21.77. ^1^H NMR (400 MHz, CDCl_3_) δ 7.80 (d, *J* = 7.7 Hz, 2H), 7.56 (dd, *J* = 11.2, 5.8 Hz, 4H), 7.46 (q, *J* = 6.5, 5.0 Hz, 5H), 7.35 (qd, *J* = 11.0, 10.0, 7.4 Hz, 5H), 7.16 (m, 2H), 3.76 (dd, *J* = 22.5, 17.2 Hz, 1H), 3.41 (dd, *J* = 17.3, 12.9 Hz, 1H). ^13^C NMR (101 MHz, CDCl_3_) δ δ 150.5–150.4 (d), 140.5, 139.8–139.7 (d), 133.4, 132.8–132.7 (d), 132.0 (d), 131.8, 131.5, 131.4, 131.3 (d), 131.0–130.8 (d), 130.1–130.0 (d), 129.3, 129.1–129.0 (d), 128.4, 128.0, 127.0, 126.1, 35.1–34.4 (d)f. HRMS (APPI+): Calculated for C_23_H_19_O_3_PS [M+H]+ 407.0865 found 407.0862. Yield 37%, 31 mg; eluent: ethyl acetate/DCM (3:7).

(*E*)-2-(2-(2-Oxido-2-phenyl-1*H*-isophosphinolin-3-yl)vinyl)isoindoline-1,3-dione (**3m**). ^31^P NMR (162 MHz, CDCl_3_) δ 22.33. ^1^H NMR (400 MHz, CDCl_3_) δ 7.83 (dt, *J* = 7.3, 3.7 Hz, 2H), 7.77–7.70 (m, 5H), 7.62 (d, *J* = 15.2 Hz, 1H), 7.53–7.46 (m, 2H), 7.41 (td, *J* = 7.6, 2.9 Hz, 2H), 7.30 (d, *J* = 4.0 Hz, 2H), 7.23 (d, *J* = 3.2 Hz, 1H), 7.16 (d, *J* = 7.5 Hz, 1H), 3.79 (dd, *J* = 22.5, 17.1 Hz, 1H), 3.43 (dd, *J* = 17.2, 12.8 Hz, 1H). ^13^C NMR (101 MHz, CDCl_3_) δ 168.1, 146.2, 142.0–141.90 (d), 134.7, 134.4, 133.0, 132.7–132.6 (d), 132.3 (d), 132.0, 131.8, 130.6 (d), 130.5–130.4 (d), 130.3 (d), 129.6, 129.3, 129.0–128.8 (d), 128.1, 123.8, 123.7, 122.2 (d), 118.5 (d), 35.4–34.7 (d). HRMS (APPI+): Calculated for C_25_H_18_NO_3_P [M+H]+ 412.1097 found 412.1091. Yield 21%, 20 mg, eluent: ethyl acetate/DCM (2:8).

## Figures and Tables

**Table 1:** Regioselective C2 functionalization of 1*H*-isophosphinoline 2-oxide (**1**) with alkenes.

Entry	Alkene 2	Alkene 2 (Structure)	Product 3	Product 3 ^1^ (Structure)	Yield ^2^ (in %)
1	**2a**	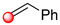	**3a**	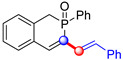	57
2	**2b**	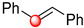	**3b**	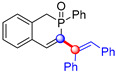	28
3	**2c**	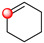	**3c**	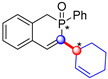	38
4	**2d**	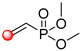	**3d**	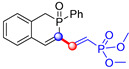	50
5	**2e**	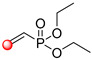	**3e**	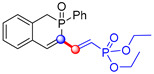	87
6	**2f**	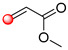	**3f**	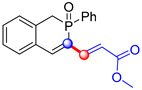	59
7	**2g**	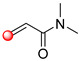	**3g**	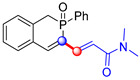	25
8	**2h**	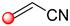	**3h**	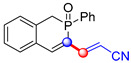	44
9	**2i**	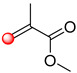	**3ia** **3ib** **3ic**	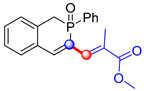 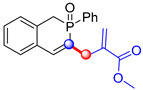 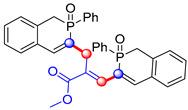	4 10 25
10	**2j**	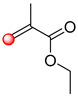	**3ja** **3jb** **3jc**	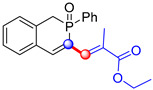 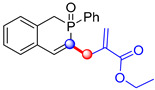 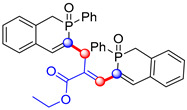	4 31 27
11	**2k**	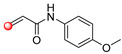	**3k**	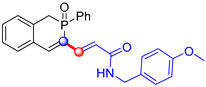	31
12	**2l**	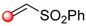	**3l**	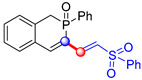 see X-ray	37
**13**	**2m**	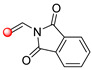	**3m**	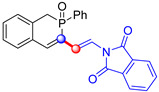	21

^1^ Only the C2-functionalized isomer was produced. ^2^ Yields are reported after isolation and purification by silica gel flash column chromatography.

**Table 2:** TGA values of some interesting functionalized isophosphinolines.

Sample	Td5% (°C)	Td Max (°C)	pMLR ^1^ (%/°C)	Residue at 750 °C (in %)
**3a**	140	≈210 ^2^	0.1821	6.6
		338	0.5131	
**3b**	165	334	1.221	4.1
**3d**	169	324	0.1985	32.5
		411	0.5572	
		450	0.3703	
**3e**	106	292	0.3403	27.7
		405	0.3719	
**3f**	211	310	0.2780	21.8
		449	0.5421	
**3g**	245	358	0.5269	20.6
		423	0.4662	
**3h**	162	327	0.4218	13.8
		421	0.5884	
**3ib**	161	293	0.5035	22.7
		437	0.1481	
**3ja**	160	178	0.1236	23.1
		300	0.5313	
		428	0.1237	

^1^ pMLR: peak of mass loss rate. ^2^ Shoulder.

**Table 3:** Main data measured in PCFC for several molecules.

Sample	pHRR1 (W.g^−1^)	TpHRR1 (°C)	pHRR2 (W.g^−1^)	TpHRR2 (°C)	THR (kJ.g^−1^)	Residue (wt%)	ΔH (kJ.g^−1^)
**3b**	230	400	-	-	23.8	3.9	24.8
**3d**	51	353	178	445	16.2	32.4	24.0
**3e**	175	329	98	413	19.1	32.5	28.3
**3f**	59	351	188	472	20.7	25.7	27.9
**3g**	150	395	195	462	22	20.4	27.6
**3h**	128	381	177	452	22.5	20.4	28.3
**3ja**	118	317	40	463	14.5 ^1^	24	19.1 ^1^

^1^ Doubtful data, due to early decomposition.

## Data Availability

Data contained within the article or Supplementary Materials.

## Supplementary Materials

The following supporting information can be downloaded at: https://www.mdpi.com/article/10.3390/molecules29215104/s1, Figures S1–S17: ^31^P{1H}, ^1^H and ^13C^{1H} NMR and HRMS spectra of the synthesized compounds.
