# Supplementary material for: Palladium-Catalyzed C-H Functionalization and Flame-Retardant Properties of Isophosphinolines
Source: Molecules. 2024 Oct 29;29(21):5104. doi: 10.3390/molecules29215104 (PMC11547386; doi:10.3390/molecules29215104)
Supplement: Supplementary file 1 [file molecules-29-05104-s001.zip › molecules-3256573-supplementary.pdf]

Palladium-Catalyzed C-H functionalization and flame-retardant properties of isophosphinolines

Karen-Pacelye Mengue Me Ndong[1,2], Mina Hariri[1,3], Gabin Mwande-Maguene[2], Jacques Lebibi[2], Fatemeh Darvish[3], Christine Safi[4], Kouceila Abdelli[4], Adam Daïch[4], Claire Negrell[1], Rodolphe Sonnier[5], Loic Dumazert[5], Abdou Rachid Issaka Ibrahim[6], Ilagouma Amadou Tidjani[6], David Virieux[1], Tahar Ayad[1]*and Jean-Luc Pirat[1]*

[1] ICGM, Univ Montpellier, ENSCM, CNRS, Montpellier, France
[2] Université des Sciences et Techniques de Masuku, Franceville, Gabon
[3] Department of Chemistry, K. N. Toosi University of Technology, Tehran, Iran
[4] Université Le Havre Normandie, Normandie Univ, URCOM UR 3221, FR CNRS 3038, Le Havre, France
[5] Polymers Composites and Hybrids (PCH), IMT Mines Ales, 30319 Ales, France
[6] University Abdou Moumouni of Niamey, Niamey, Niger
* Correspondence: jean-luc.pirat@enscm.fr (J.-L.P.); Tel.: (+033. 448 792 014); tahar.ayad@enscm.fr (T.A.); Tel.: (+033. 448 792 015)

Table of Contents

Comments on the residue contents in MCC versus in TGA....2
$^{31}P\{^1H\}$, ^1H and $^{13}C\{^1H\}$ NMR and HRMS spectra of the synthesized compounds 3
S1 : (*E*)-2-Phenyl-3-styryl-1*H*-isophosphinoline 2-oxide (**3a**) 3
S2: (*Z*)-3-(1,2-Diphenylvinyl)-2-phenyl-1*H*-isophosphinoline 2-oxide (**3b**) 5
S3: 3-(Cyclohex-2-en-1-yl)-2-phenyl-1*H*-isophosphinoline 2-oxide (**3c**) 9
S4: Dimethyl (*E*)-(2-(2-oxido-2-phenyl-1*H*-isophosphinolin-3-yl)vinyl)phosphonate (**3d**).... 11
S5: Diethyl (*E*)-(2-(2-oxido-2-phenyl-1*H*-isophosphinolin-3-yl)vinyl)phosphonate (**3e**) 14
S6: Methyl (*E*)-3-(2-oxido-2-phenyl-1*H*-isophosphinolin-3-yl)acrylate (**3f**) 16
S7: (*E*)-*N,N*-Dimethyl-3-(2-oxido-2-phenyl-1*H*-isophosphinolin-3-yl)acrylamide (**3g**) 19
S8: (*E*)-3-(2-Oxido-2-phenyl-1*H*-isophosphinolin-3-yl)acrylonitrile (**3h**) 21
S9: Methyl 2-methyl-3-(2-oxido-2-phenyl-1*H*-isophosphinolin-3-yl)acrylate (**3ia**).... 24
S10: Methyl 2-((2-oxido-2-phenyl-1*H*-isophosphinolin-3-yl)methyl)acrylate (**3ib**) 28
S11: Methyl 3-(2-oxido-2-phenyl-1*H*-isophosphinolin-3-yl)-2-((2-oxido-2-phenyl-1*H*-isophosphinolin-3-yl)methyl)acrylate (**3ic**).... 30
S12: Ethyl 2-((2-oxido-2-phenyl-1*H*-isophosphinolin-3-yl)methyl)acrylate (**3ja**) 33
S13: Ethyl 2-((2-oxido-2-phenyl-1*H*-isophosphinolin-3-yl)methyl)acrylate (**3jb**).... 36
S14: Ethyl 3-(2-oxido-2-phenyl-1*H*-isophosphinolin-3-yl)-2-((2-oxido-2-phenyl-1*H*-isophosphinolin-3-yl)methyl)acrylate (**3jc**) 38
S15: (*E*)-*N*-(4-Methoxybenzyl)-3-(2-oxido-2-phenyl-1H-isophosphinolin-3-yl)acrylamide (**3k**).... 41
S16: (*E*)-2-Phenyl-3-(2-(phenylsulfonyl)vinyl)-1*H*-isophosphinoline 2-oxide (**3l**).... 43
S17: (*E*)-2-(2-(2-Oxido-2-phenyl-1*H*-isophosphinolin-3-yl)vinyl)isoindoline-1,3-dione (**3m**).... 45
Crystallographic data of sulfonated product **3l**.... 45

Comments on the residue contents in MCC versus in TGA

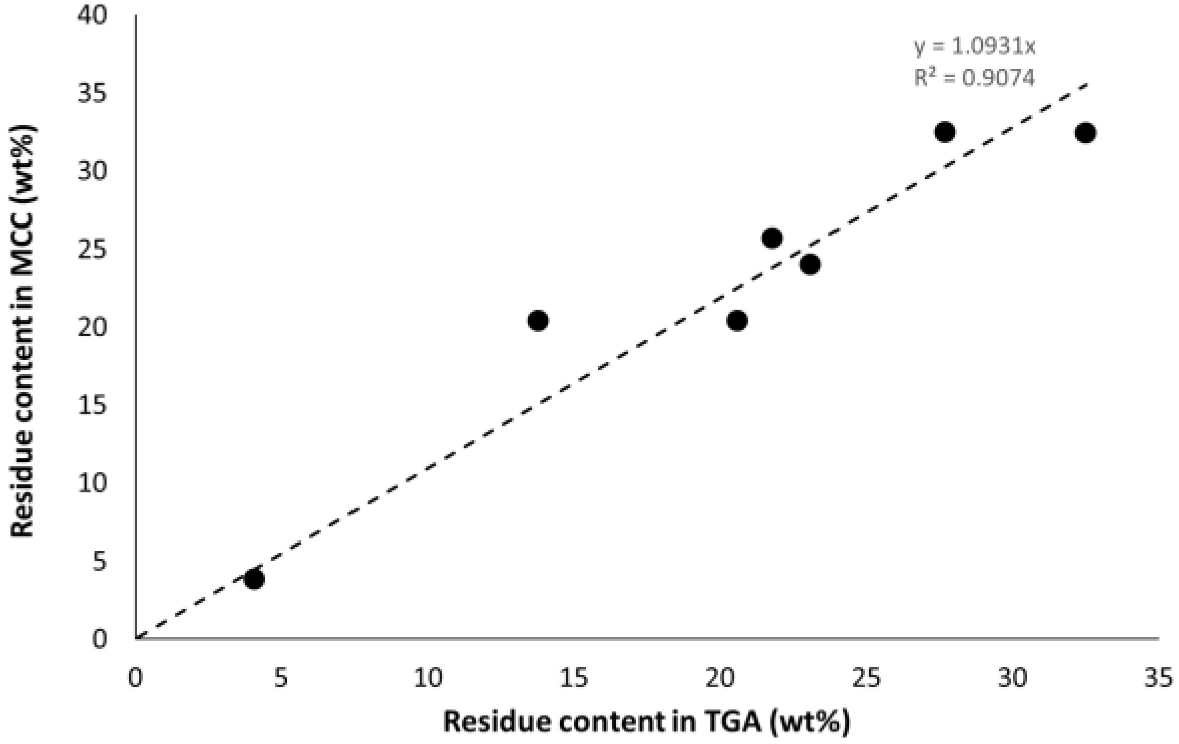

Figure SI1. Residue contents in MCC versus in TGA

There is a good agreement between the residue contents measured in MCC and in TGA (Figure SI1) despite some differences that may be assigned to the test protocol. The heating rate is much higher in MCC: in some rare cases, the heating rate can impact significantly the pyrolysis pathway and the final residue content.

$^{31}P\{^1H\}$, ^1H and $^{13}C\{^1H\}$ NMR and HRMS spectra of the synthesized compounds

S1 : (*E*)-2-Phenyl-3-styryl-1*H*-isophosphinoline 2-oxide **(3a)**

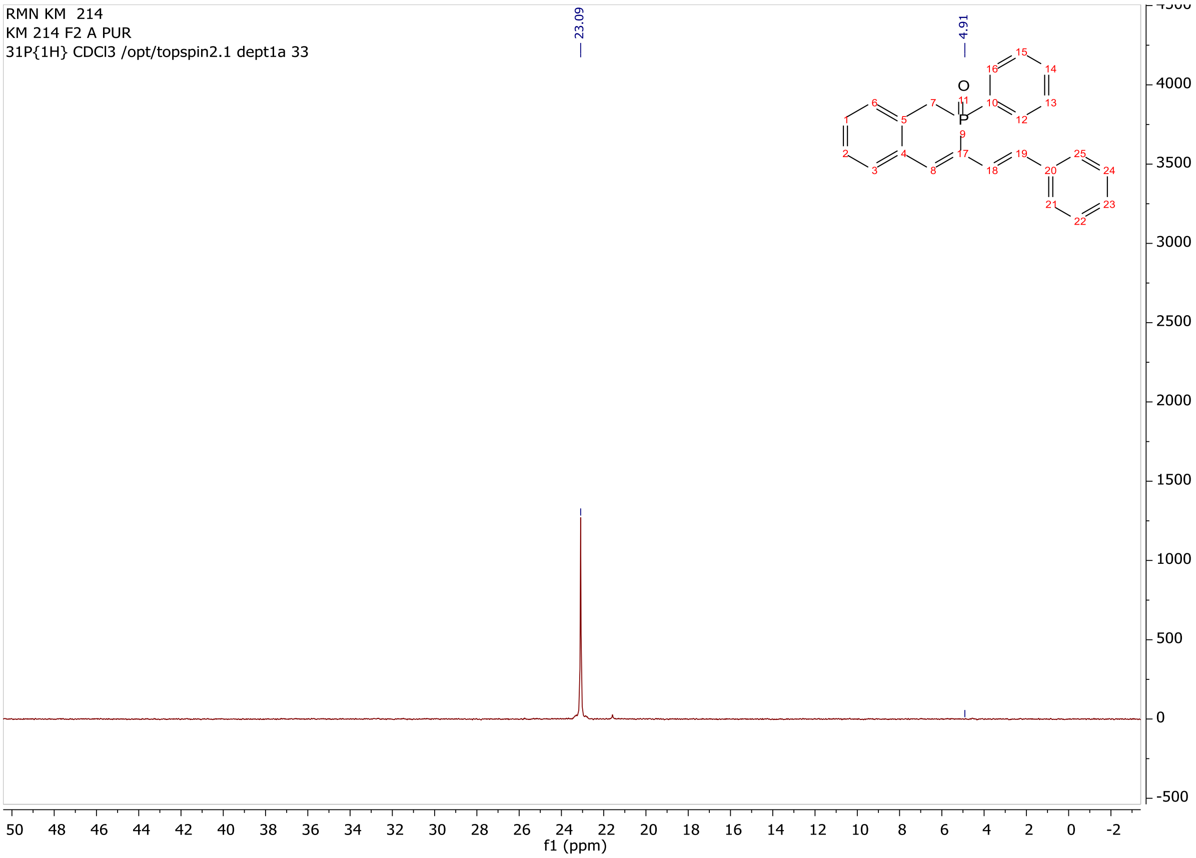

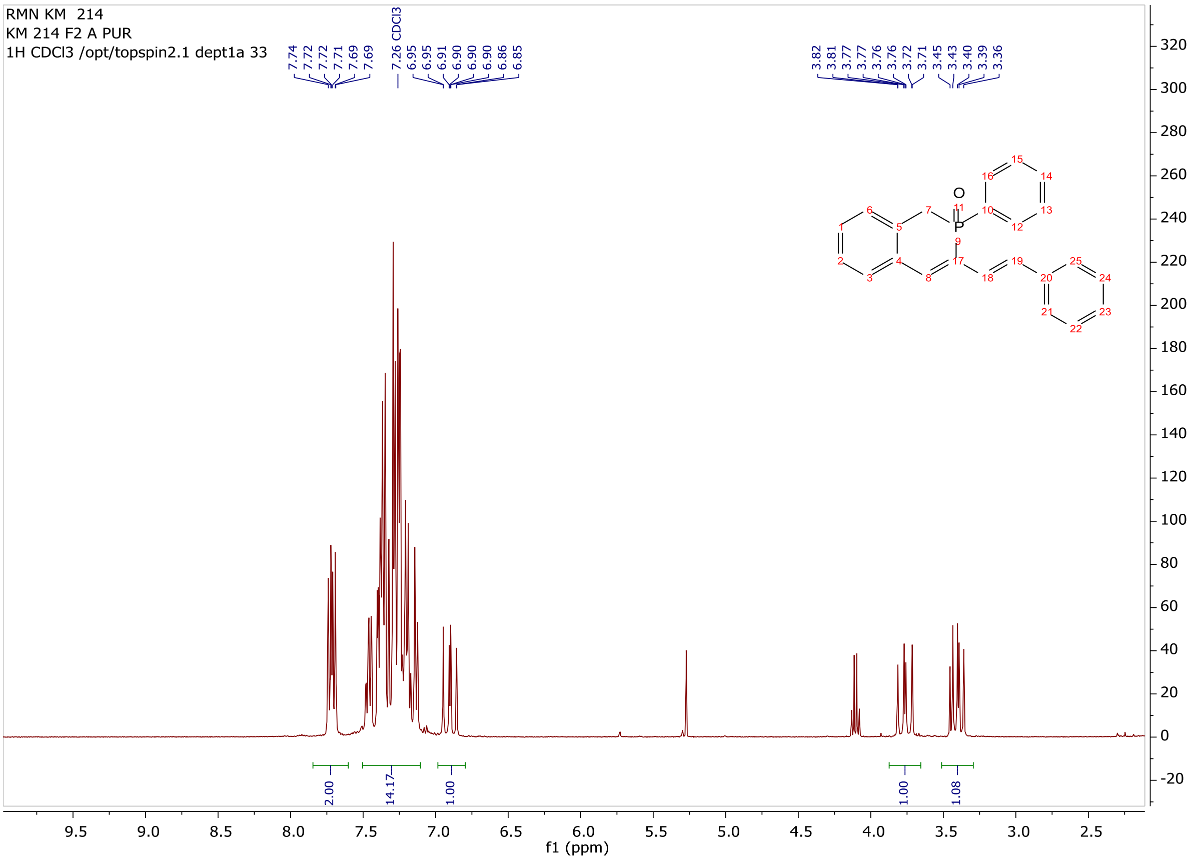

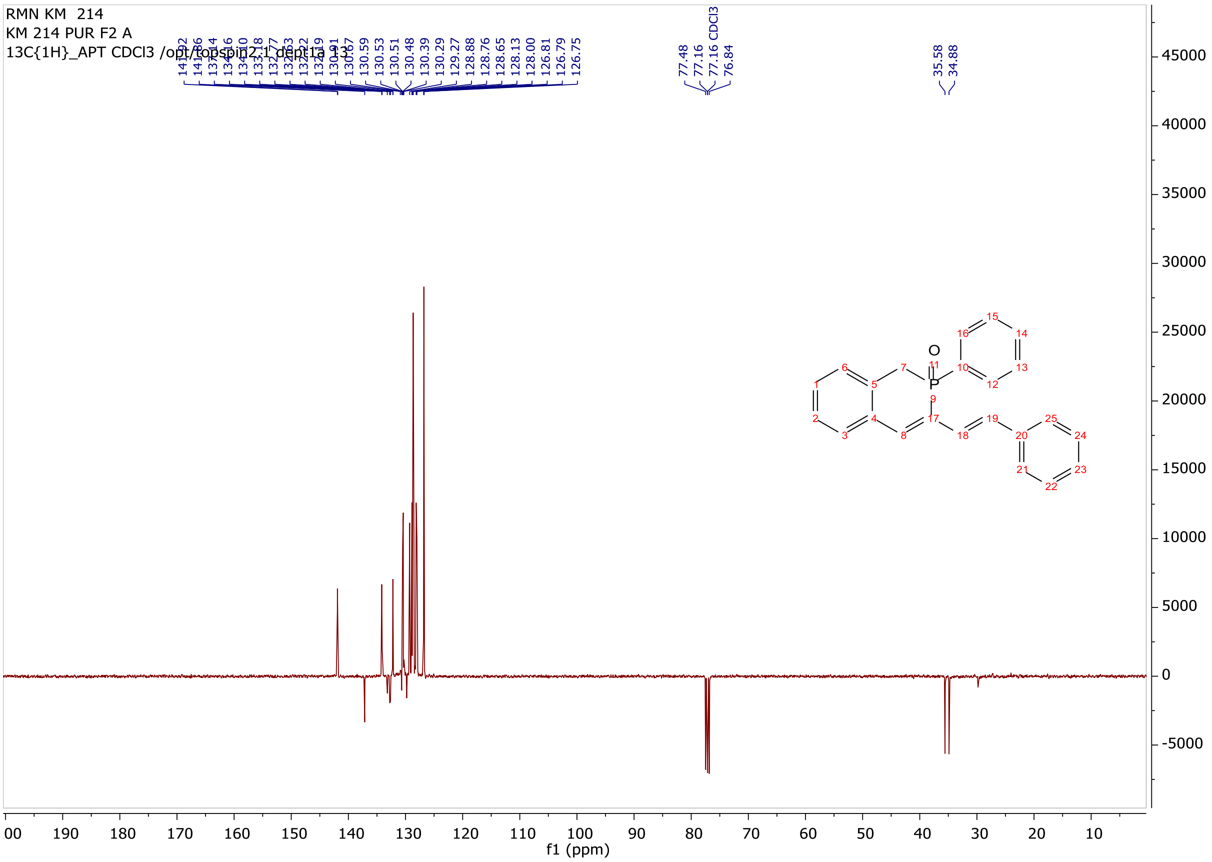

D:\LMP\OLAF\2023\data\S2328\MMN-4h_1.raw 7/17/2023 4:25:23 PM

Code OLAF MMN-4h_1.raw **Echantillon** KM214 **Instrument** Orbitrap ID-X

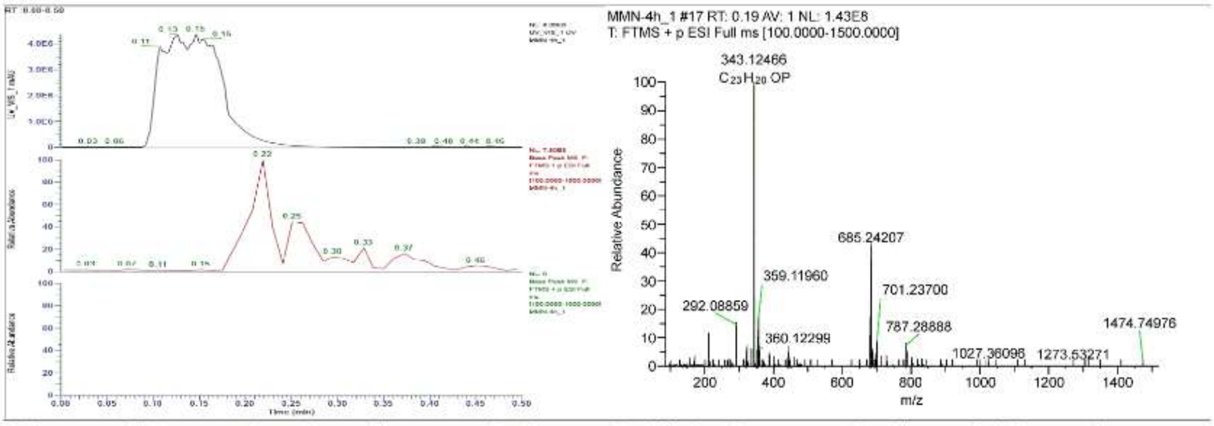

Display	File Name	Detector Type	Filter	Trace Type	Mass Defect Ran...	Ranges	Mass Tolerance	Delay Time	Comment
True	D:\LMP\OLAF \2023\data \S2328 \MMN-4h_1.raw	UV		UV_VIS_1	MDF Ranges		5	0	
True	D:\LMP\OLAF \2023\data \S2328 \MMN-4h_1.raw	MS	FTMS + p ESI Full ms [100.0000-1500.0 000]	Base Peak	MDF Ranges		5	0	
[illegible]	D:\LMP\OLAF \2023\data	[illegible]	FTMS + p ESI Full ms	[illegible]	[illegible]		[illegible]	[illegible]	

Rank	Peak Mass	Display Formula	Delta [ppm]	Theo. mass	Pattern Cov. [%]	# Matched Iso.	Combined Score	MS Cov. [%]	MSMS Matched...
1	343.12466	$C_{23}H_{20}OP$	0.10	343.12463	99.64	3	95.38	98.69	(Collection)
2	343.12466	$C_{12}H_{13}O_2N_{11}$	-0.45	343.12482	84.19	2	73.76	77.43	(Collection)
3	343.12466	$C_{13}H_{19}O_7N_4$	-0.47	343.12483	84.27	2	73.65	77.43	(Collection)

S2: (*Z*)-3-(1,2-Diphenylvinyl)-2-phenyl-1*H*-isophosphinoline 2-oxide **(3b)**

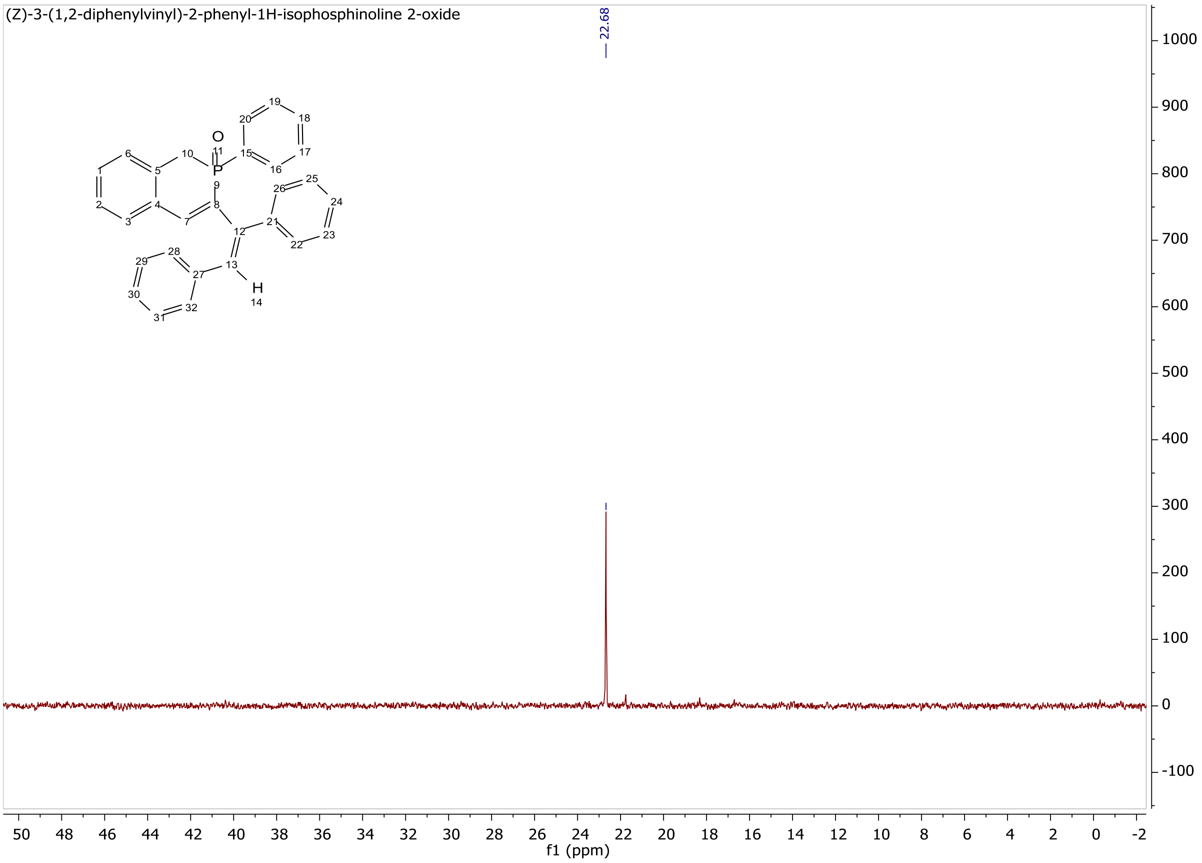

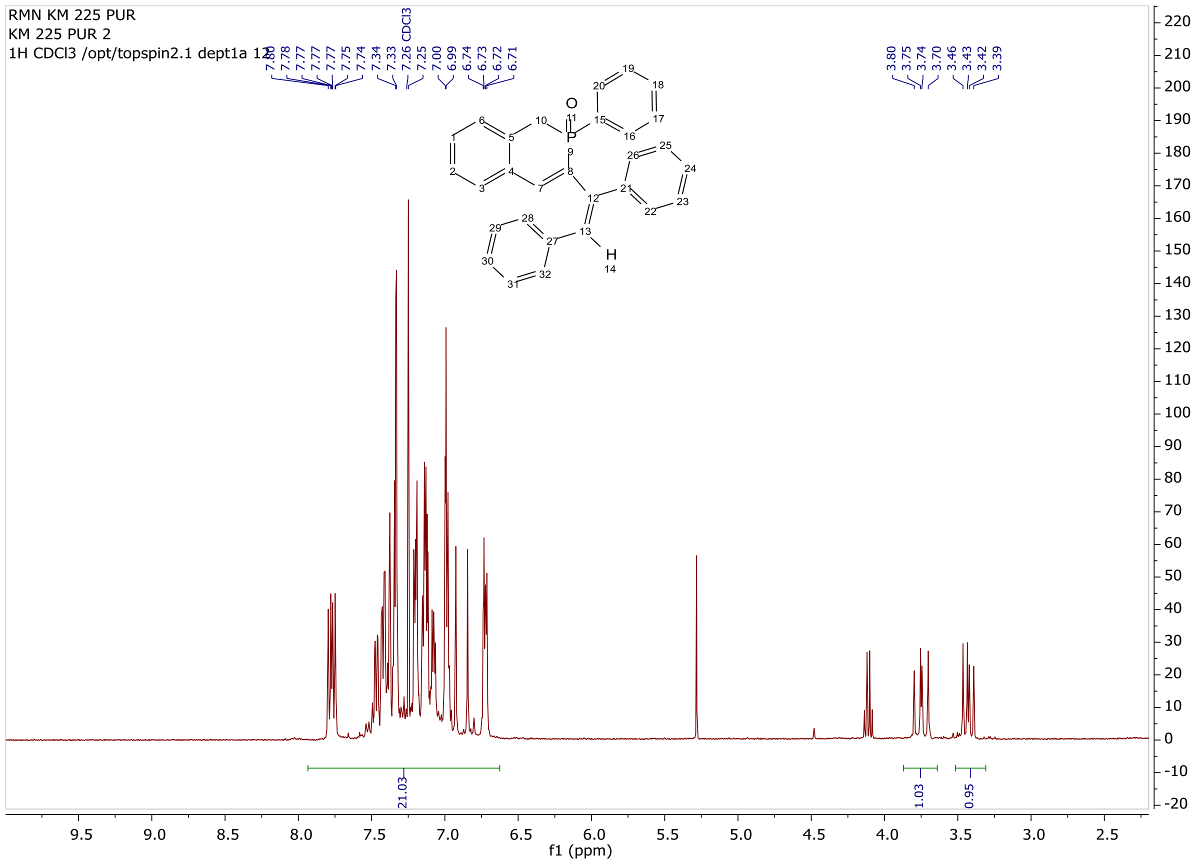
RMN KM 225 PUR
KM 225 PUR 2
1H CDCl3 /opt/topspin2.1 dept1a 12
f1 (ppm)

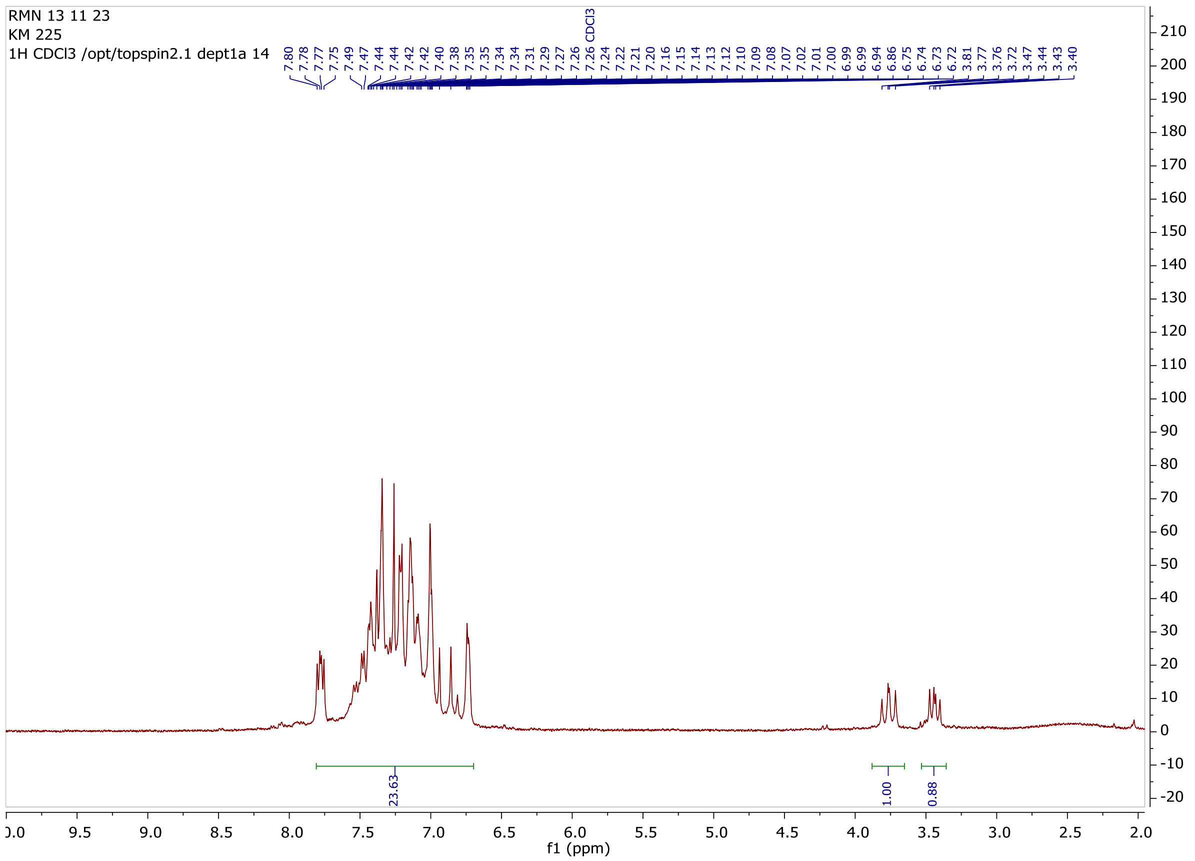
RMN 13 11 23
KM 225
1H CDCl3 /opt/topspin2.1 dept1a 14
f1 (ppm)

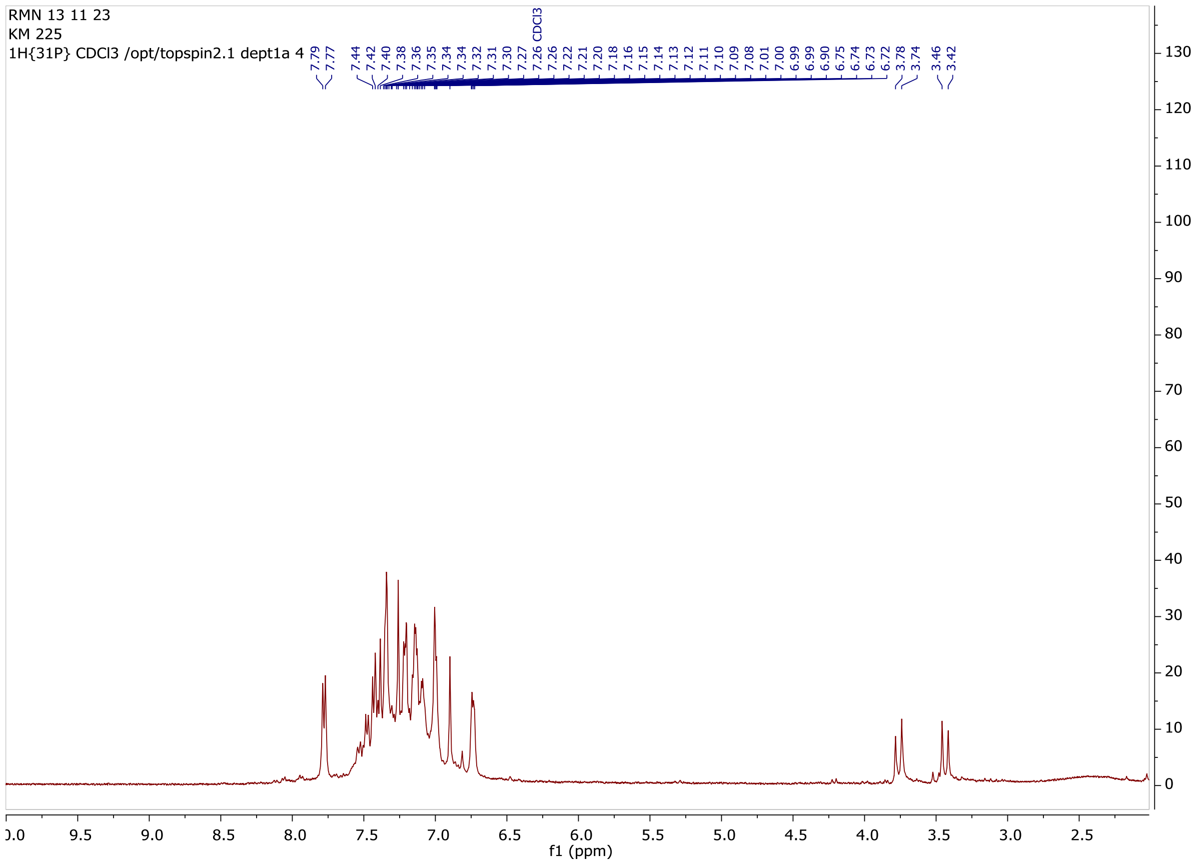
RMN 13 11 23
KM 225
1H{31P} CDCl3 /opt/topspin2.1 dept1a 4
f1 (ppm)

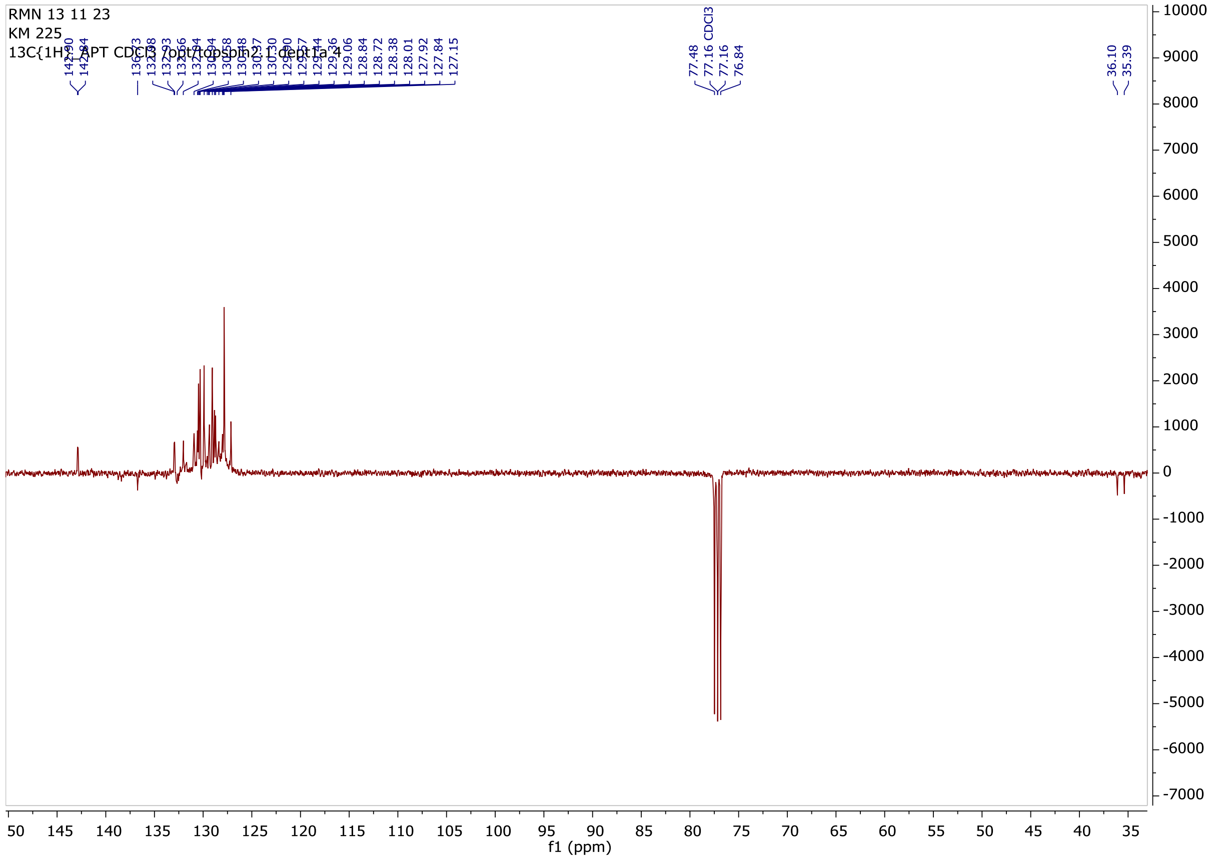
RMN 13 11 23
KM 225
f1 (ppm)

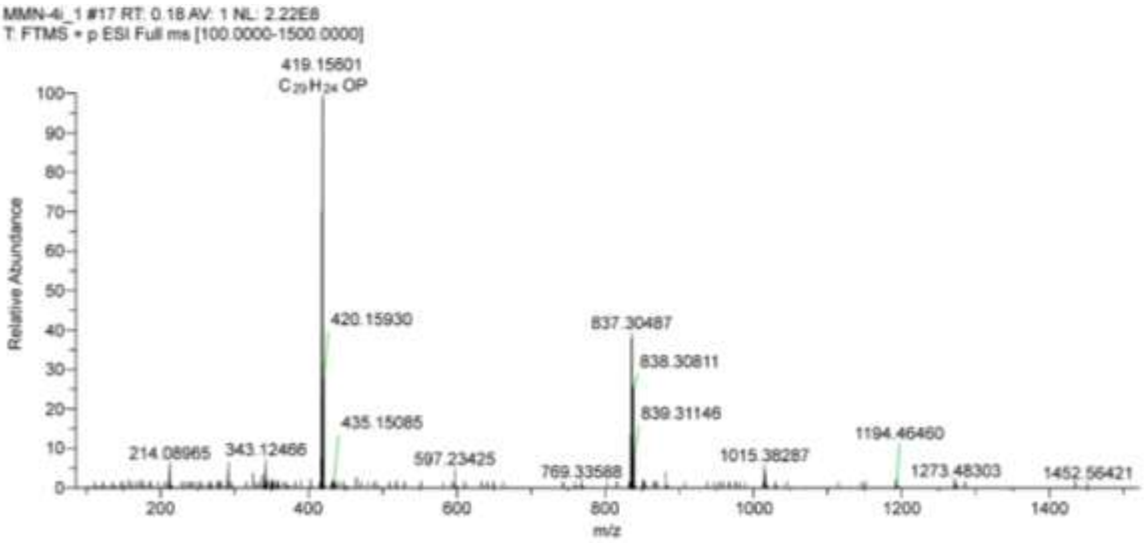

Rank	Peak Mass	Display Formula	Delta [ppm]	Theo. mass	Pattern Cov. [%]	# Matched Iso.	Combined Score	MS Cov. [%]	MSMS Matched...	N
1	419.15601	$C_{29}H_{24}OP$	0.18	419.15593	99.66	4	96.81	99.83	(Collection)	
2	419.15601	C[illegible]H[illegible]O[illegible]N[illegible]	-0.27	419.15612	79.18	4	71.81	75.33	(Collection)	[illegible]
3	419.15601	C[illegible]H[illegible]O[illegible]N[illegible]	-0.29	419.15613	79.12	3	71.74	75.33	(Collection)	

S3: 3-(Cyclohex-2-en-1-yl)-2-phenyl-1*H*-isophosphinoline 2-oxide **(3c)**

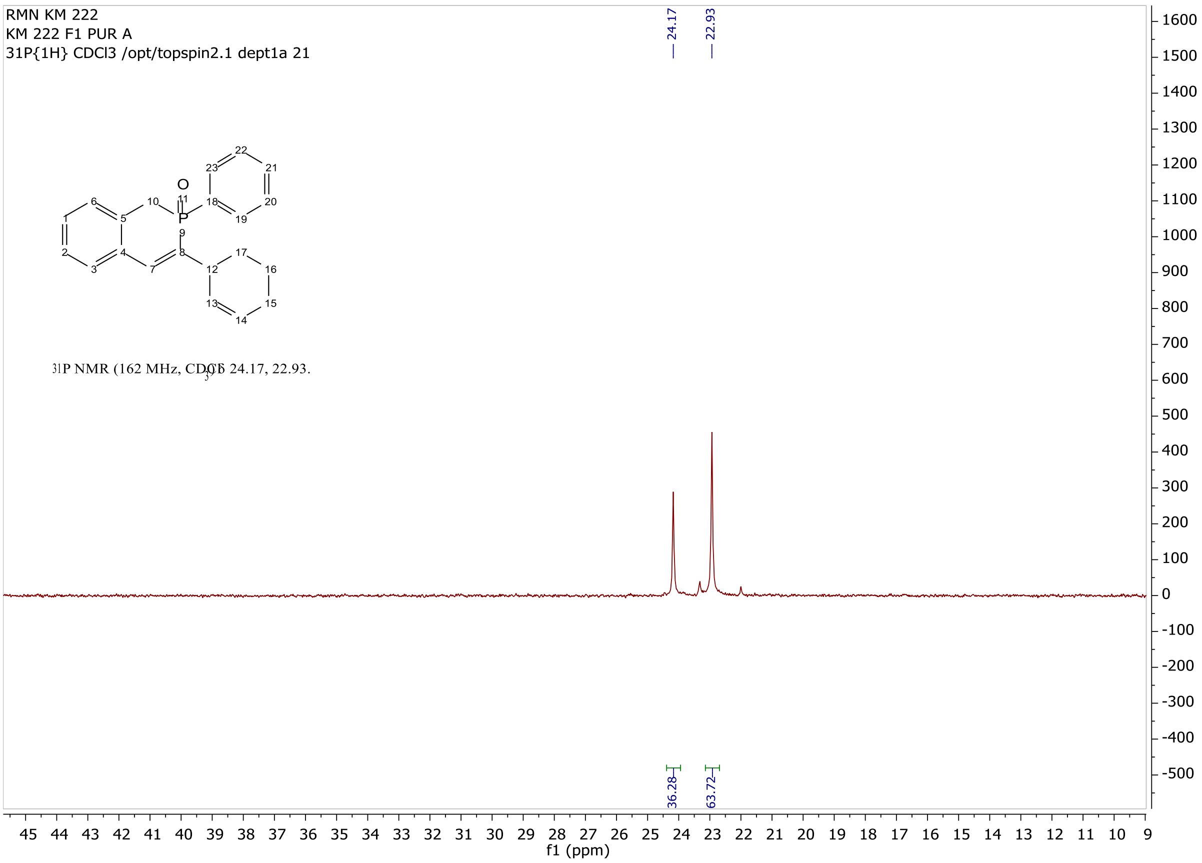

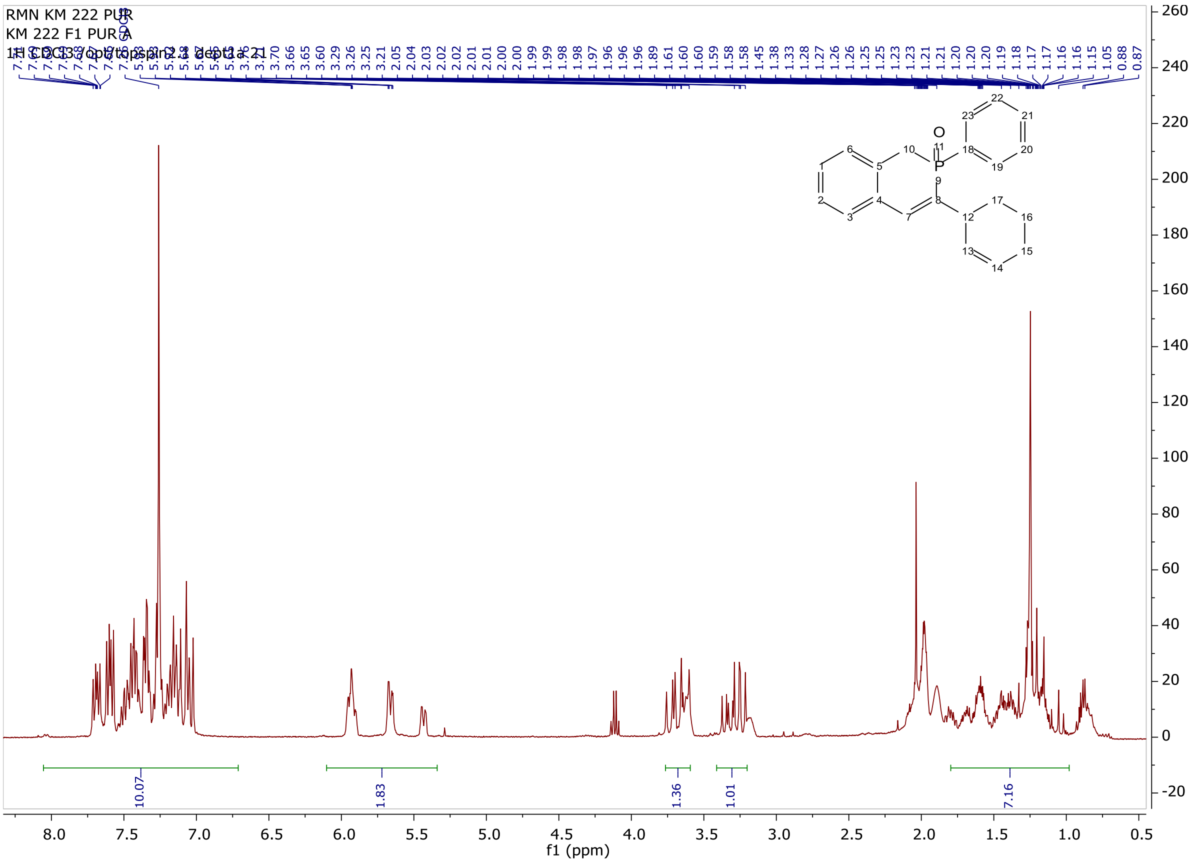

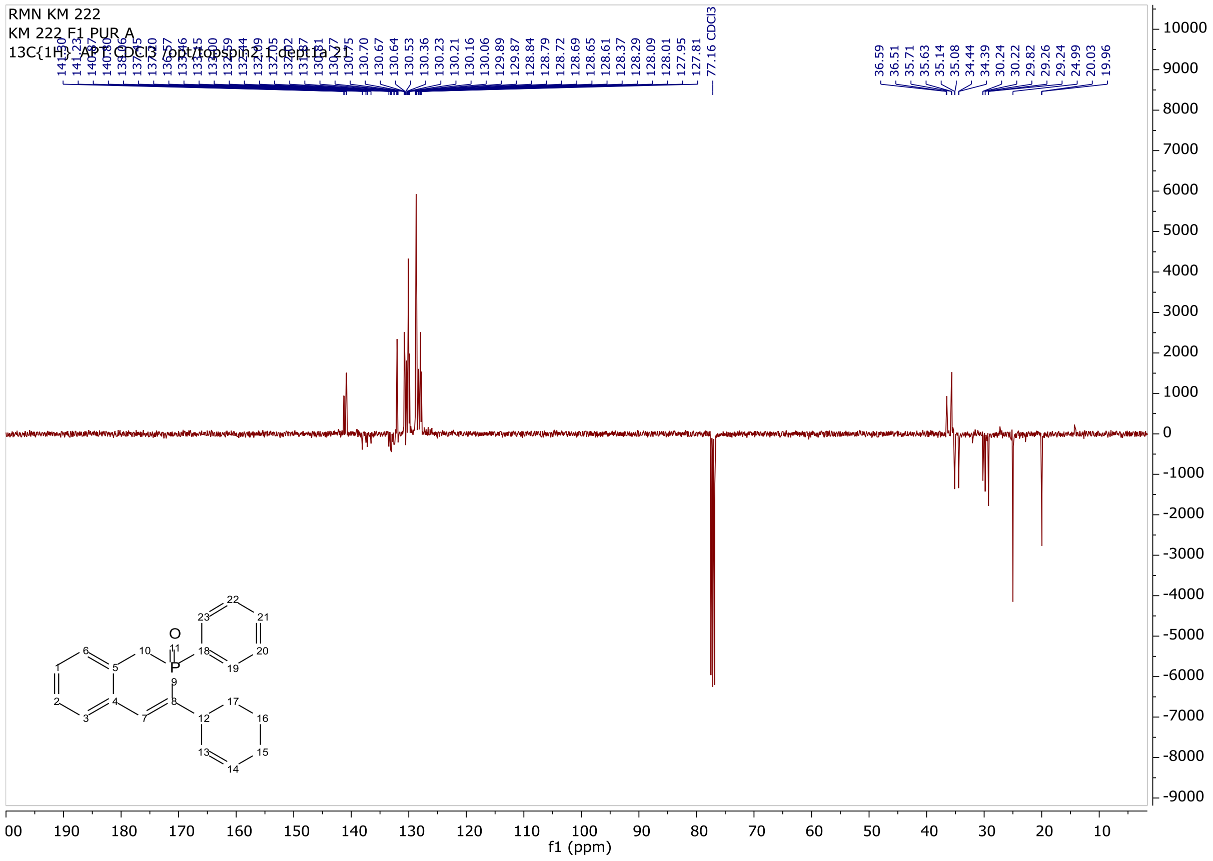

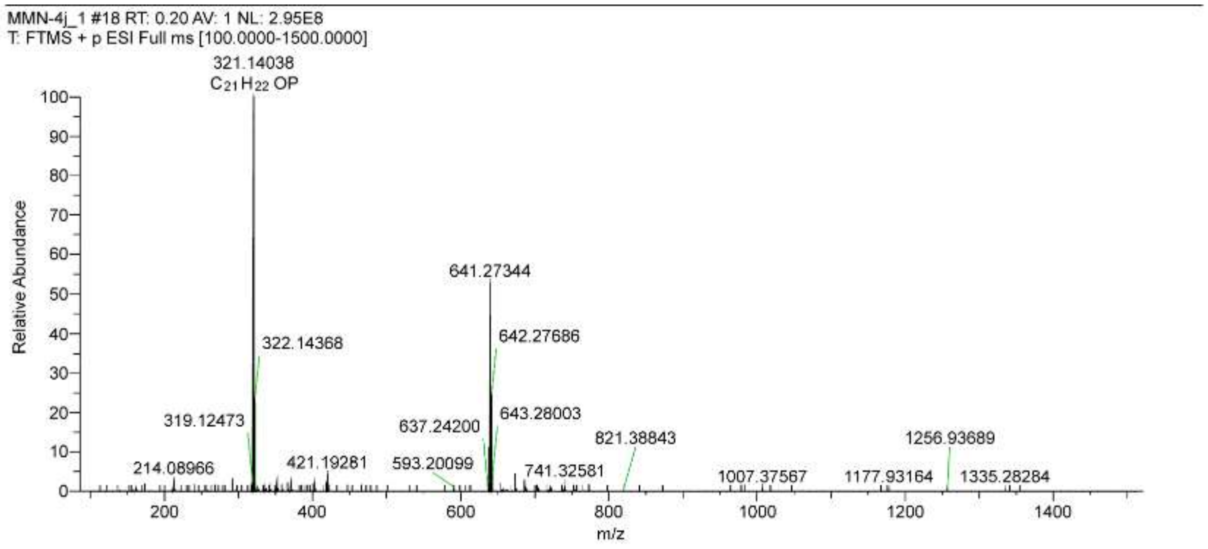

Rank	Peak Mass	Display Formula	Delta [mmu]	Theo. mass	Pattern Cov. [%]	# Matched Iso.	Combined Score	MS Cov. [%]	MSMS Matched...
1	321.14038	$C_{21}H_{22}OP$	0.10	321.14028	99.63	4	96.76	99.92	(Collection)
2	321.14038	$C_{10}H_{15}O_{3}N_{11}$	-0.09	321.14047	85.87	2	75.87	79.67	(Collection)
3	321.14038	$C_{6}H_{18}O_{2}N_{12}P$	-0.40	321.14078	89.26	1	75.84	79.67	(Collection)

S4: Dimethyl (*E*)-(2-(2-oxido-2-phenyl-1*H*-isophosphinolin-3-yl)vinyl)phosphonate **(3d)**

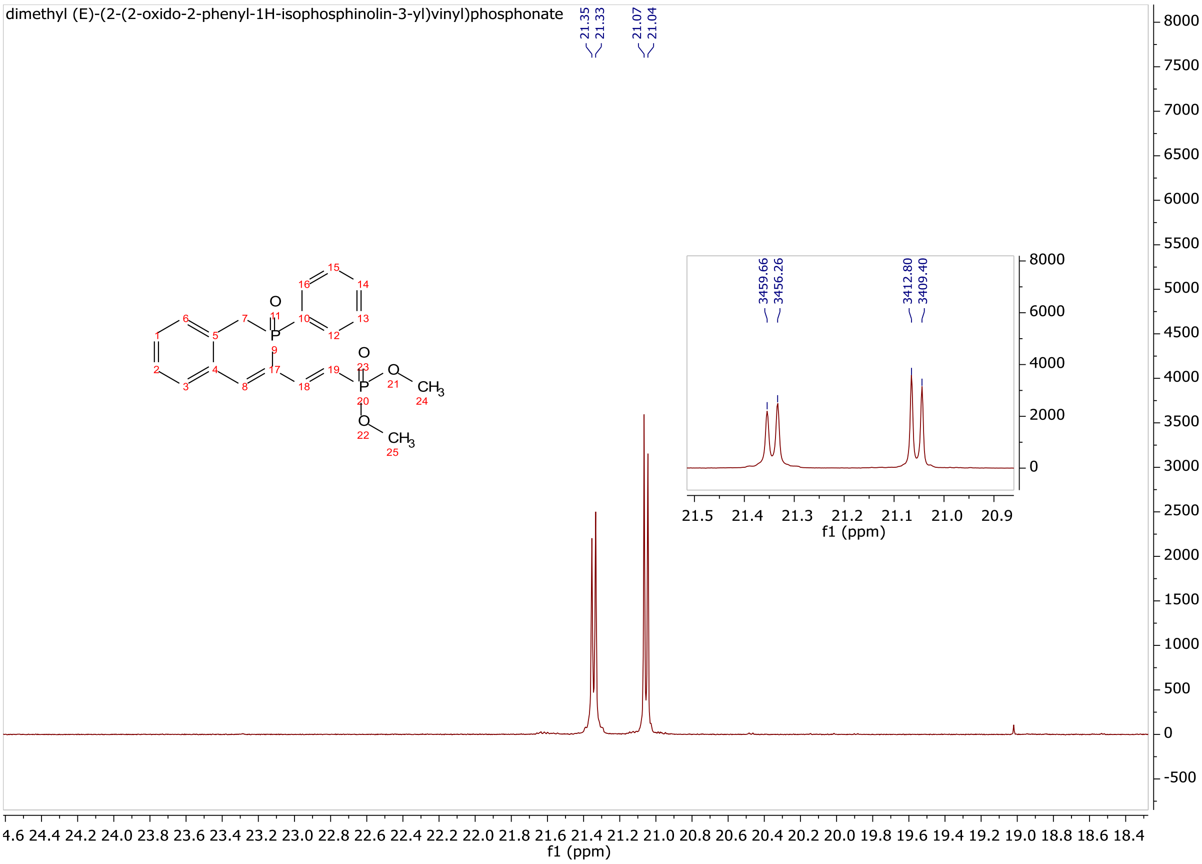

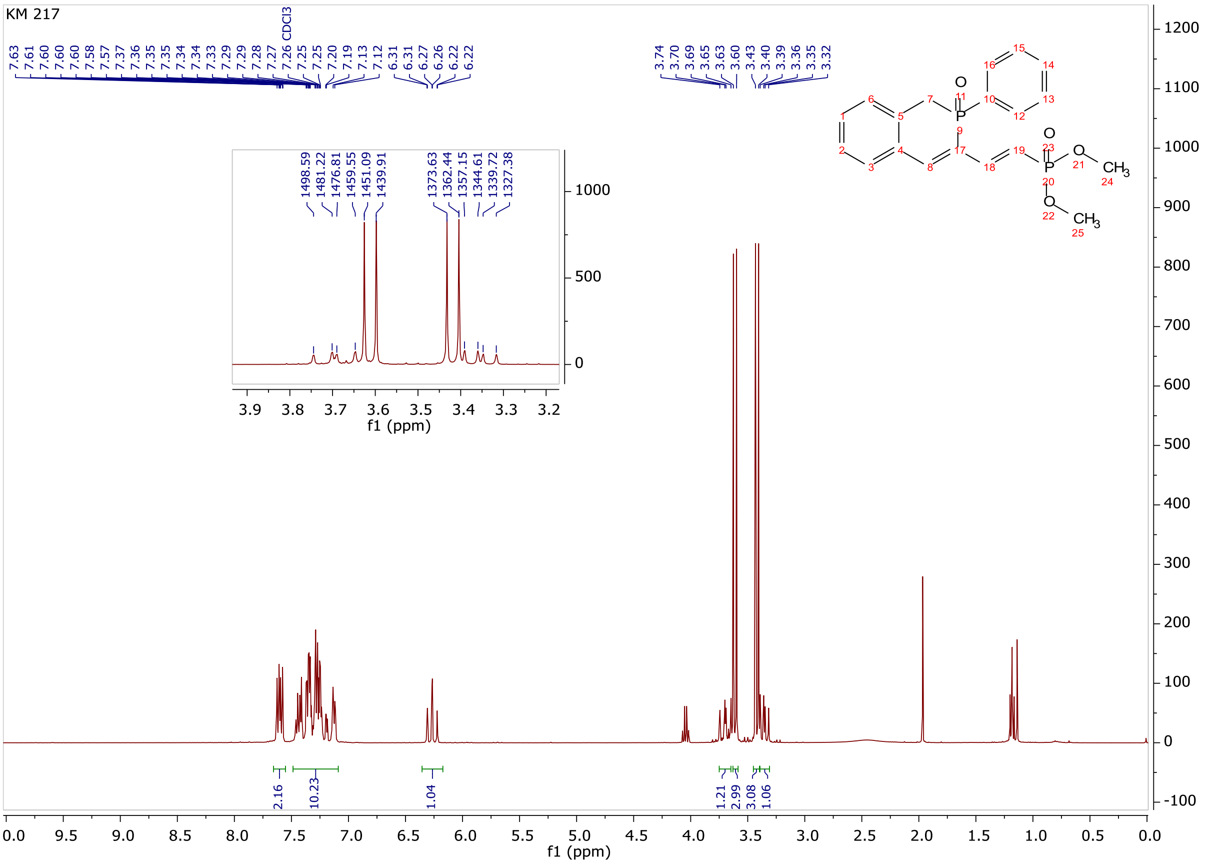

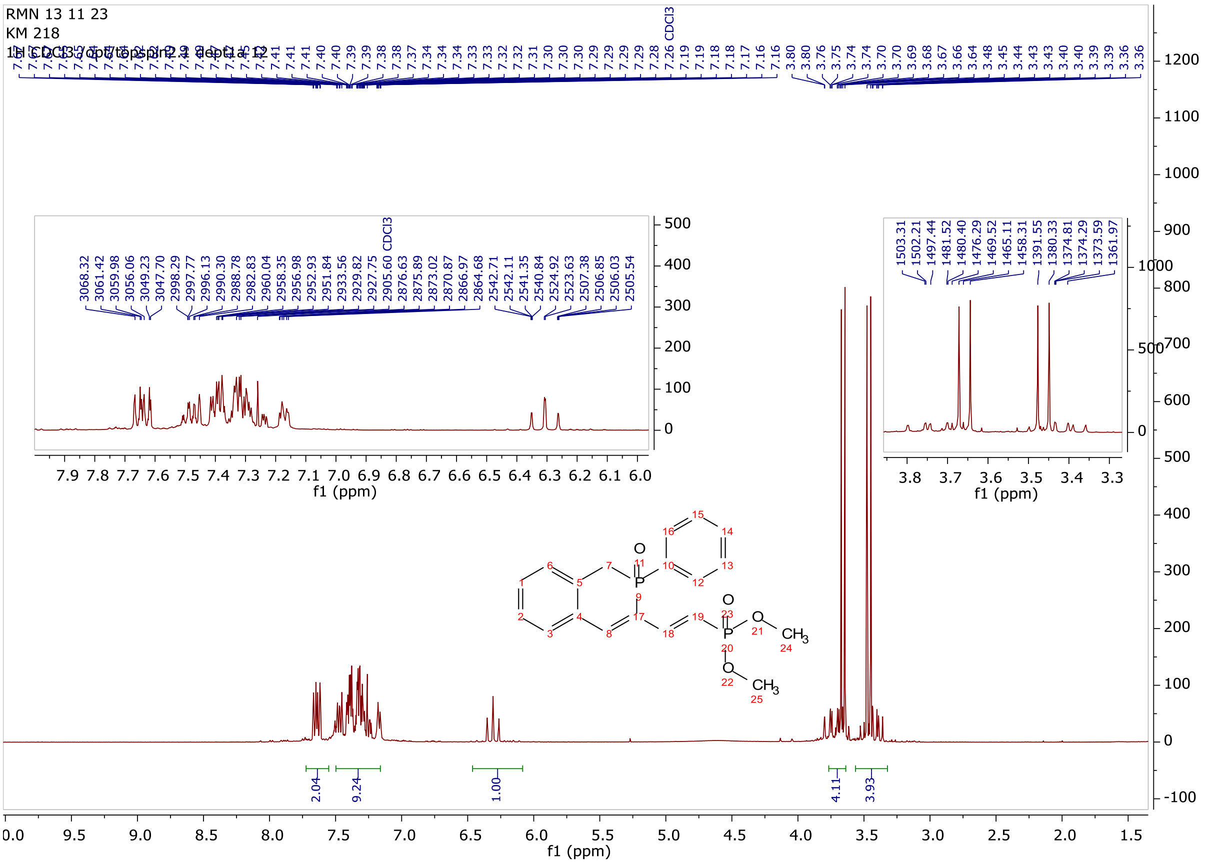

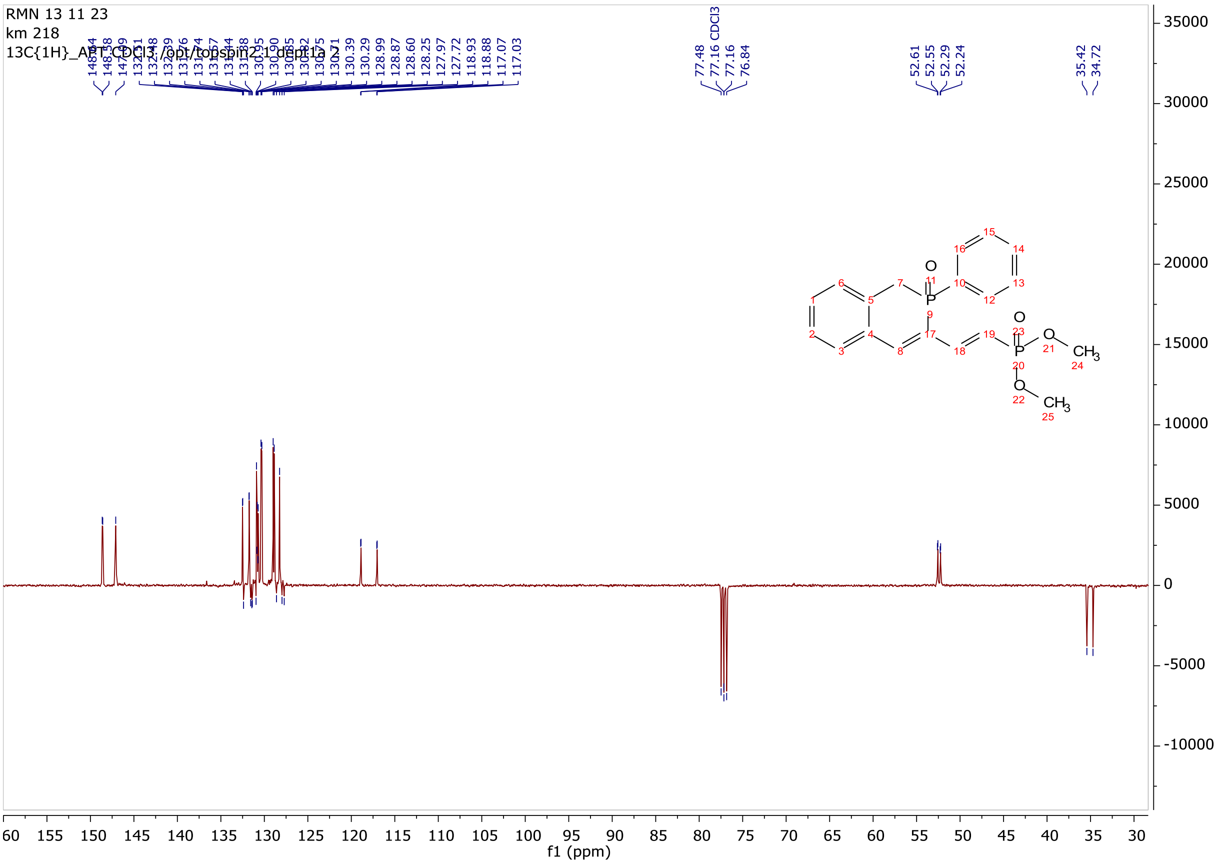

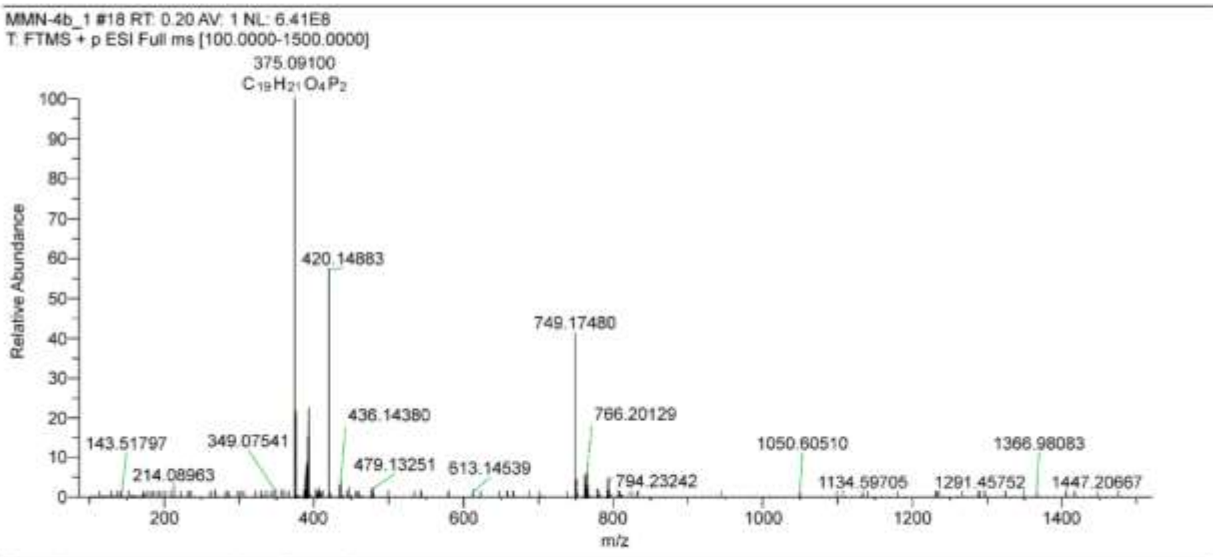

Rank	Peak Mass	Display Formula	Delta [ppm]	Theo. mass	Pattern Cov. [%]	# Matched Iso.	Combined Score	MS Cov. [%]	MSMS Matched...
1	375.09100	$C_{19}H_{21}O_4P_2$	0.12	375.09096	97.44	5	93.94	98.15	(Collection)
2	375.09100	[illegible]	-0.41	375.09116	87.67	4	77.98	81.62	(Collection)
3	375.09100	[illegible]	0.42	375.09085	84.38	5	77.96	81.62	(Collection)

S5: Diethyl (*E*)-(2-(2-oxido-2-phenyl-1*H*-isophosphinolin-3-yl)vinyl)phosphonate **(3e)**

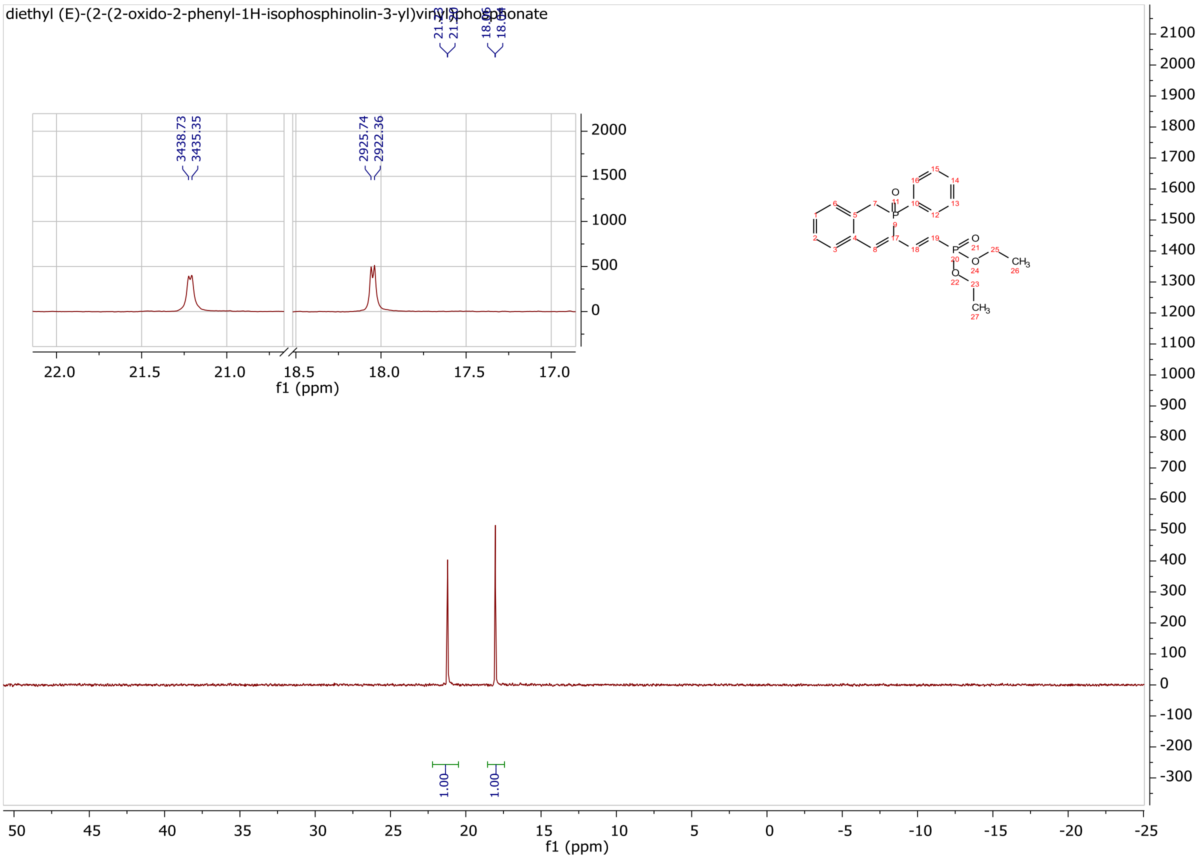

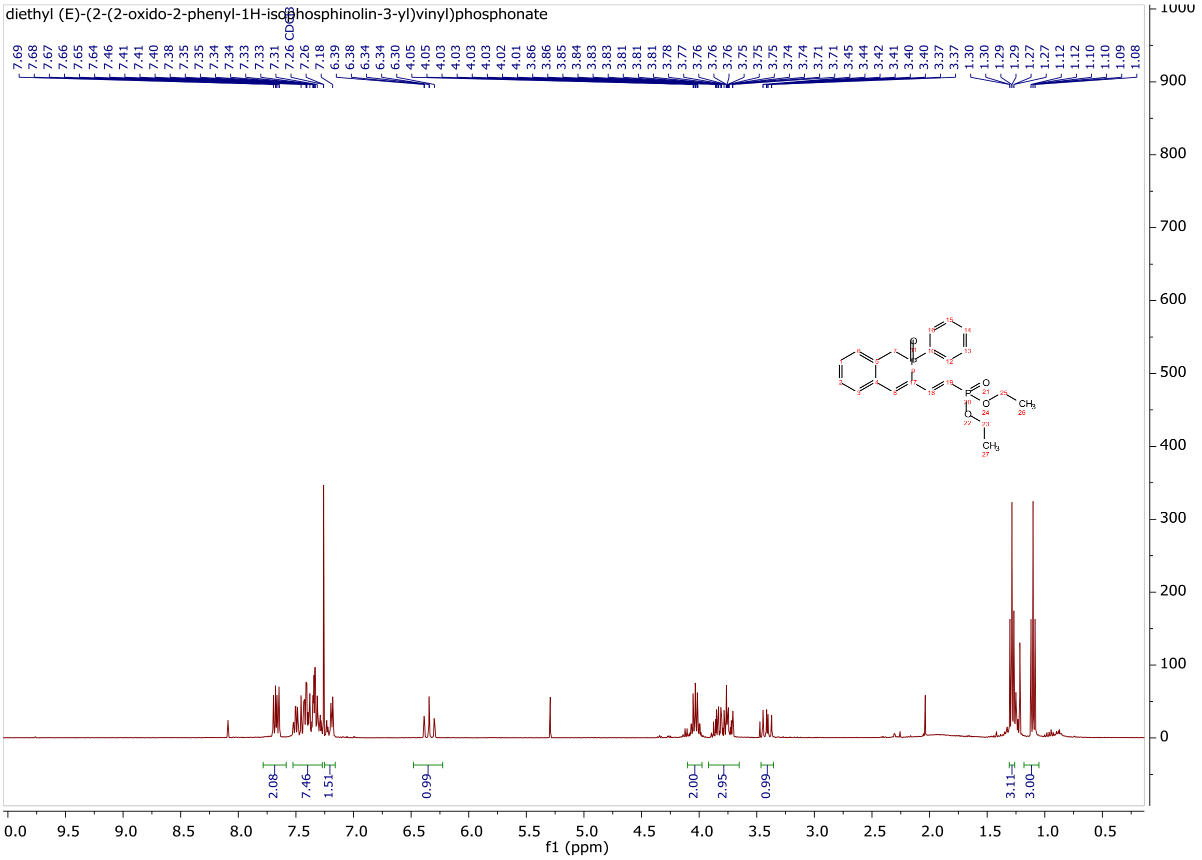

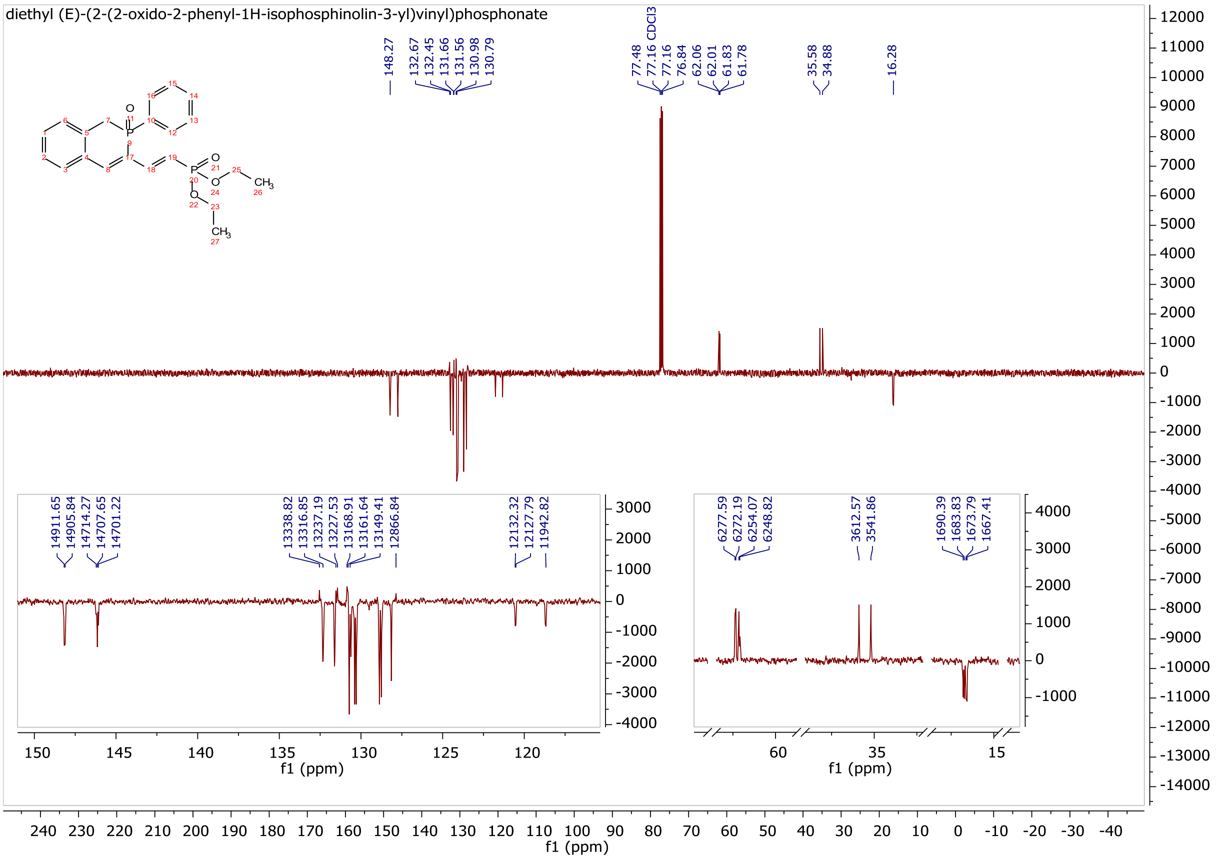

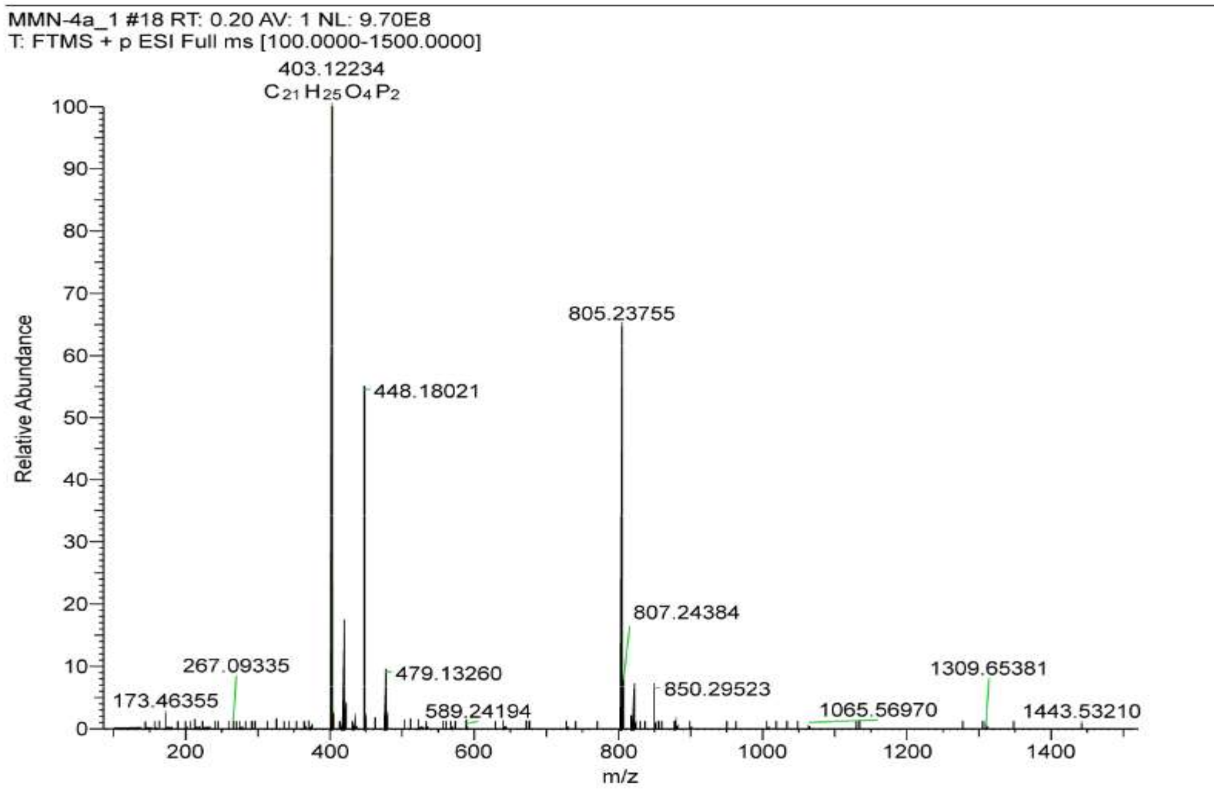

Rank	Peak Mass	Display For...	Delta [mmu]	Theo. mass	Pattern Cov....	# Matched I...	Combined S...	MS Cov. [%]	MSMS Matc...
1	403.12234	$C_{21}H_{25}O_4P_2$	0.09	403.12226	97.72	3	93.9	97.93	(Collection)
2	403.12234	$C_{15}H_{21}O_{10}N_3$	0.20	403.12215	95.86	4	93.46	97.93	(Collection)
3	403.12234	$C_6H_{21}O_5N_{12}P_2$	-0.42	403.12276	88.99	2	77.79	81.58	(Collection)

S6: Methyl (*E*)-3-(2-oxido-2-phenyl-1*H*-isophosphinolin-3-yl)acrylate **(3f)**

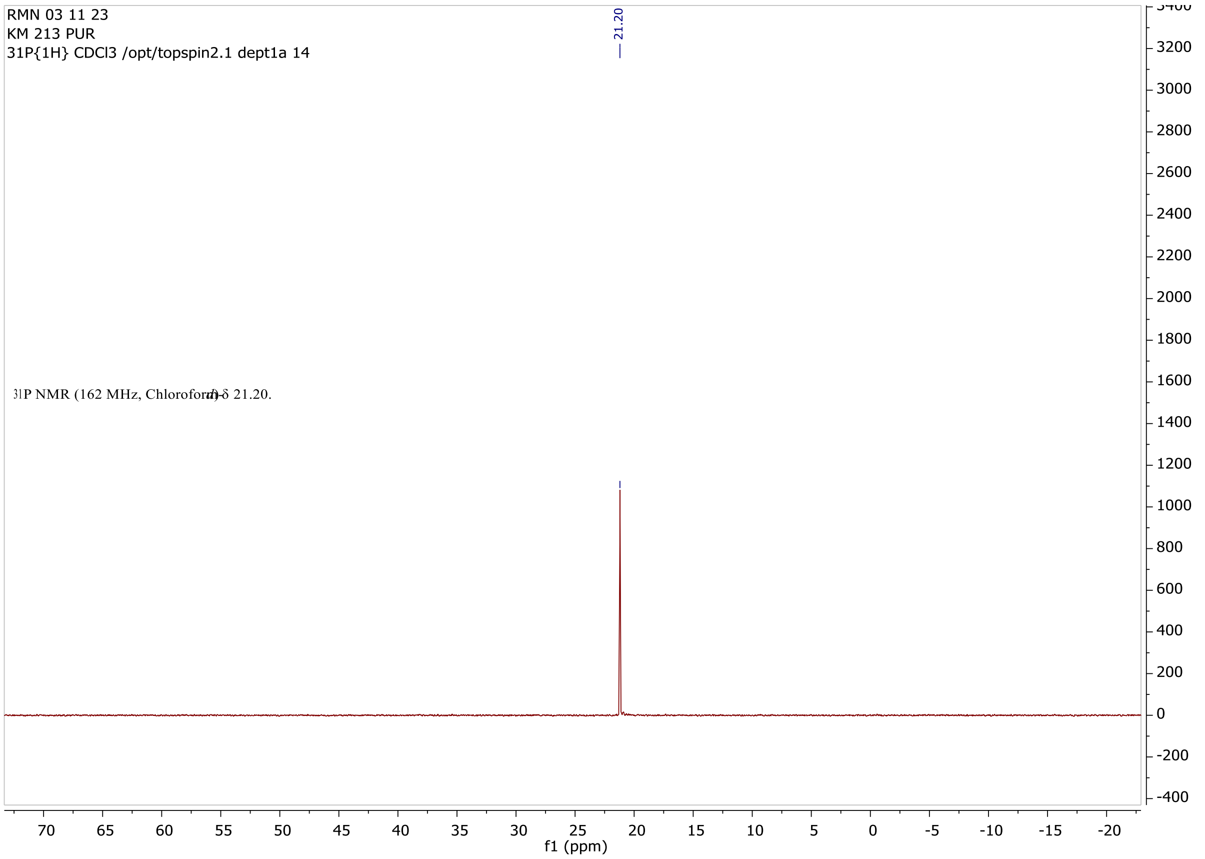

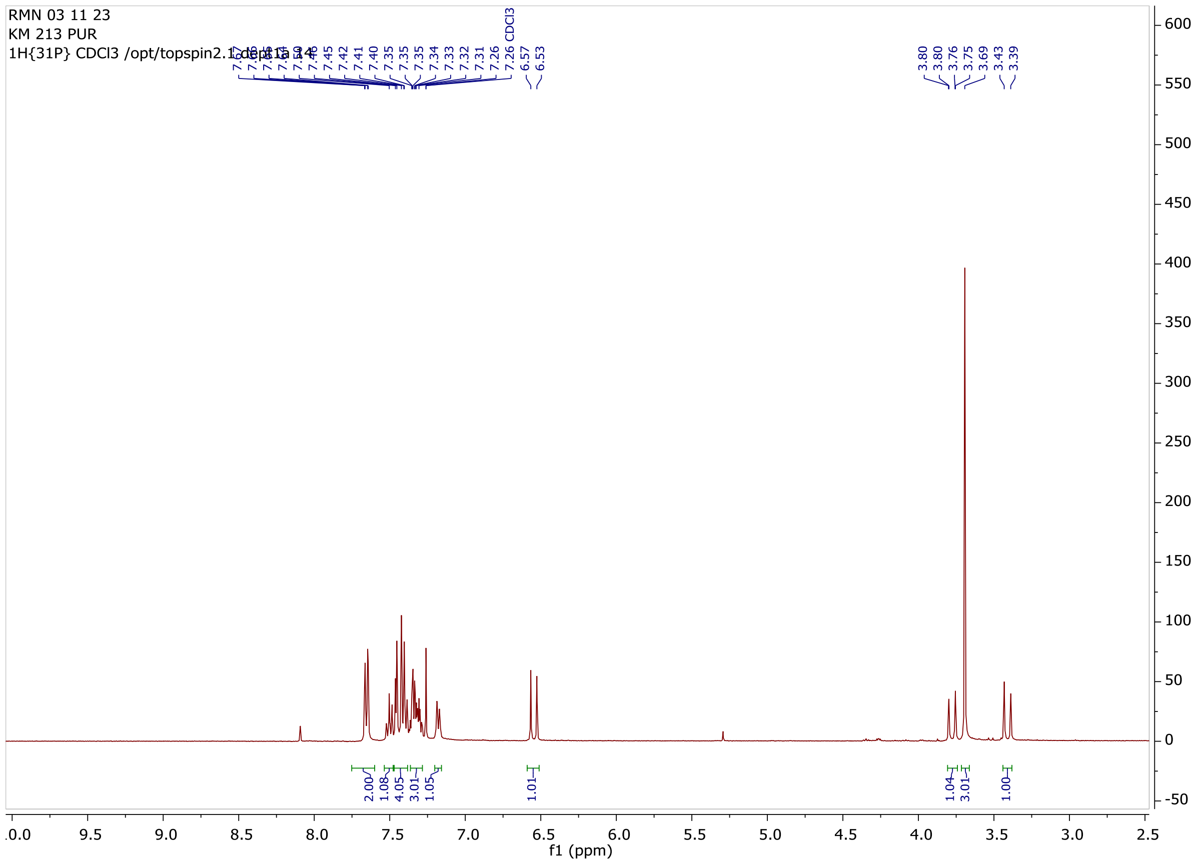
RMN 03 11 23
KM 213 PUR
1H{31P} CDCl3 /opt/topspin2.1 dept1a 14
f1 (ppm)

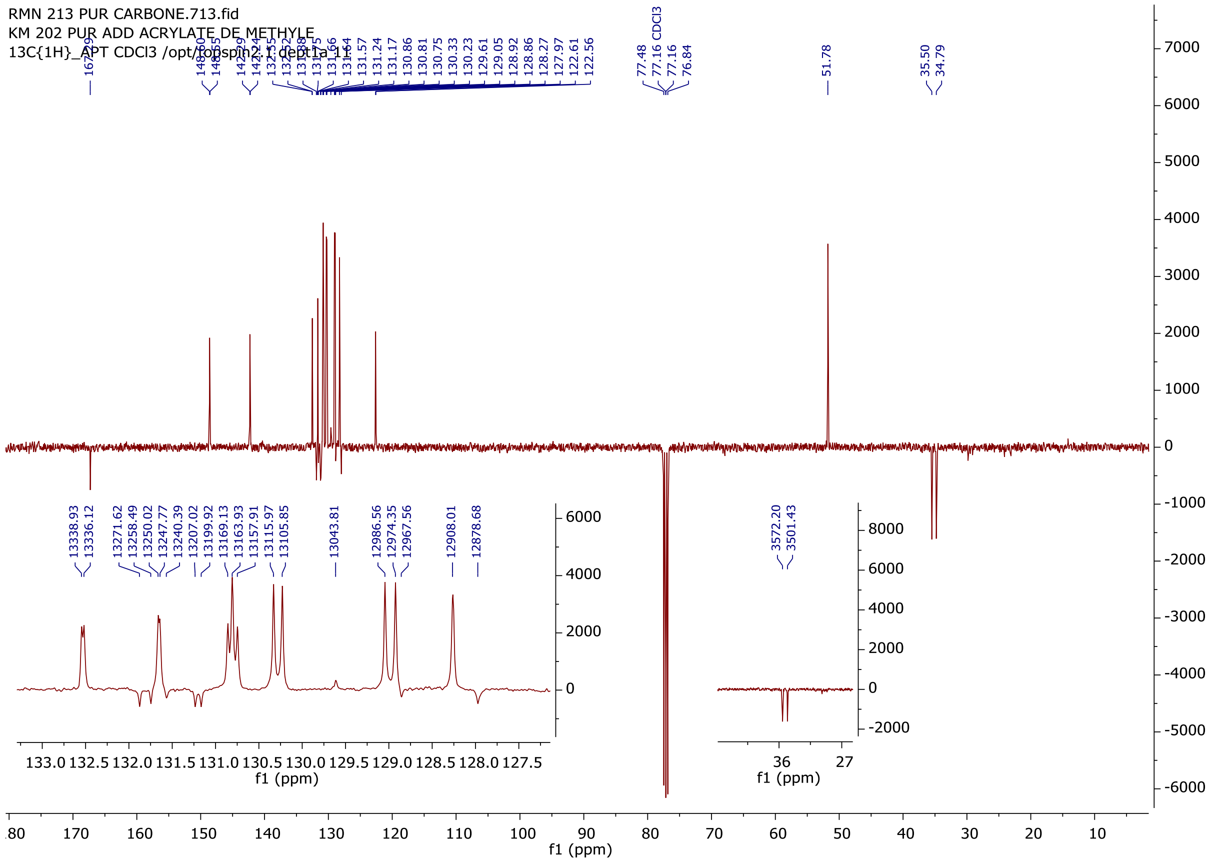
RMN 213 PUR CARBONE.713.fid
KM 202 PUR ADD ACRYLATE DE METHYLE
13C{1H}_APT CDCl3 /opt/topspin2.1 dept1a 11
f1 (ppm)

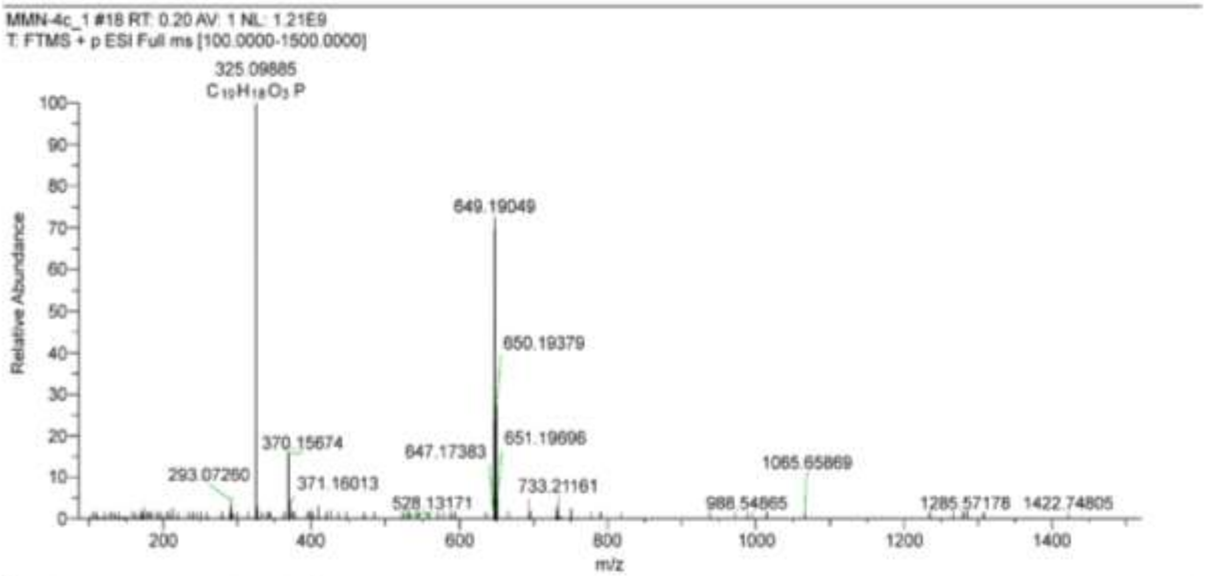

Rank	Peak Mass	Display Formula	Delta [ppm]	Theo. mass	Pattern Cov. [%]	# Matched Iso.	Combined Score	MS Cov. [%]	MSMS Matched...
1	325.09885	C[illegible]H[illegible]O[illegible]P	0.12	325.09881	99.13	5	95.6	99.58	(Collection)
2	325.09885	C[illegible]H[illegible]O[illegible]N[illegible]	-0.49	325.09900	87.75	4	77.85	81.52	(Collection)
3	325.09885	C[illegible]H[illegible]O[illegible]N[illegible]	-0.47	325.09900	87.4	2	77.44	81.38	(Collection)

S7: (*E*)-*N,N*-Dimethyl-3-(2-oxido-2-phenyl-1*H*-isophosphinolin-3-yl)acrylamide (**3g**)

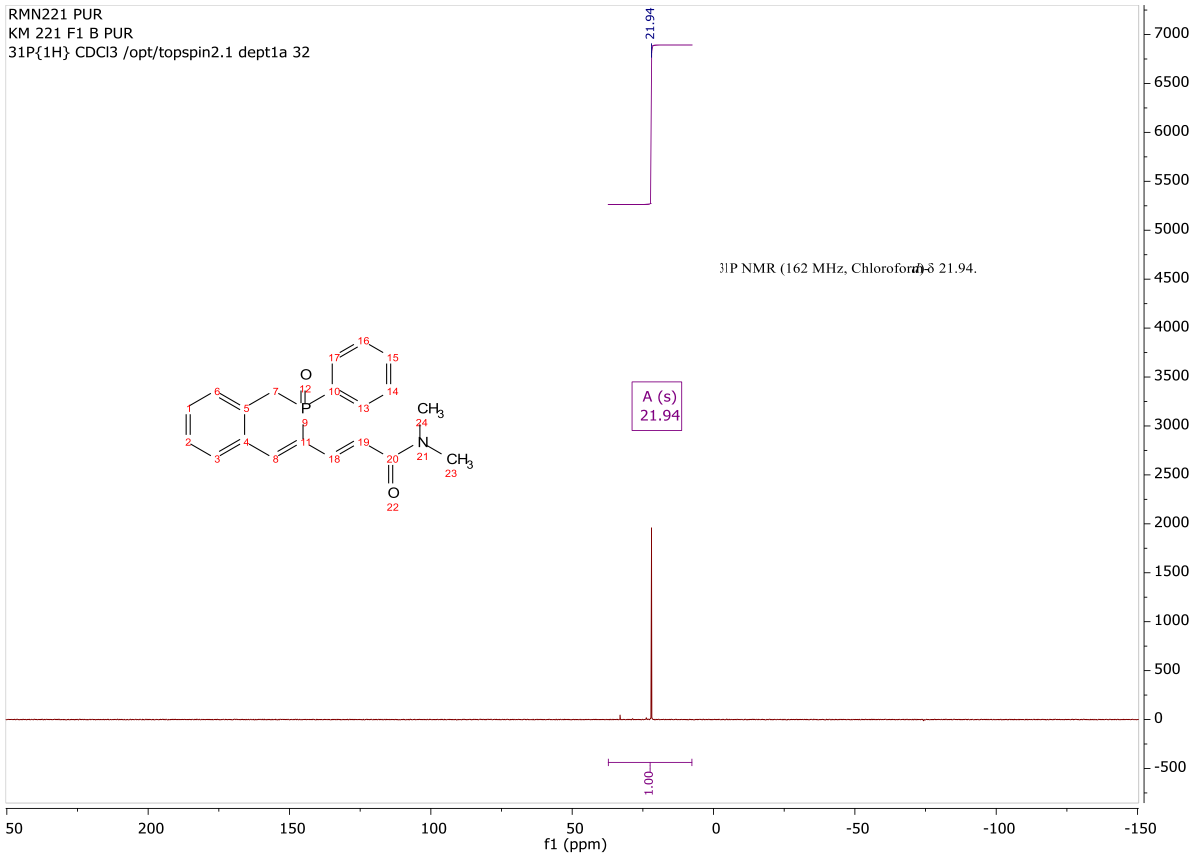

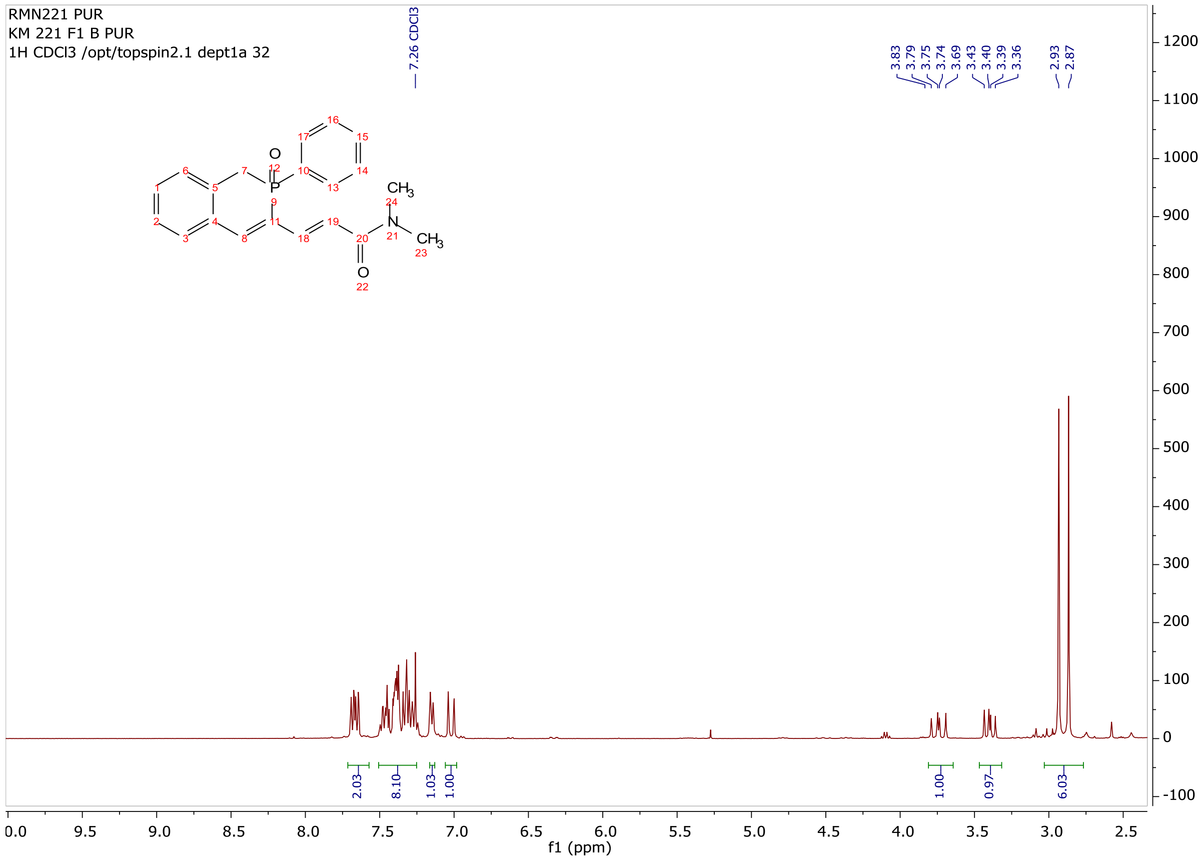

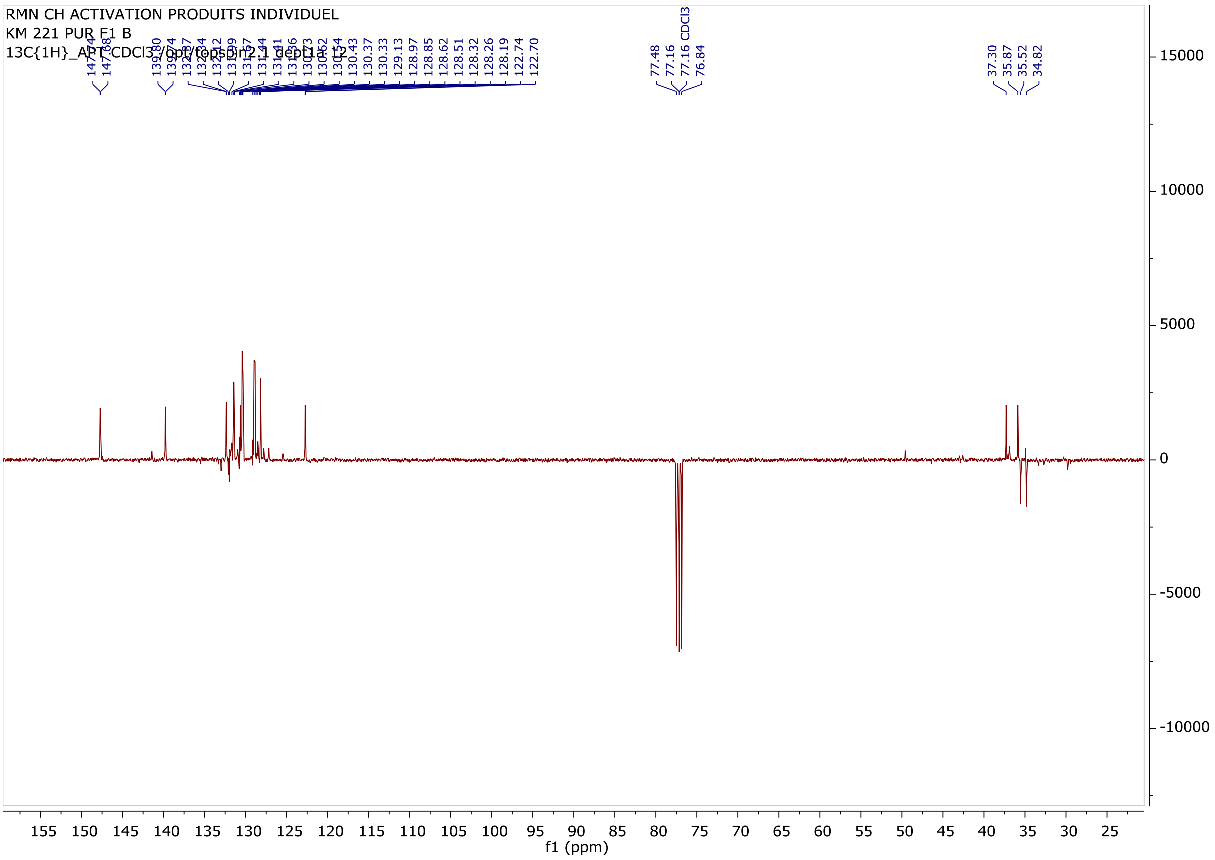

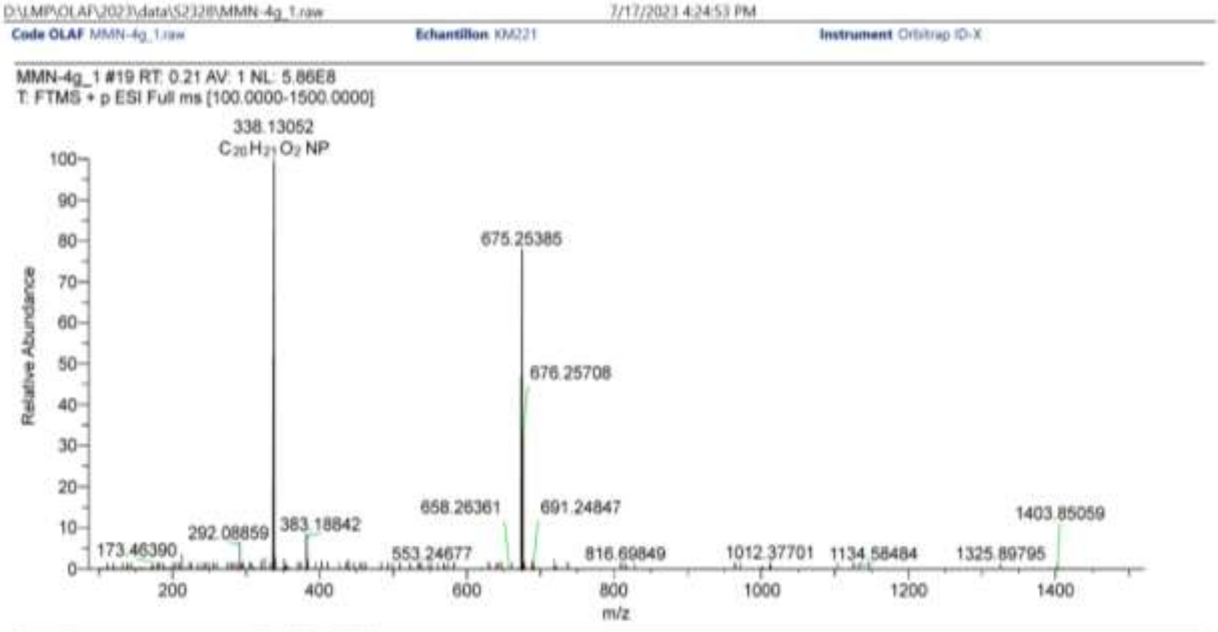

Rank	Peak Mass	Display Formula	Delta [ppm]	Theo. mass	Pattern Cov. [%]	# Matched Iso.	Combined Score	MS Cov. [%]	MSMS Matched...
1	338.13052	$C_{20}H_{21}O_2NP$	0.24	338.13044	99.36	4	95.72	99.26	(Collection)
2	338.13052	[illegible]	-0.33	338.13063	86.31	3	77.47	81.38	(Collection)
3	338.13052	[illegible]	-0.34	338.13064	86.5	3	77.42	81.38	(Collection)

S8: (*E*)-3-(2-oxido-2-phenyl-1*H*-isophosphinolin-3-yl)acrylonitrile **(3h)**

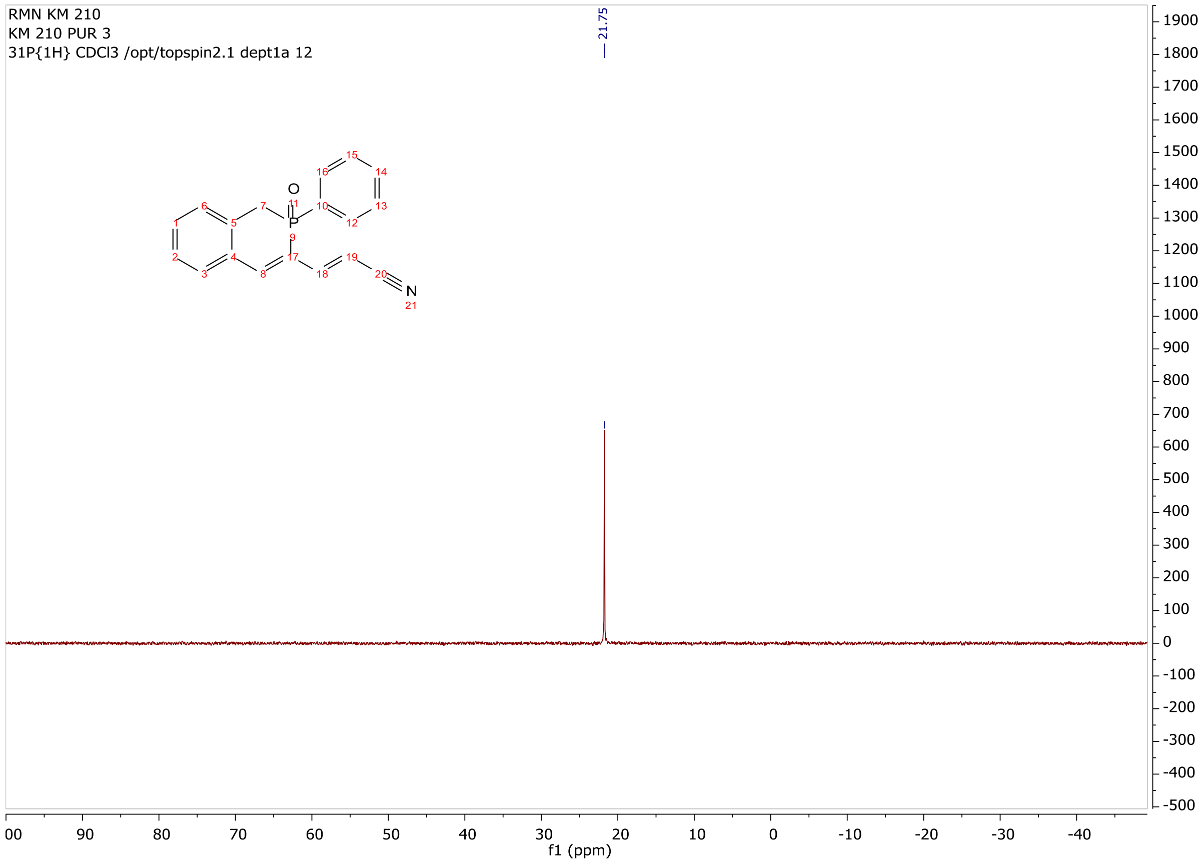

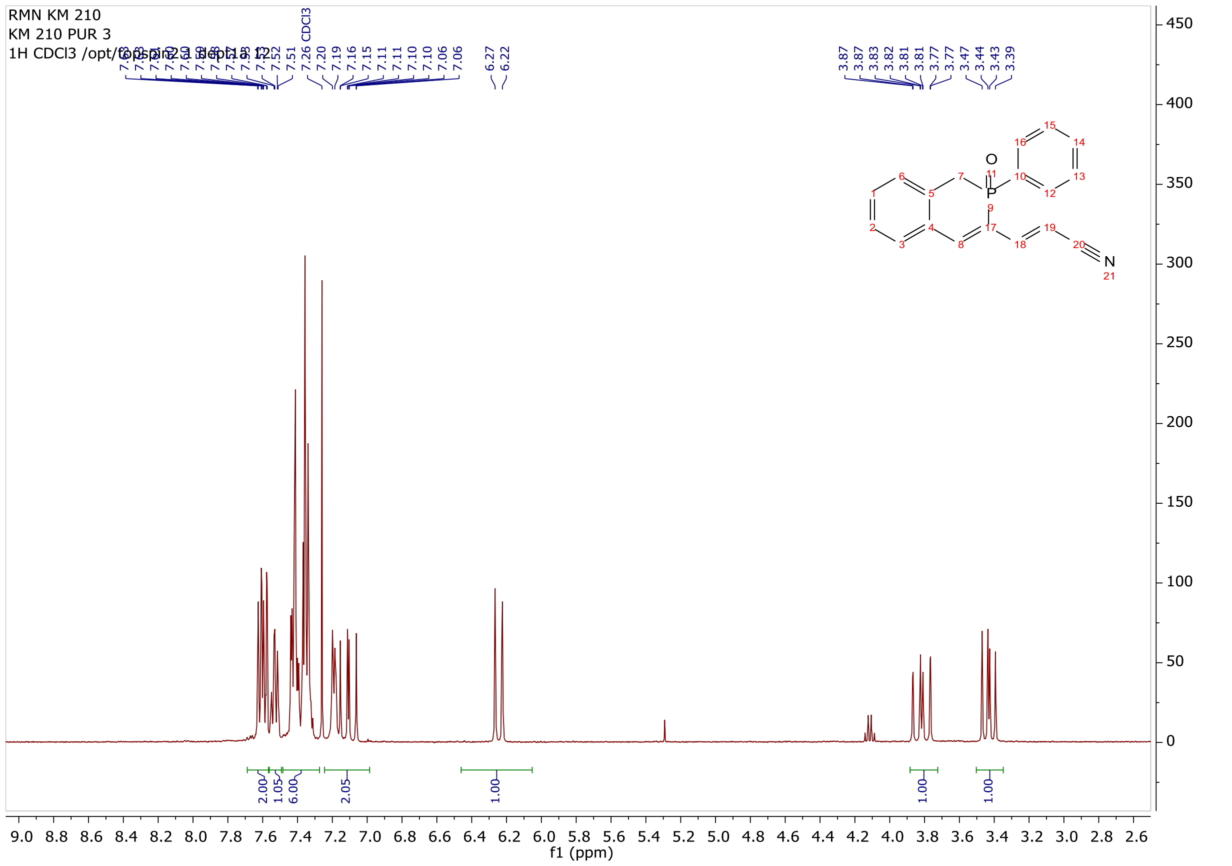

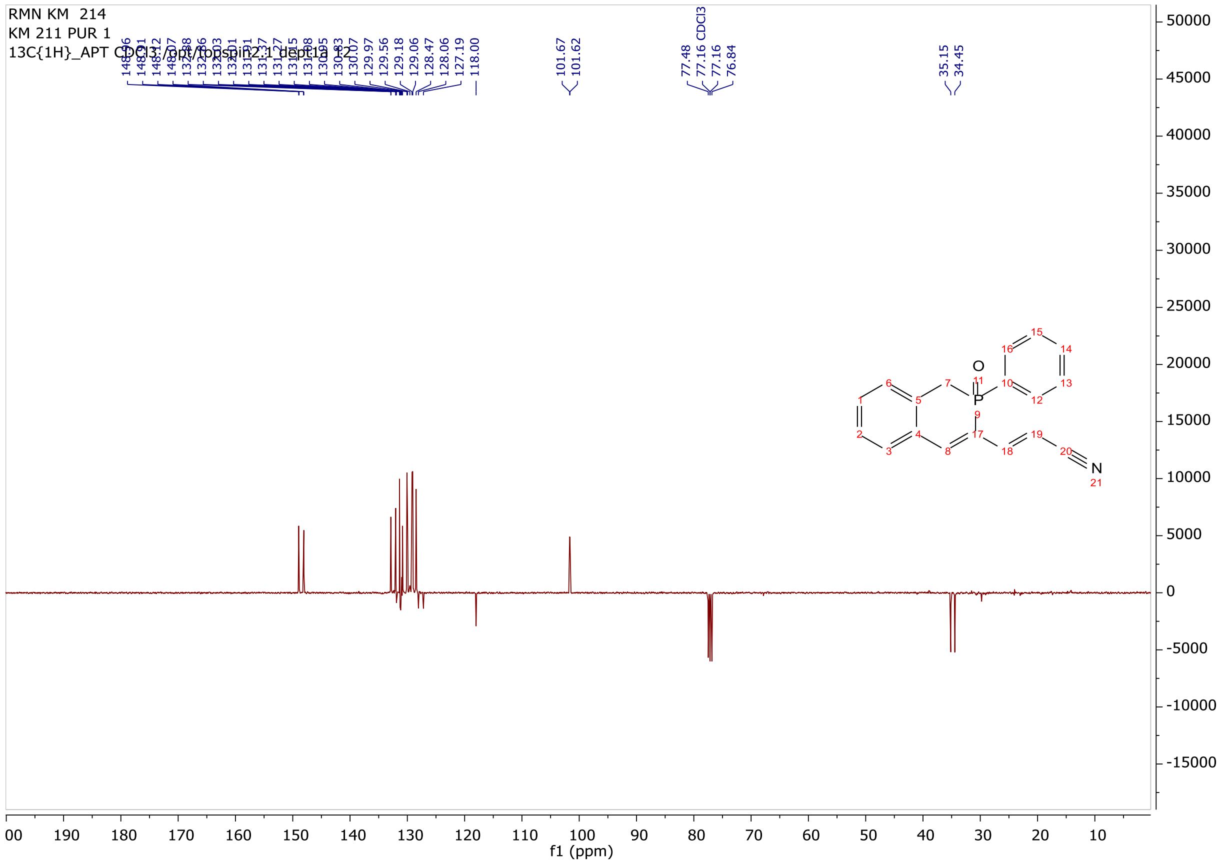

D:\LMP\OLAF\2023\data\S2328\MMN-4f_1.raw 7/17/2023 4:24:23 PM

Code OLAF MMN-4f_1.raw **Echantillon** KM212 **Instrument** Orbitrap ID-X

MMN-4f_1 #19 RT: 0.21 AV: 1 NL: 1.31E9
T: FTMS + p ESI Full ms [100.0000-1500.0000]

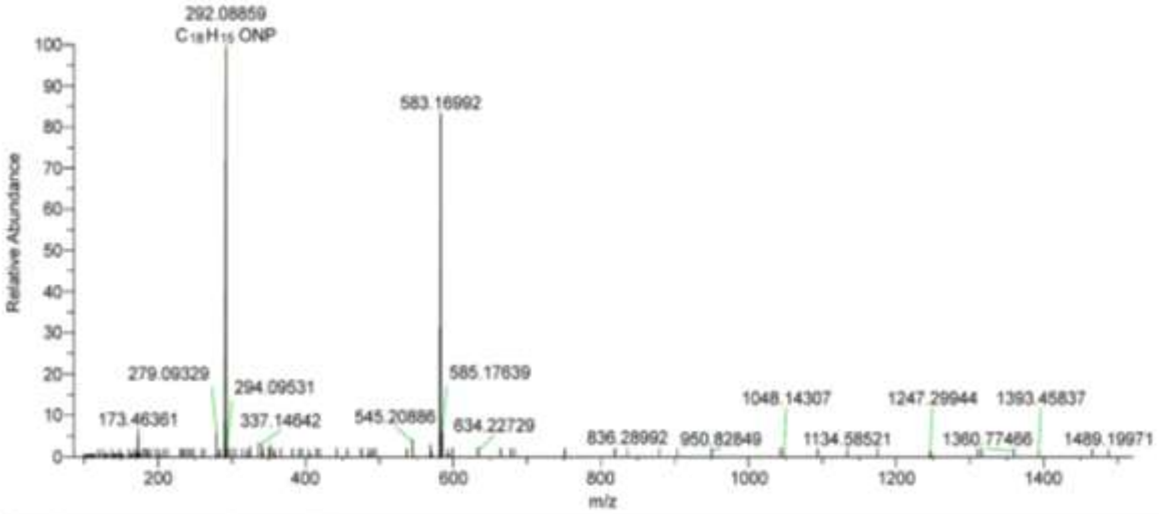

Rank	Peak Mass	Display Formula	Delta [ppm]	Theo. mass	Pattern Cov. [%]	# Matched Iso.	Combined Score	MS Cov. [%]	MSMS Matched...
1	292.08859	$C_{18}H_{15}ONP$	0.05	292.08858	99.7	3	96.39	99.51	(Collection)
2	292.08859	[illegible]	-0.60	292.08877	88.68	1	78.12	81.99	(Collection)
3	292.08859	[illegible]	-0.62	292.08877	89.01	1	78.11	81.99	(Collection)

S9: Methyl 2-methyl-3-(2-oxido-2-phenyl-1*H*-isophosphinolin-3-yl)acrylate **(3ia)**

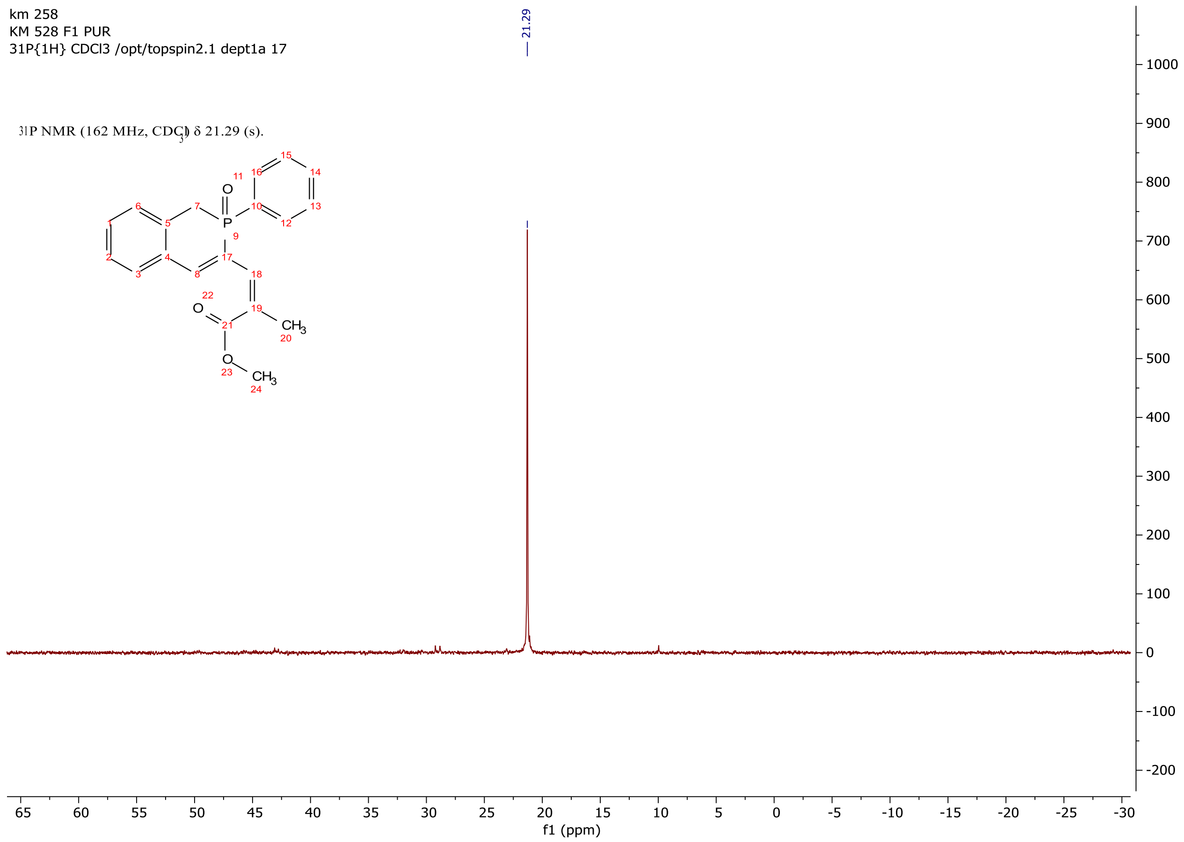

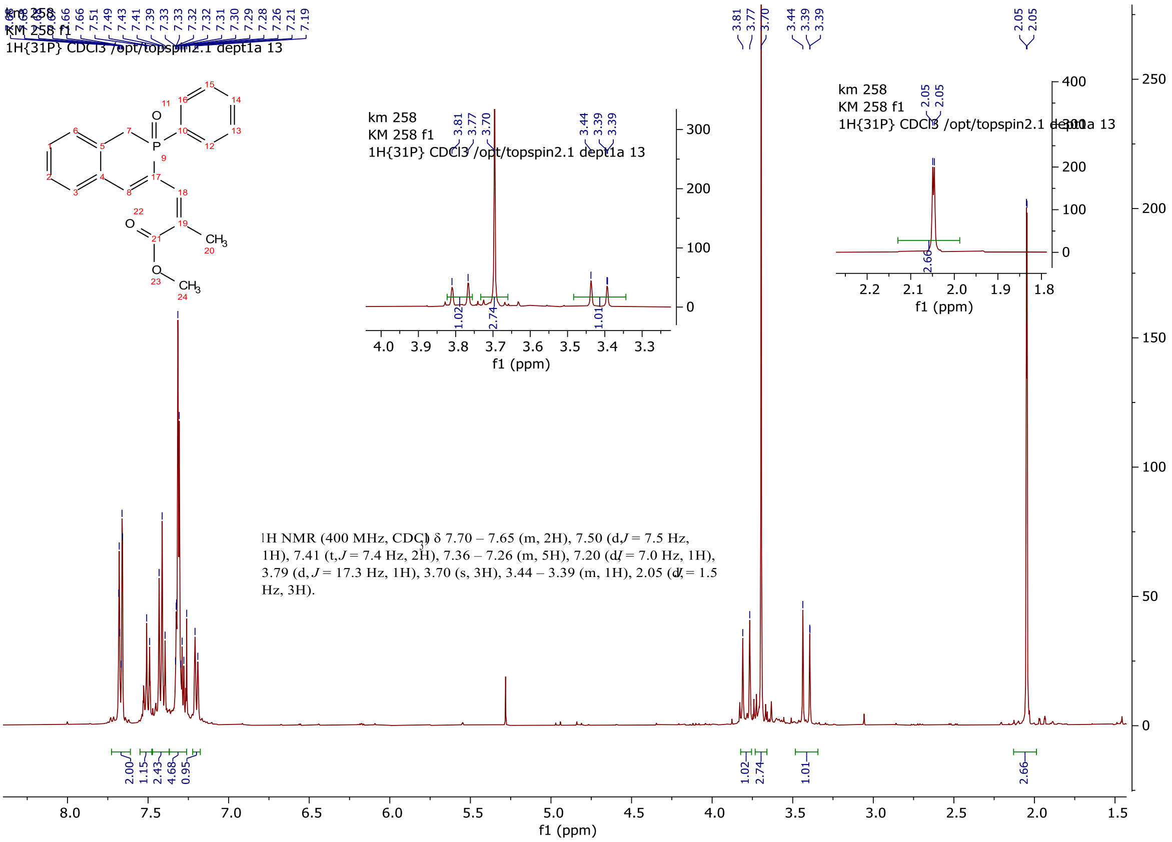
km 258
KM 258 f1
1H{31P} CDCl3 /opt/topspin2.1 dept1a 13
1H NMR (400 MHz, CDCl3) δ 7.70 – 7.65 (m, 2H), 7.50 (d, J = 7.5 Hz, 1H), 7.41 (t, J = 7.4 Hz, 2H), 7.36 – 7.26 (m, 5H), 7.20 (d, J = 7.0 Hz, 1H), 3.79 (d, J = 17.3 Hz, 1H), 3.70 (s, 3H), 3.44 – 3.39 (m, 1H), 2.05 (d, J = 1.5 Hz, 3H).
f1 (ppm)

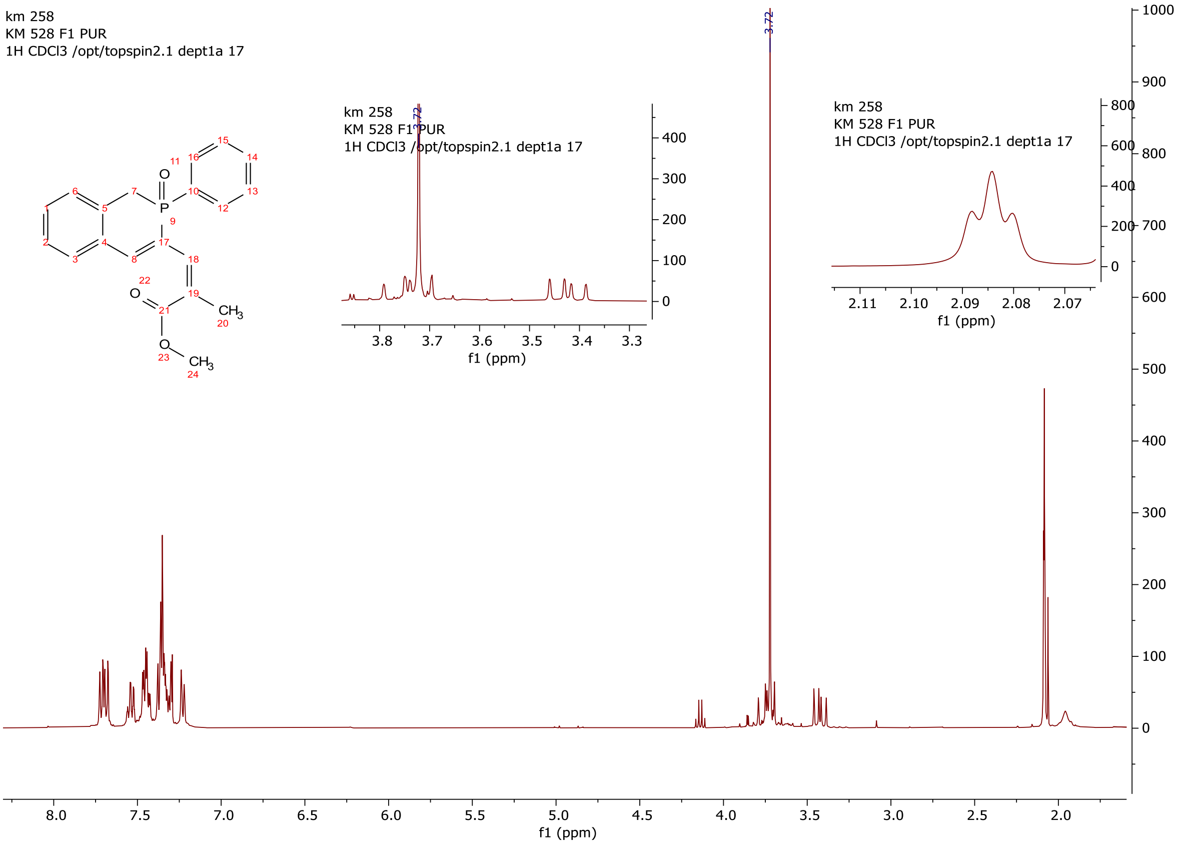
km 258
KM 528 F1 PUR
1H CDCl3 /opt/topspin2.1 dept1a 17
f1 (ppm)

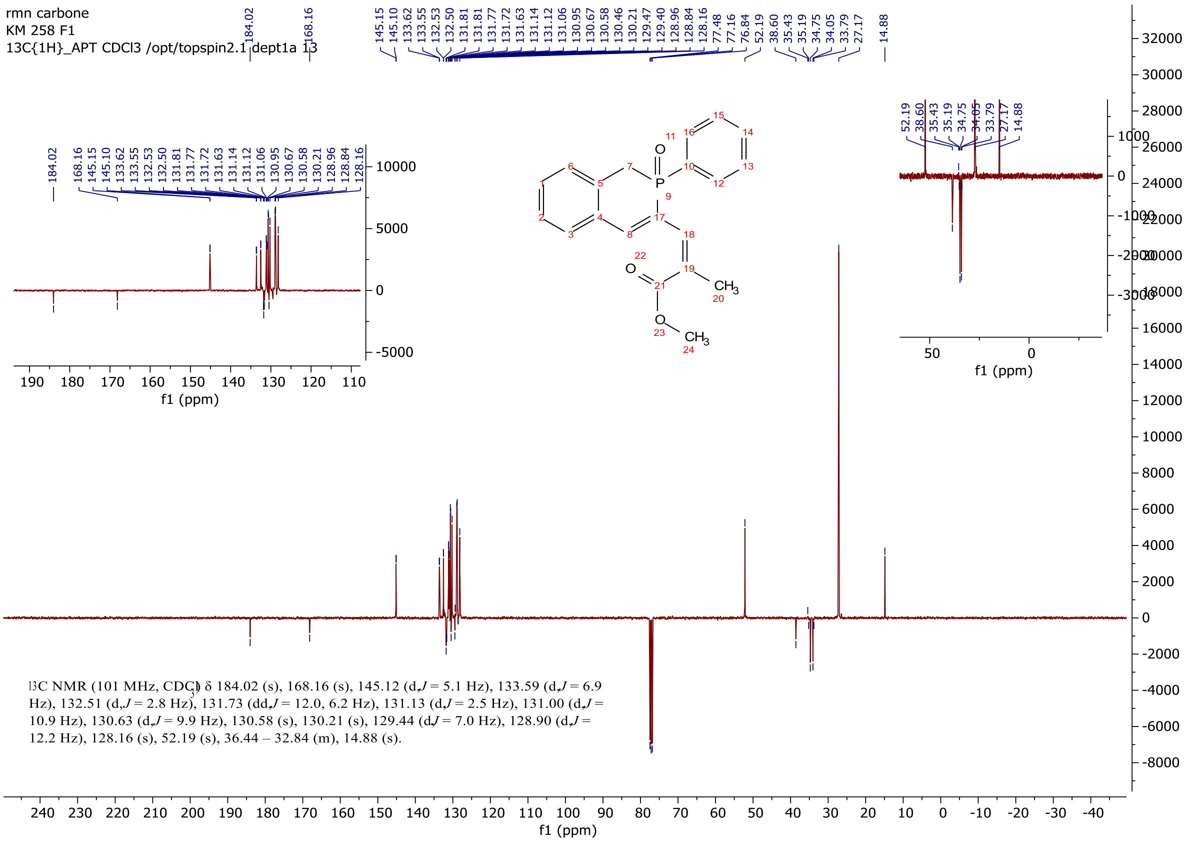
rmn carbone
KM 258 F1
13C{1H}_APT CDCl3 /opt/topspin2.1 dept1a 13
13C NMR (101 MHz, CDCl3) δ 184.02 (s), 168.16 (s), 145.12 (d, J = 5.1 Hz), 133.59 (d, J = 6.9 Hz), 132.51 (d, J = 2.8 Hz), 131.73 (dd, J = 12.0, 6.2 Hz), 131.13 (d, J = 2.5 Hz), 131.00 (d, J = 10.9 Hz), 130.63 (d, J = 9.9 Hz), 130.58 (s), 130.21 (s), 129.44 (d, J = 7.0 Hz), 128.90 (d, J = 12.2 Hz), 128.16 (s), 52.19 (s), 36.44 – 32.84 (m), 14.88 (s).
f1 (ppm)

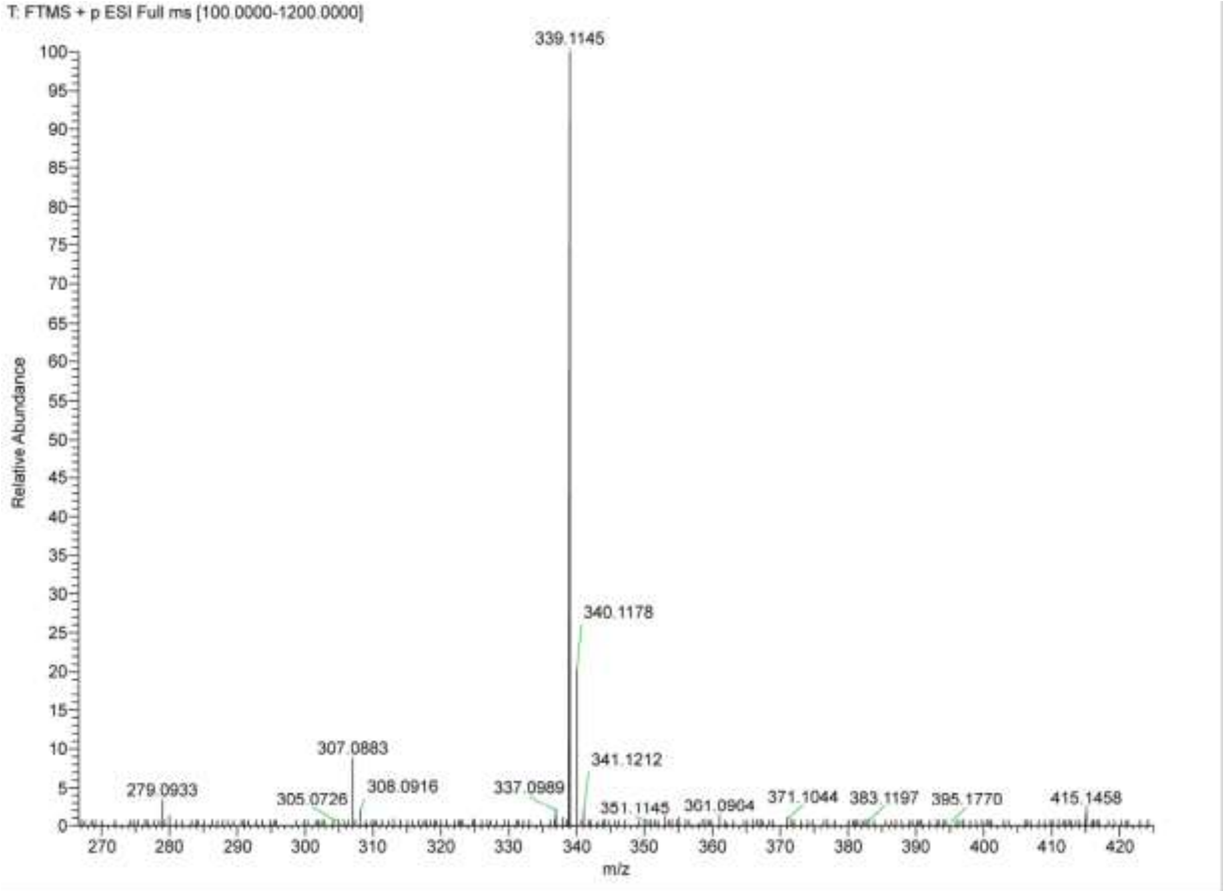

D:\LMP\OLAF\2024\data\S2408\MMN-7a.raw 2/20/2024 11:48:10 AM

Rank	Peak Mass	Display Formula	Delta [ppm]	Theo. mass	Pattern Cov. [%]	# Matched Iso.	Combined Score	MS Cov. [%]	MSMS Matched Fragments
1	339.1145	$C_{[illegible]}H_{[illegible]}O_{3}P$	0.12	339.11446	97.32	6	93.75	98.1	(Collection)
2	339.1145	$C_{[illegible]}H_{[illegible]}O_{[illegible]}NP_{[illegible]}$	-0.80	339.11477	84.45	5	78.35	82.2	(Collection)

S10: Methyl 2-((2-oxido-2-phenyl-1*H*-isophosphinolin-3-yl)methyl)acrylate **(3ib)**

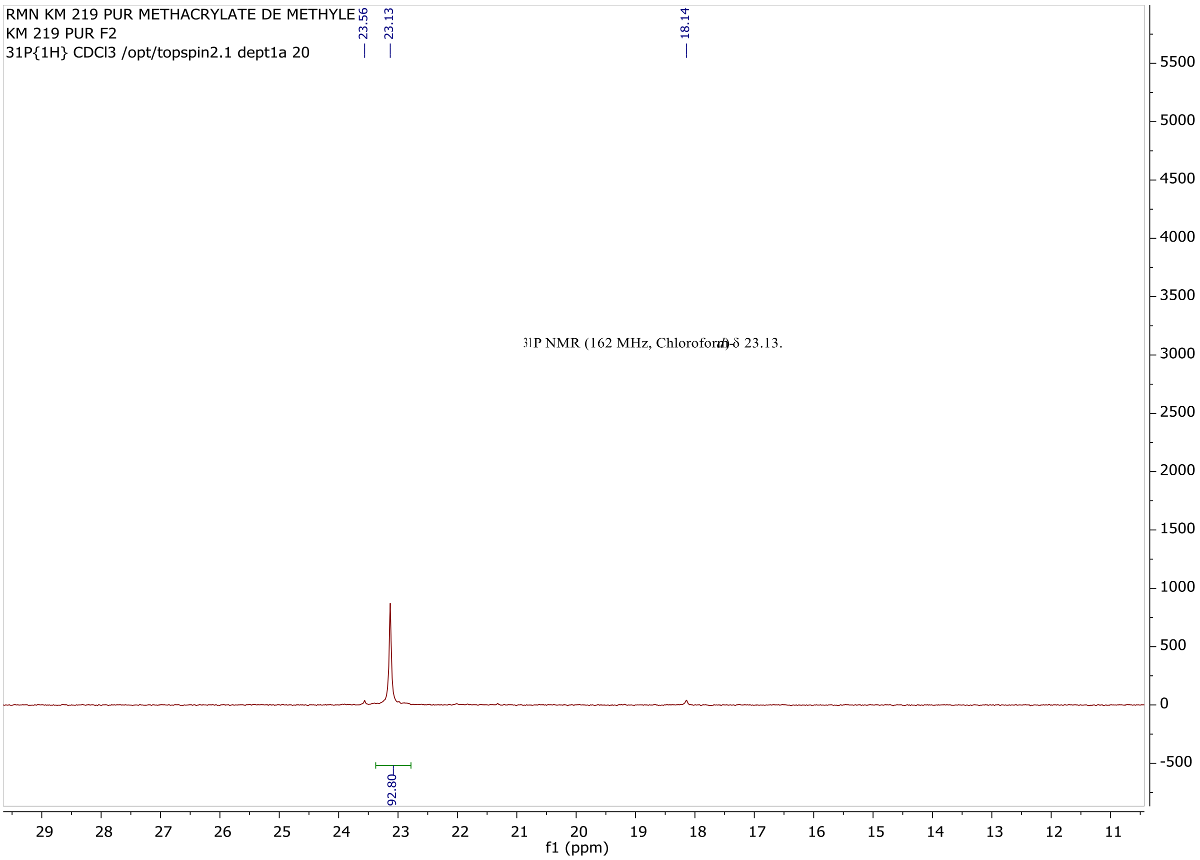

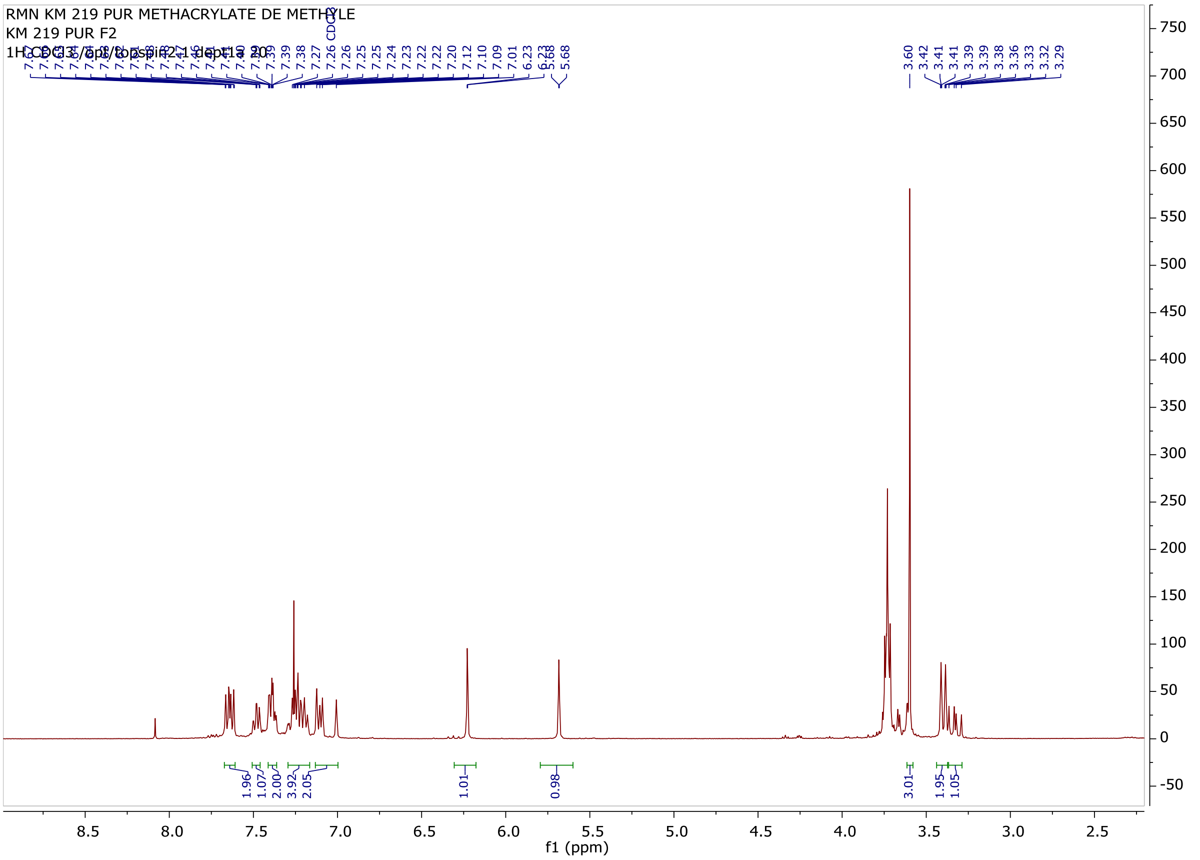

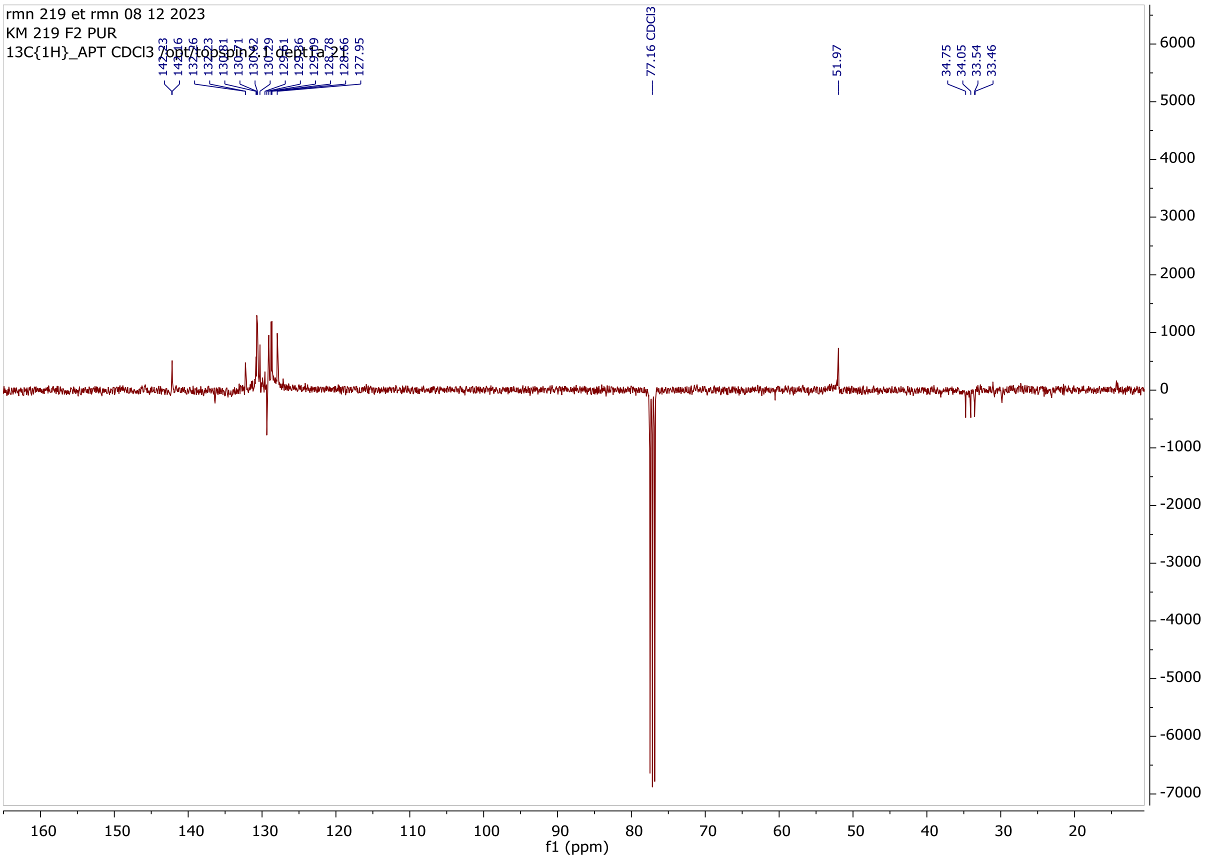

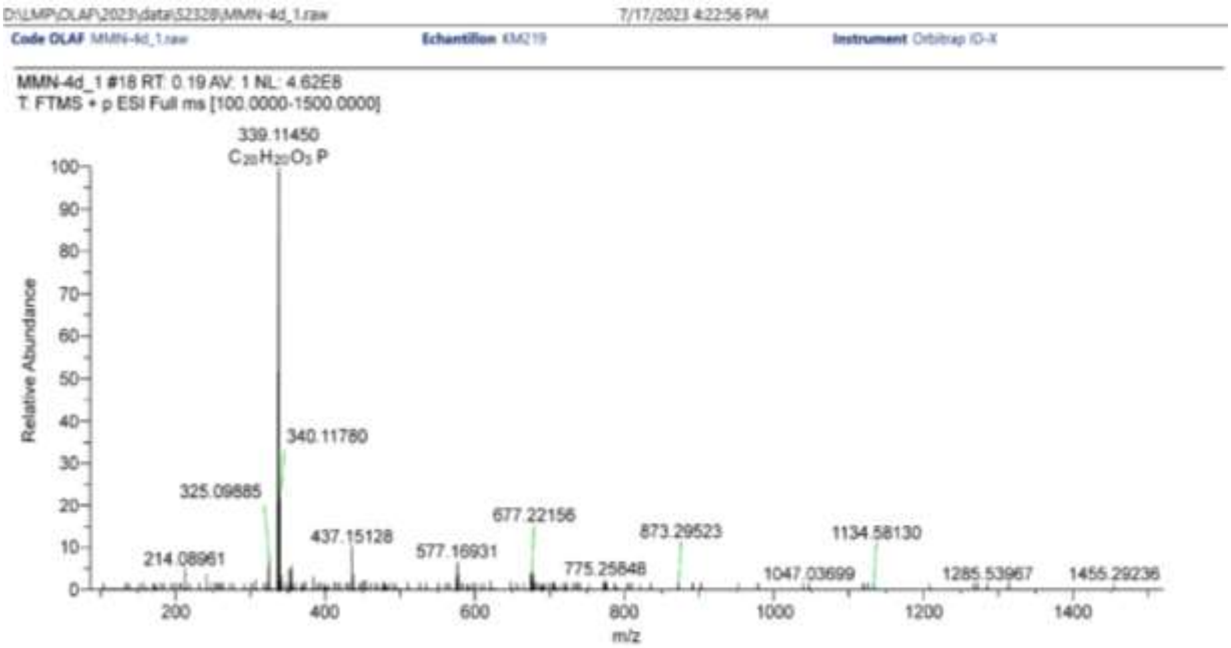

Rank	Peak Mass	Display Formula	Delta [ppm]	Theo. mass	Pattern Cov. [%]	# Matched Iso.	Combined Score	MS Cov. [%]	MSMS Matched...
1	339.11450	$C_{20}H_{20}O_3P$	0.13	339.11446	99.1	5	95.58	99.57	(Collection)
2	339.11450	[illegible]	-0.43	339.11465	86.46	2	76.75	80.64	(Collection)
3	339.11450	[illegible]	-0.45	339.11465	86.64	4	76.7	80.64	(Collection)

S11: Methyl 3-(2-oxido-2-phenyl-1*H*-isophosphinolin-3-yl)-2-((2-oxido-2-phenyl-1*H*-isophosphinolin-3-yl)methyl)acrylate **(3ic)**

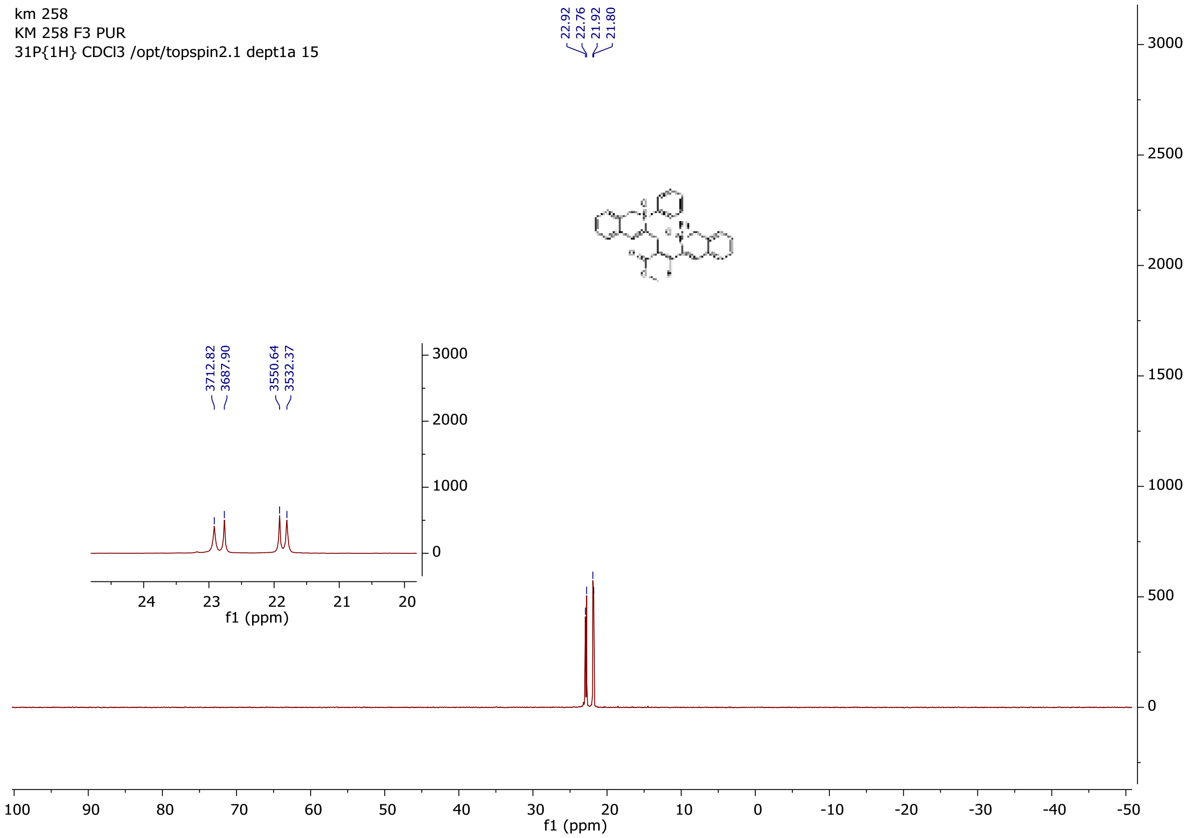

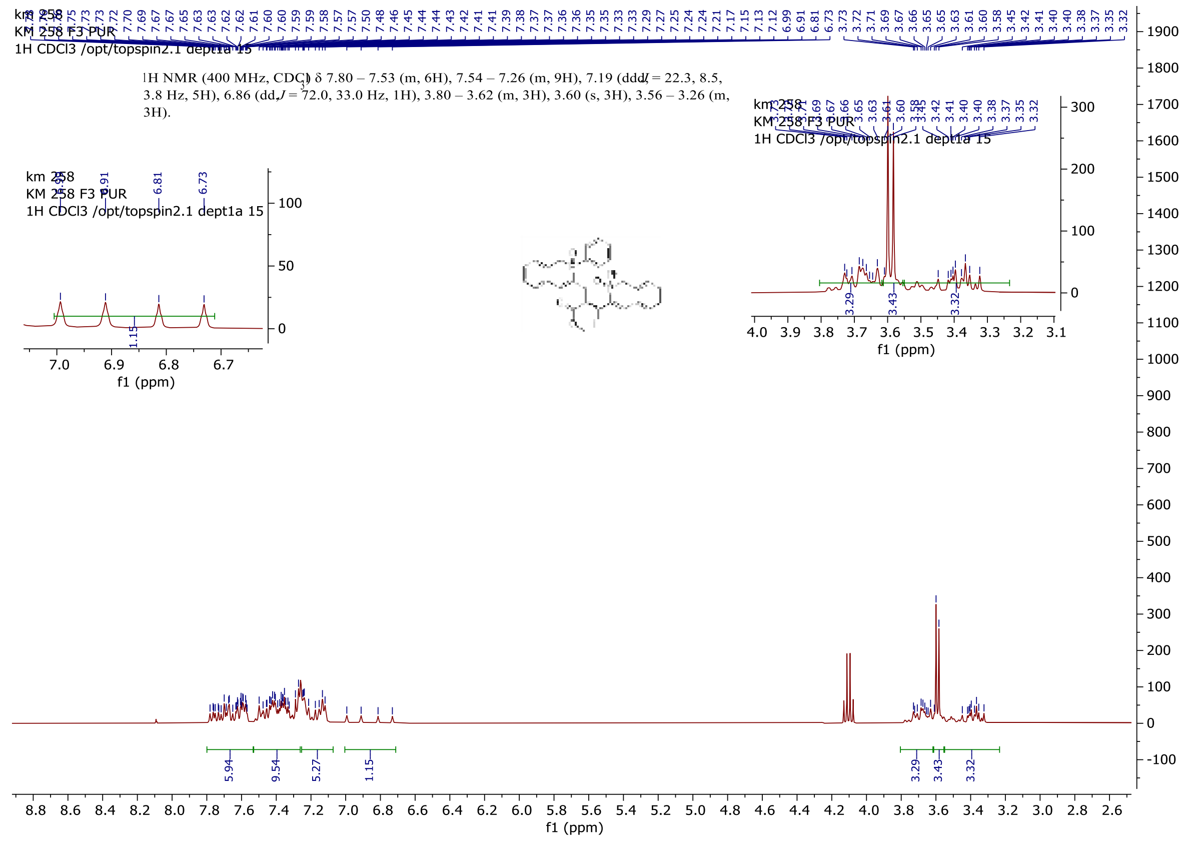

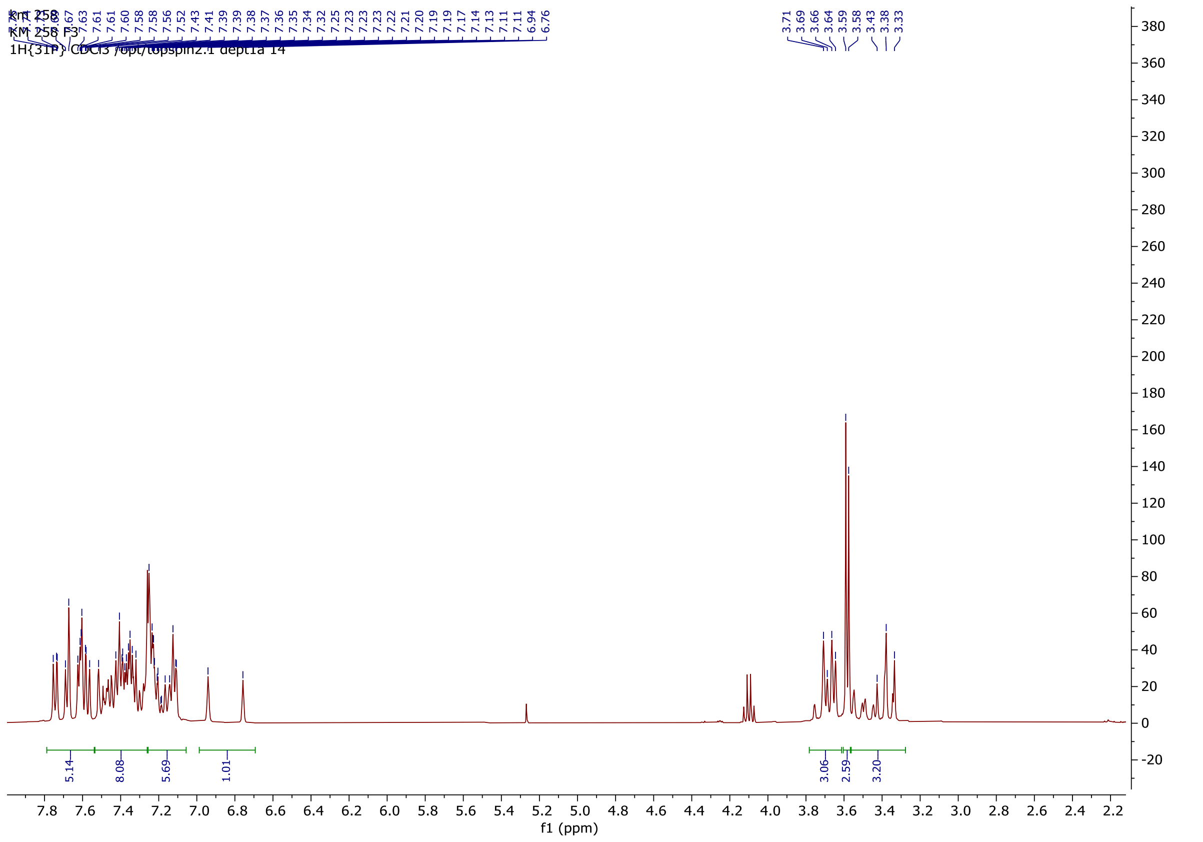

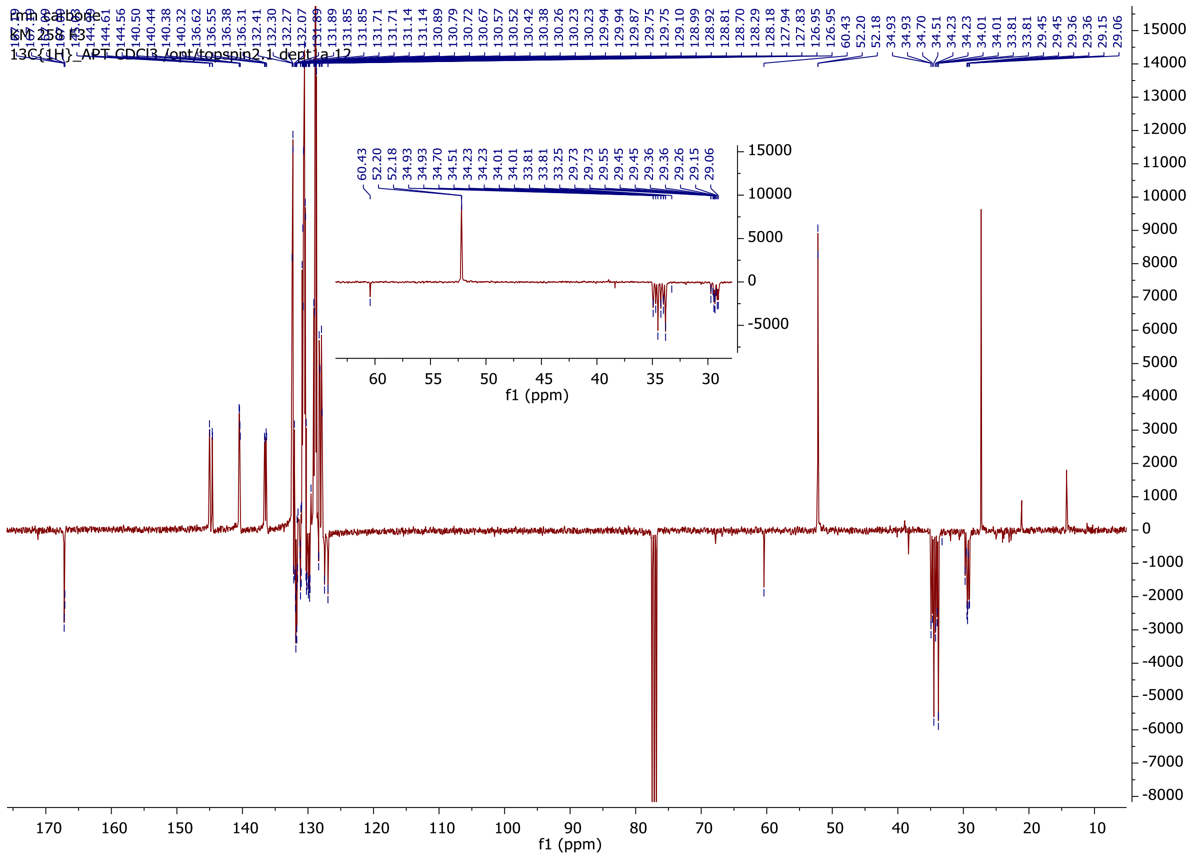

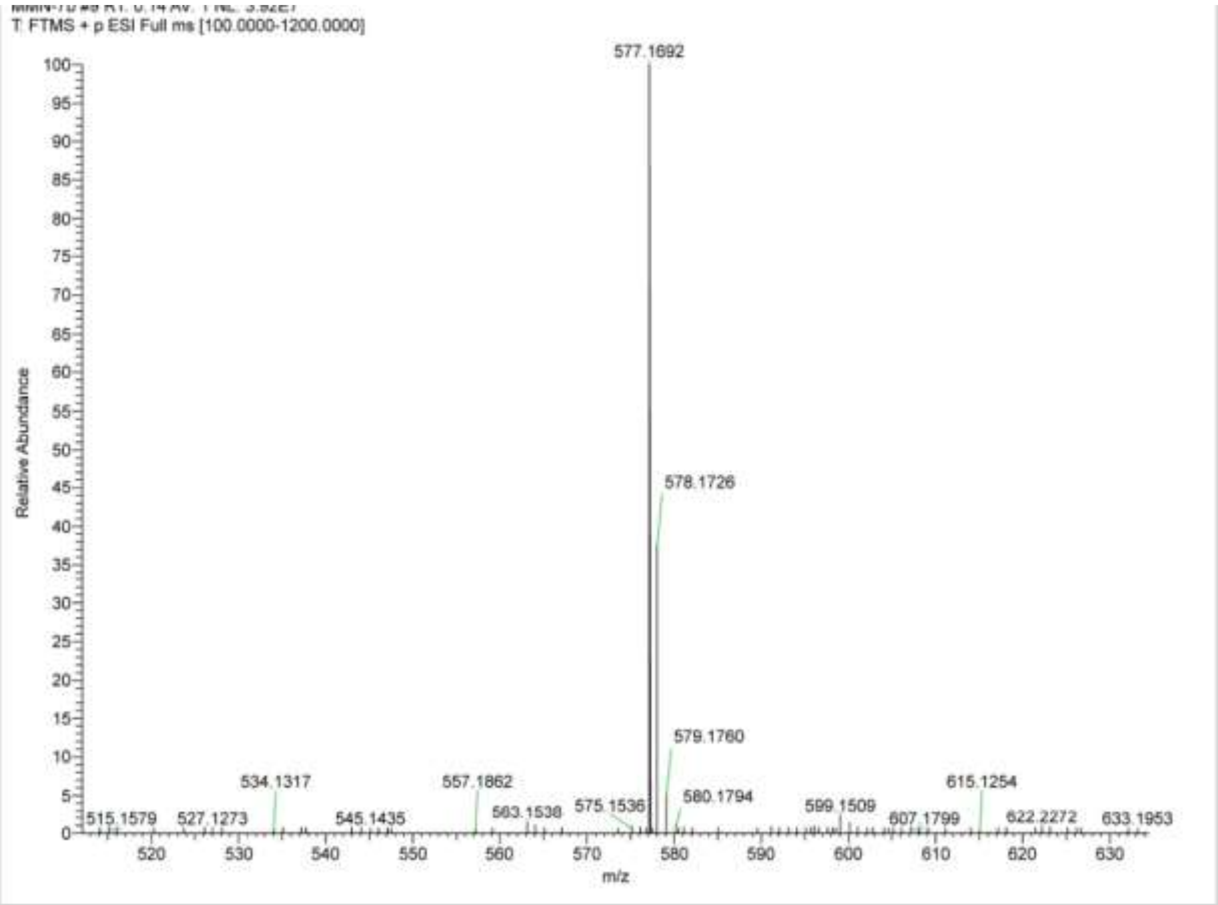

D:\LMP\OLAF\2024\data\S2408\MMN-7b.raw 2/20/2024 11:48:49 AM

Rank	Peak Mass	Display Formula	Delta [ppm]	Theo. mass	Pattern Cov. [%]	# Matched Iso.	Combined Score	MS Cov. [%]	MSMS Matched Fragments
1	577.1694	[illegible]	0.39	577.16921	94.42	5	92.34	96.22	(Collection)
3	577.1694	[illegible]	0.97	577.16888	87.15	1	66.89	70.25	(Collection)
8	577.1694	[illegible]	-0.49	577.16972	76.68	5	66.75	70.25	(Collection)

S12: Ethyl 2-((2-oxido-2-phenyl-1H-isophosphinolin-3-yl)methyl)acrylate **(3ja)**

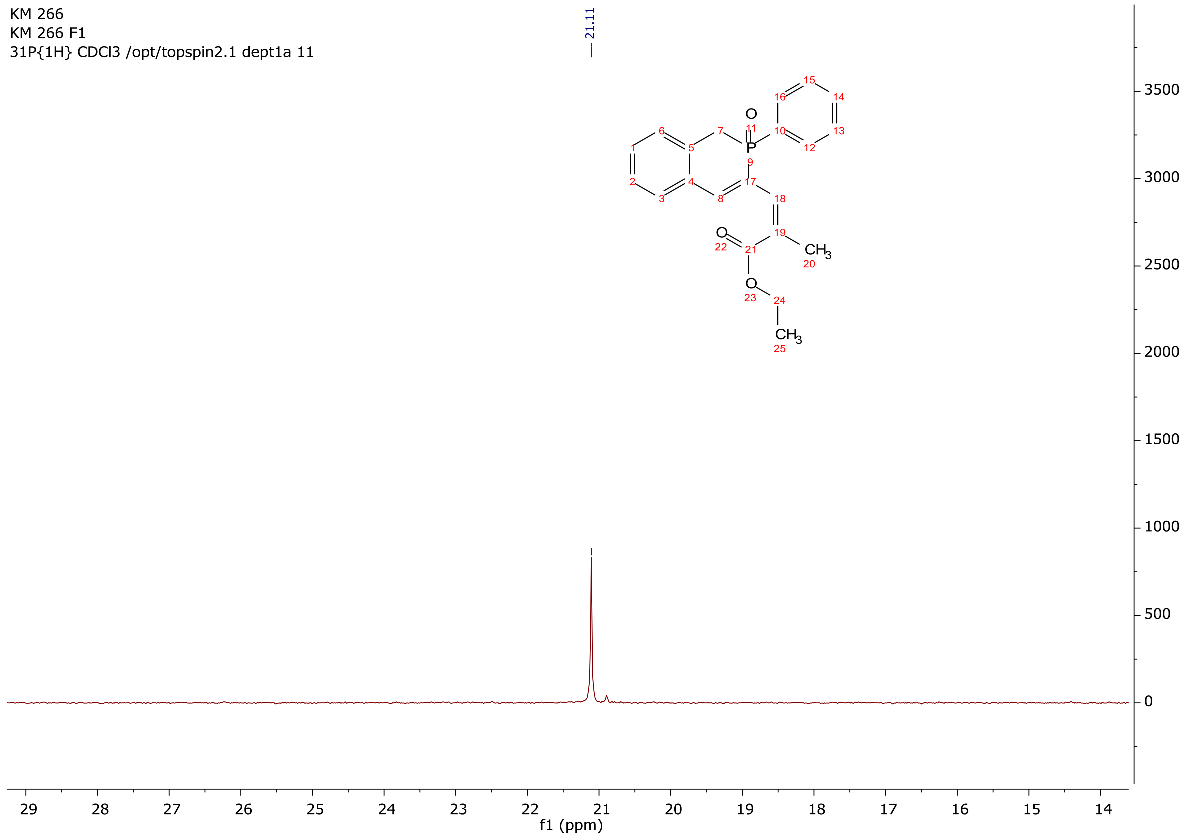

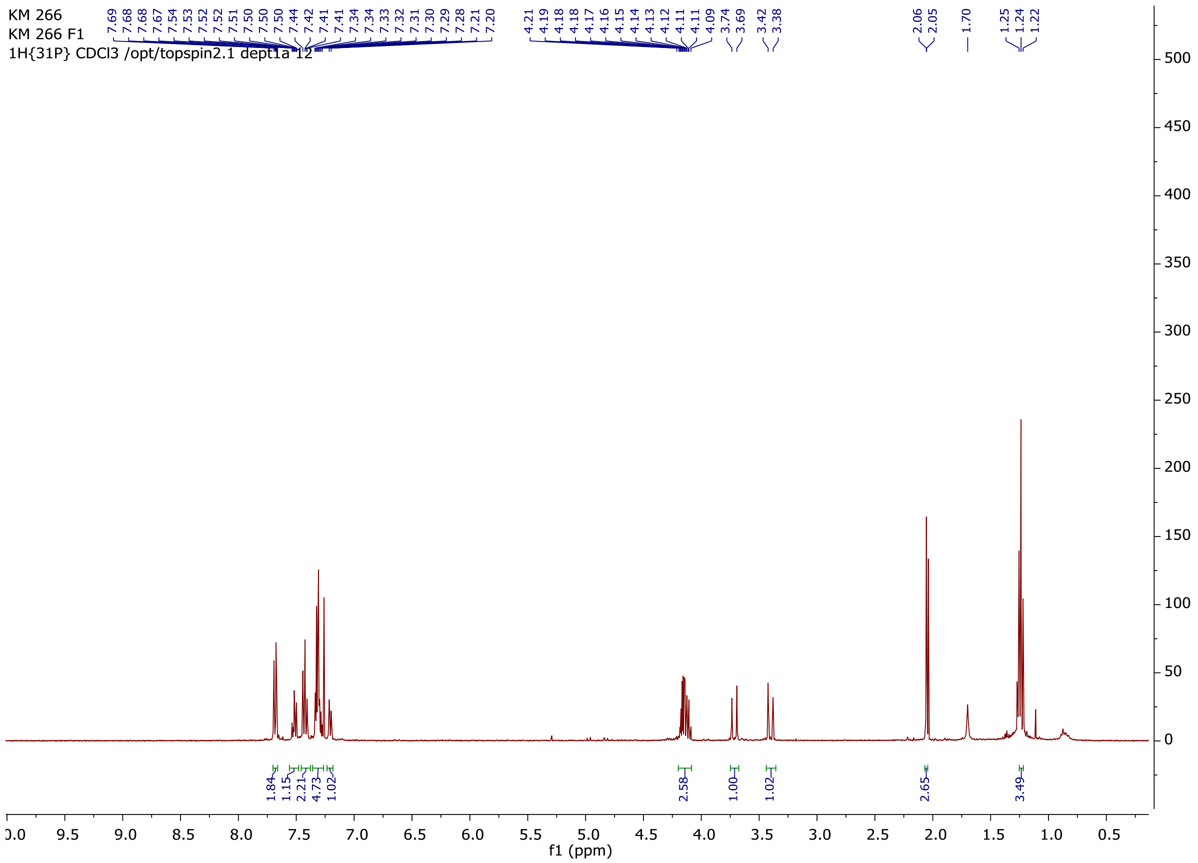
KM 266
KM 266 F1
1H{31P} CDCl3 /opt/topspin2.1 dept1a 12
f1 (ppm)

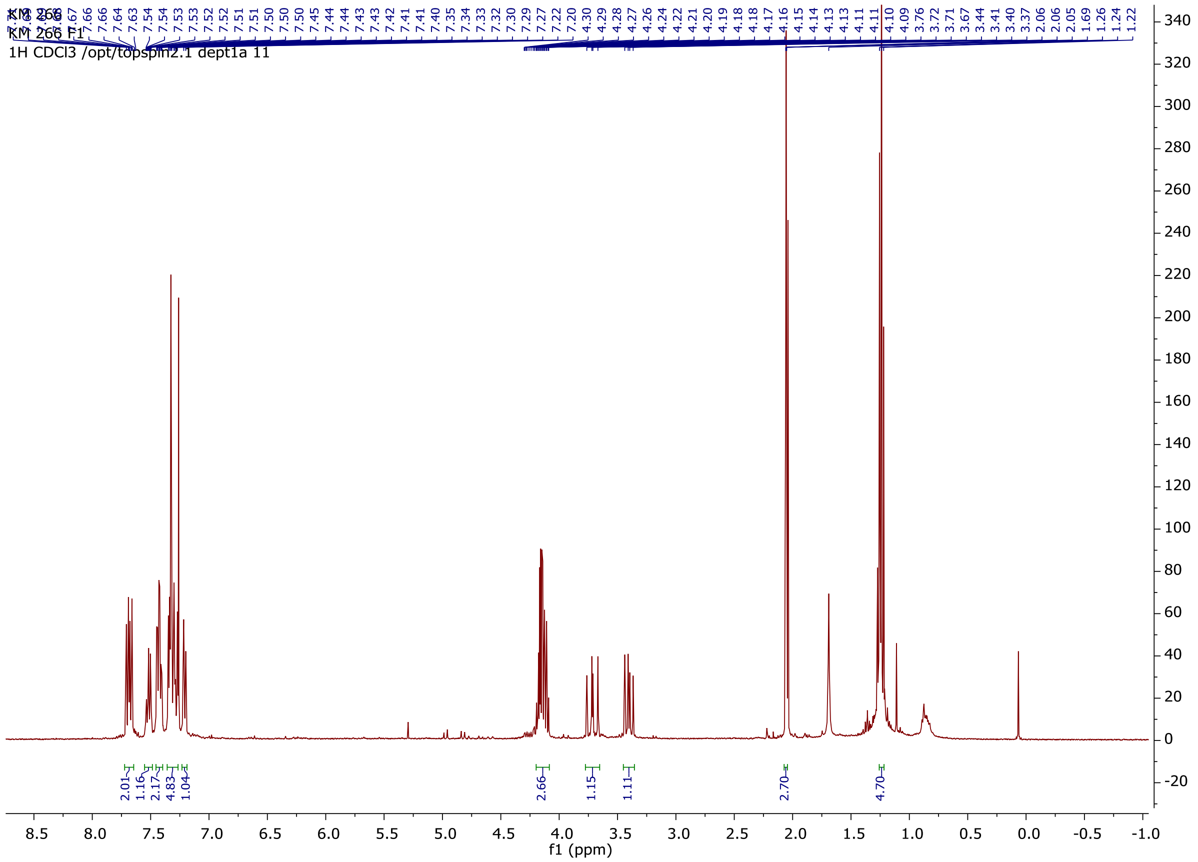
KM 266
KM 266 F1
1H CDCl3 /opt/topspin2.1 dept1a 11
f1 (ppm)

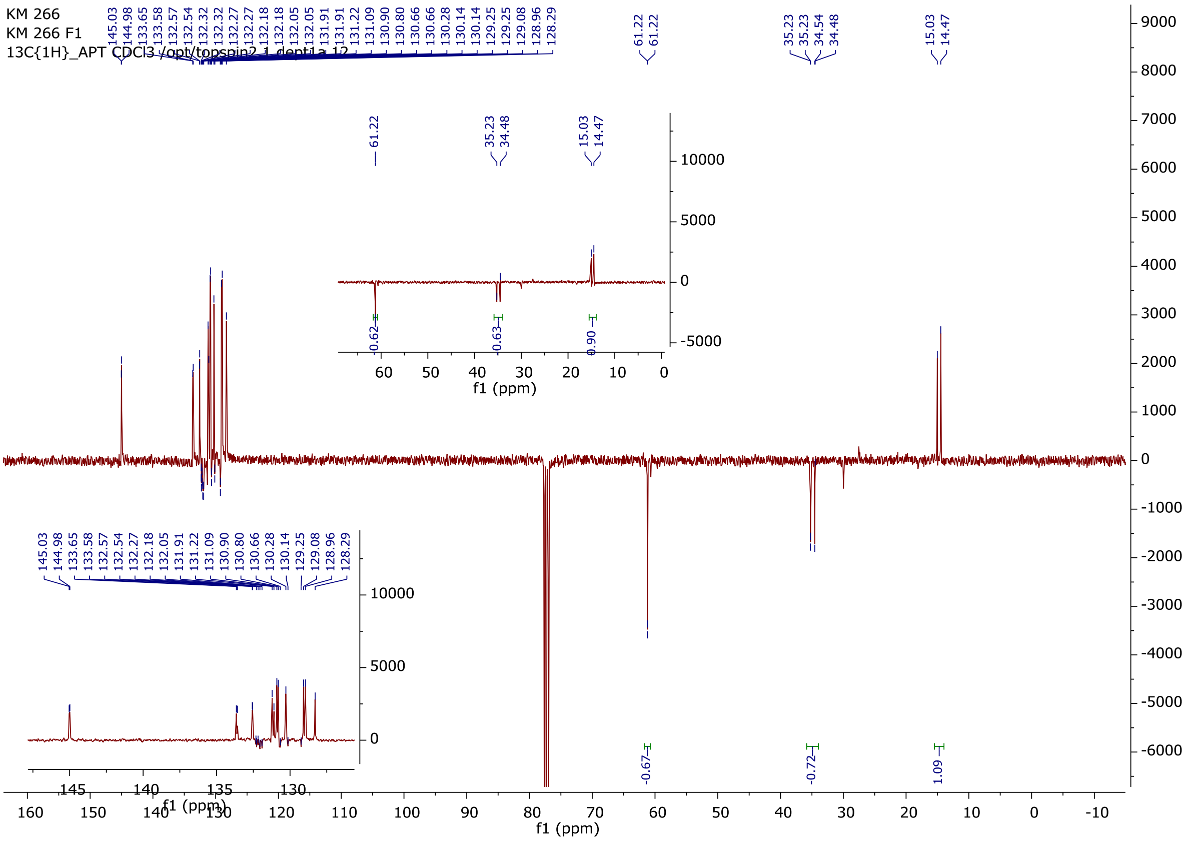

High Resolution Mass Result

Analysis Info

Sample Name **MMN-9 a _ KM-25**

Acquisition Date 3/11/2024 1:10:15 PM

Instrument / Ser# micrOTOF-Q 228888.10300

Acquisition Parameter

Source Type ESI Ion Polarity Positive Scan Begin 50 m/z Scan End 3000 m/z

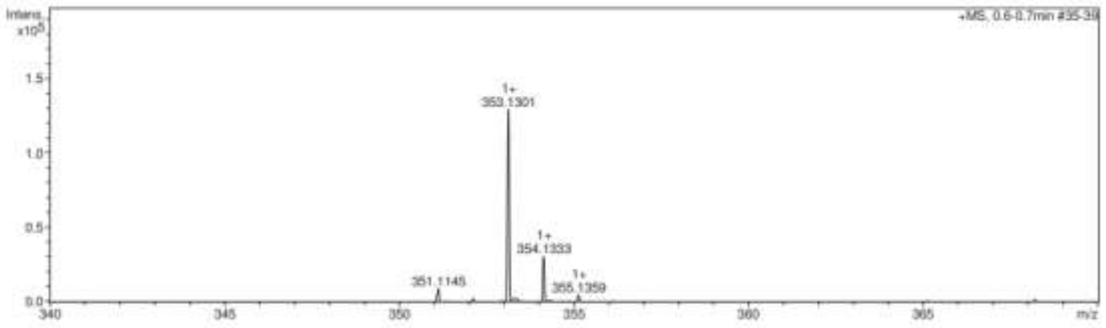

Meas. m/z	#	Ion Formula	Score	m/z	err [mDa]	err [ppm]	mSigma	rdb	e⁻ Conf	N-Rule	Adduct
353.1301	1	C21H22O3P	100.00	353.1301	-0.0	-0.0	2.8	11.5	even	ok	M+H

S13: Ethyl 2-((2-oxido-2-phenyl-1*H*-isophosphinolin-3-yl)methyl)acrylate **(3jb)**

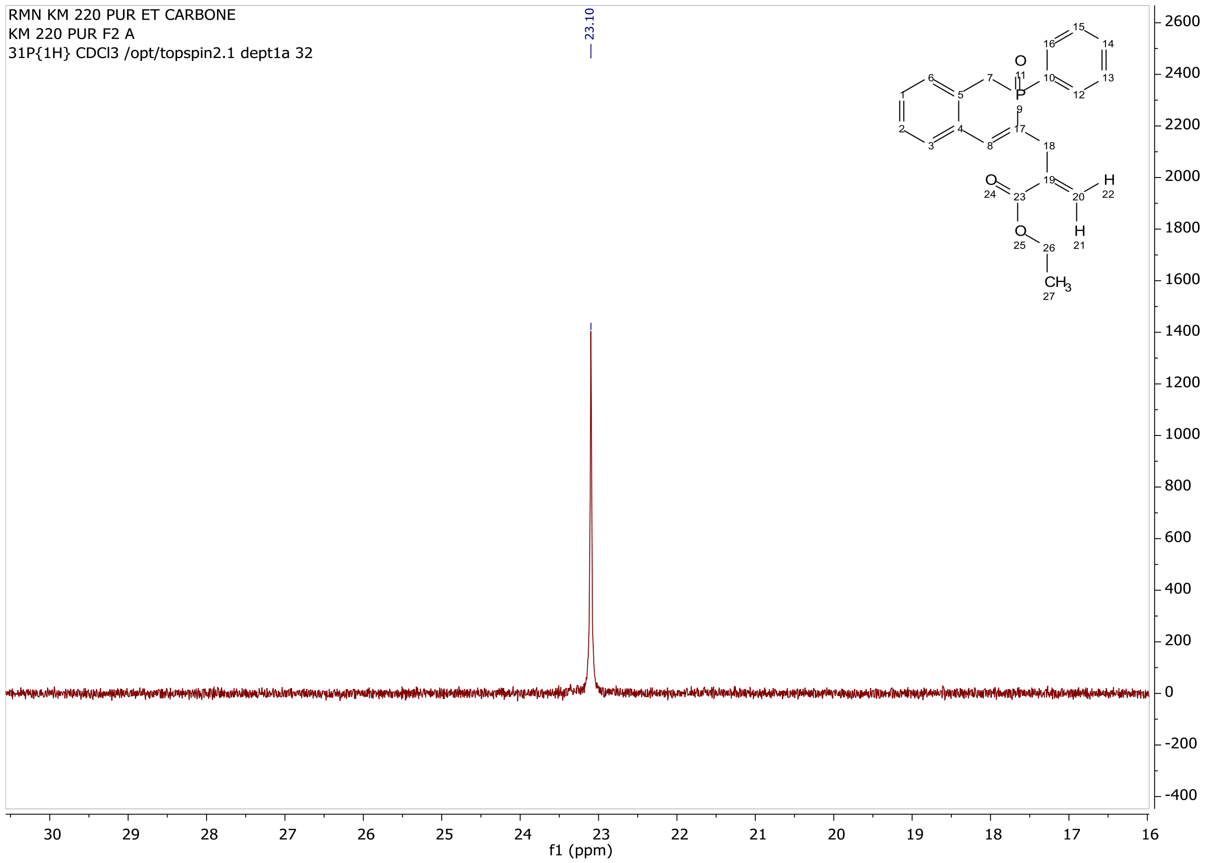

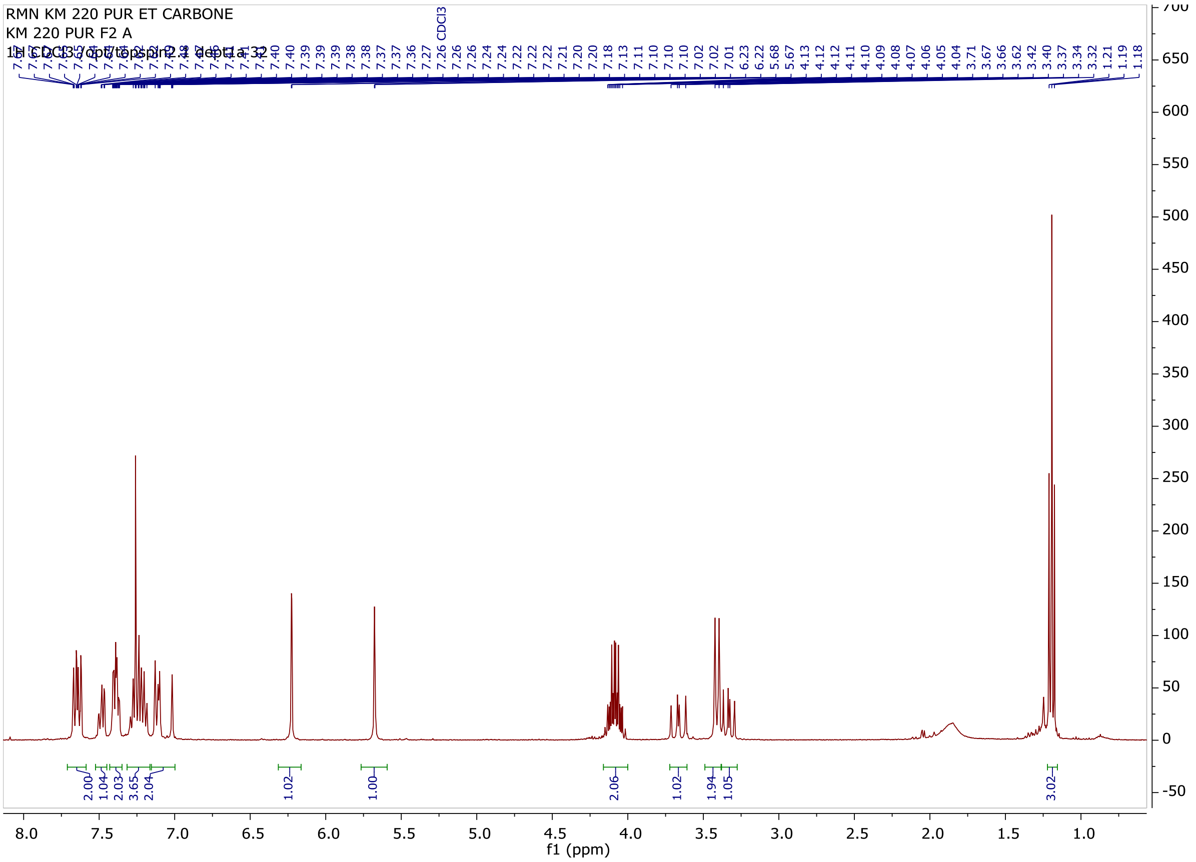
RMN KM 220 PUR ET CARBONE
KM 220 PUR F2 A
CDCl3
7.40
7.39
7.39
7.39
7.38
7.37
7.37
7.36
7.27
7.26
7.26
7.26
7.24
7.24
7.22
7.22
7.22
7.21
7.20
7.20
7.18
7.13
7.11
7.10
7.10
7.10
7.02
7.02
7.01
6.23
6.22
5.68
5.67
4.13
4.12
4.12
4.11
4.10
4.09
4.08
4.07
4.06
4.05
4.04
3.71
3.67
3.66
3.62
3.42
3.40
3.37
3.34
3.32
1.21
1.19
1.18
2.00
1.04
2.03
3.65
2.04
1.02
1.00
2.06
1.02
1.94
1.05
3.02
f1 (ppm)

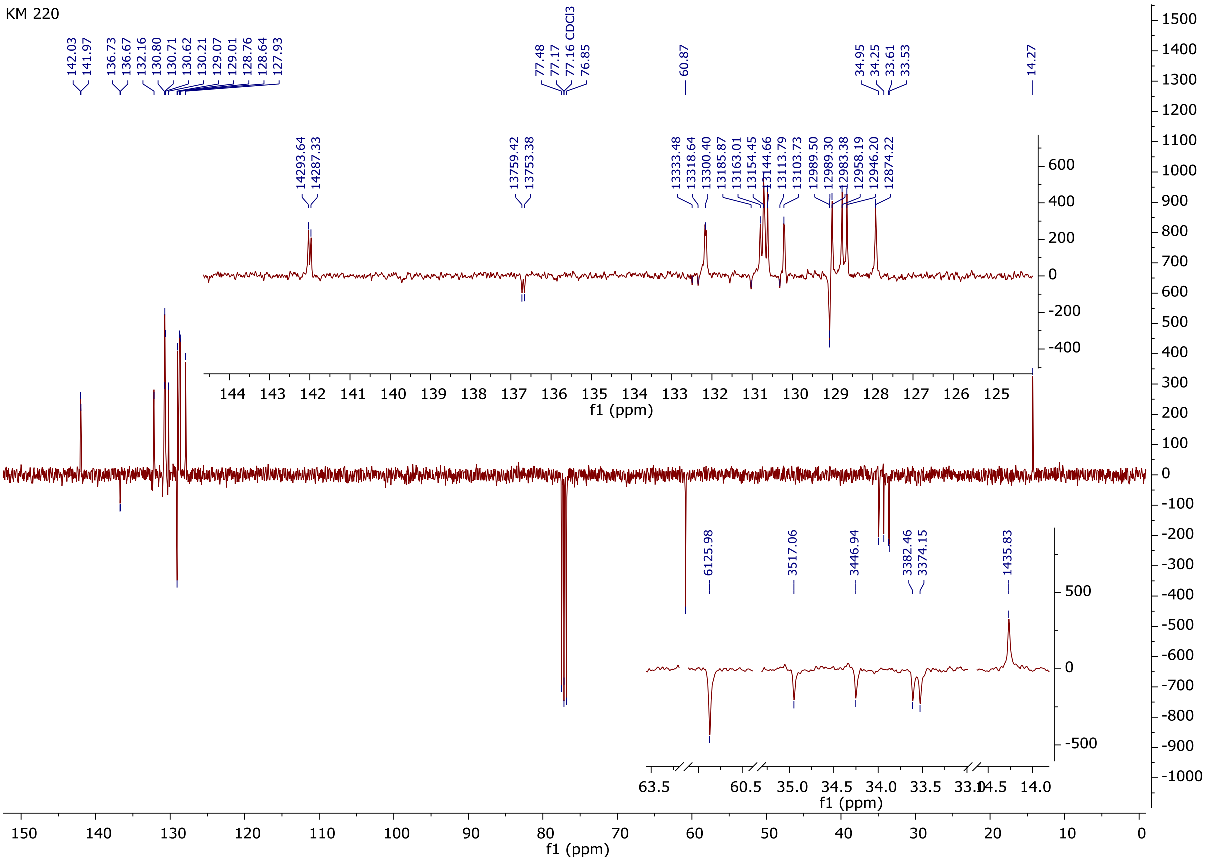
KM 220
142.03
141.97
136.73
136.67
132.16
130.80
130.71
130.62
130.21
129.07
129.01
128.76
128.64
127.93
77.48
77.17
77.16 CDCl3
76.85
60.87
34.95
34.25
33.61
33.53
14.27
14293.64
14287.33
13759.42
13753.38
13333.48
13318.64
13300.40
13185.87
13163.01
13154.45
13144.66
13113.79
13103.73
12989.50
12989.30
12983.38
12958.19
12946.20
12874.22
6125.98
3517.06
3446.94
3382.46
3374.15
1435.83
f1 (ppm)

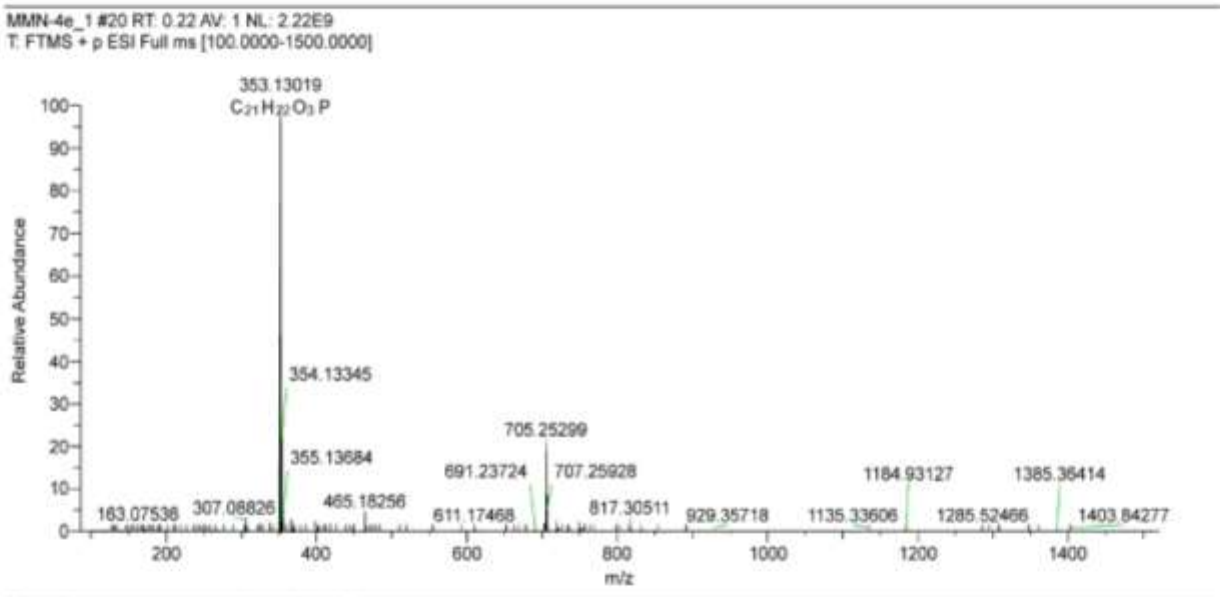

Rank	Peak Mass	Display Formula	Delta [ppm]	Theo. mass	Pattern Cov. [%]	# Matched Iso.	Combined Score	MS Cov. [%]	MSMS Matched...
1	353.13019	$C_{21}H_{22}O_3P$	0.23	353.13011	99.39	5	96.74	99.91	(Collection)
2	353.13019	[illegible]	-0.32	353.13030	85.65	2	76.98	80.8	(Collection)
3	353.13019	[illegible]	-0.33	353.13030	85.87	3	76.91	80.8	(Collection)

S14: Ethyl 3-(2-oxido-2-phenyl-1*H*-isophosphinolin-3-yl)-2-((2-oxido-2-phenyl-1*H*-isophosphinolin-3-yl)methyl)acrylate **(3jc)**

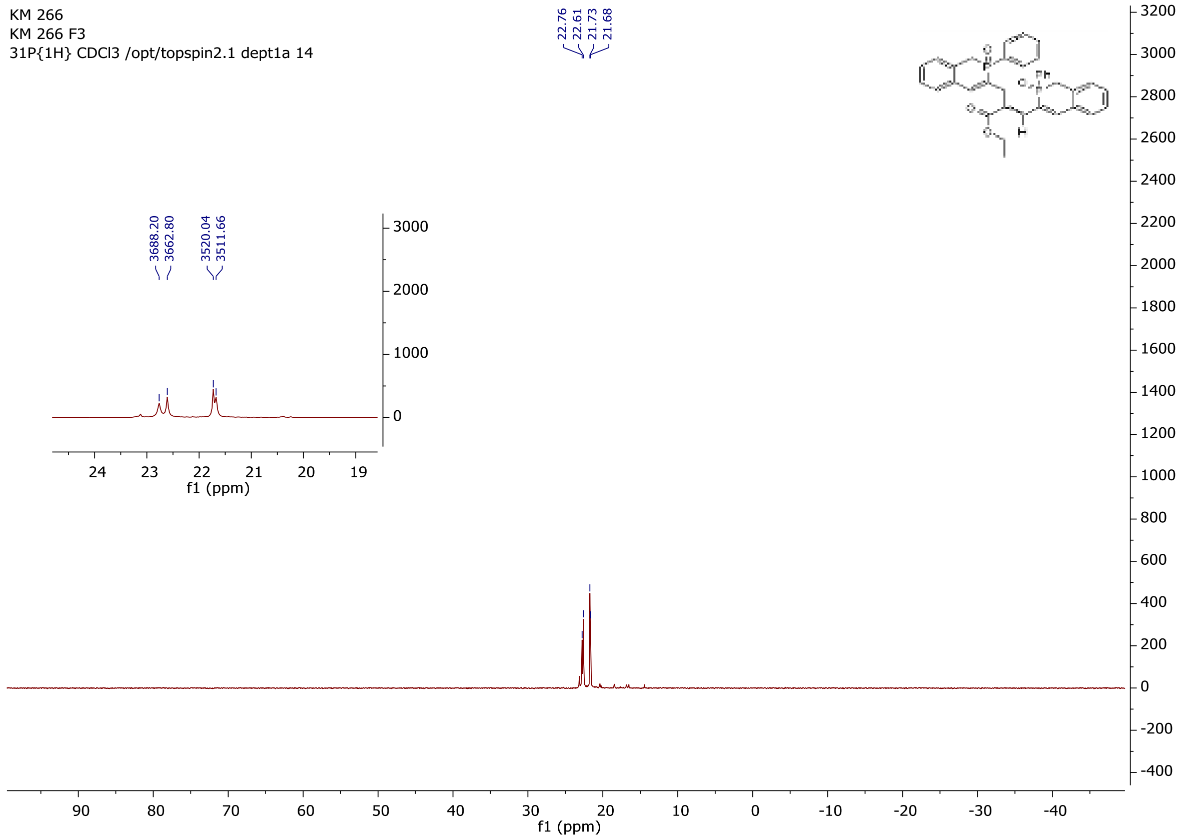

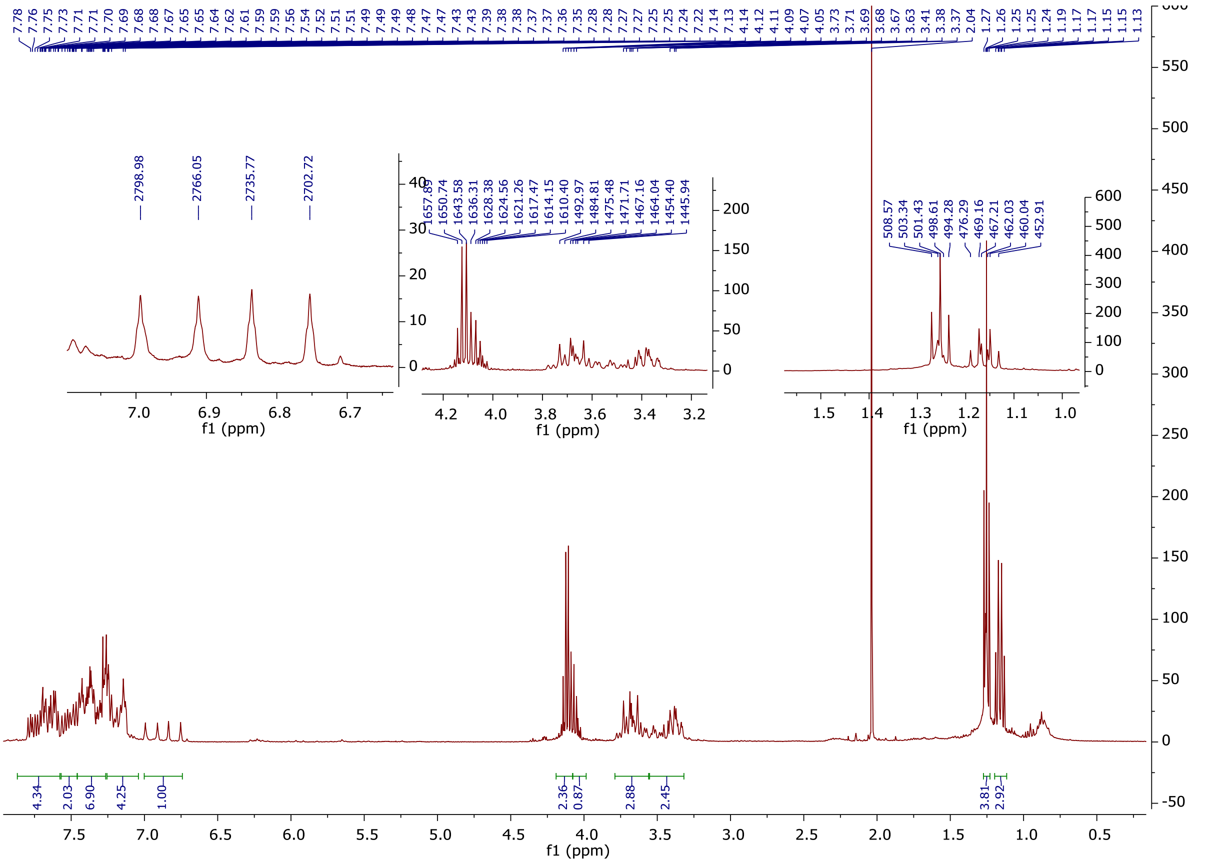
7.78 7.76 7.75 7.73 7.71 7.71 7.70 7.69 7.68 7.68 7.67 7.65 7.65 7.64 7.62 7.61 7.59 7.59 7.56 7.54 7.52 7.51 7.51 7.49 7.49 7.48 7.47 7.47 7.43 7.43 7.39 7.38 7.38 7.37 7.37 7.36 7.35 7.28 7.28 7.27 7.27 7.25 7.25 7.24 7.22 7.14 7.13 4.14 4.12 4.11 4.09 4.07 4.05 3.73 3.71 3.69 3.68 3.67 3.63 3.41 3.38 3.37 2.04 1.27 1.26 1.25 1.25 1.24 1.19 1.17 1.17 1.15 1.15 1.13
2798.98 2766.05 2735.77 2702.72
1657.89 1650.74 1643.58 1636.31 1628.38 1624.56 1621.26 1617.47 1614.15 1610.40 1492.97 1484.81 1475.48 1471.71 1467.16 1464.04 1454.40 1445.94
508.57 503.34 501.43 498.61 494.28 476.29 469.16 467.21 462.03 460.04 452.91
4.34 2.03 6.90 4.25 1.00 2.36 0.87 2.88 2.45 3.81 2.92
f1 (ppm)

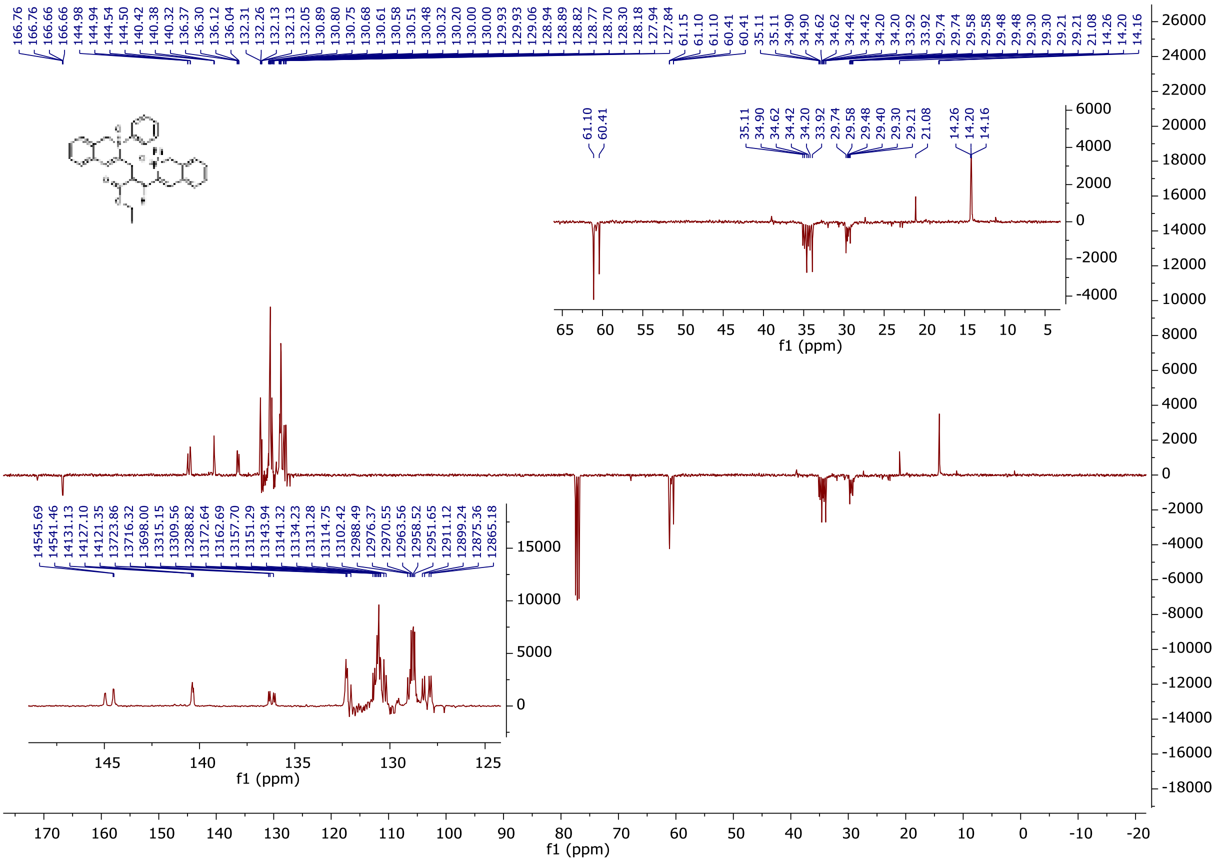
166.76 166.76 166.66 166.66 144.98 144.94 144.54 144.50 140.42 140.38 140.32 136.37 136.30 136.12 136.04 132.31 132.26 132.13 132.05 130.89 130.80 130.75 130.68 130.61 130.58 130.51 130.48 130.32 130.20 130.00 130.00 129.93 129.93 129.06 128.94 128.89 128.82 128.77 128.70 128.30 128.18 127.94 127.84 61.15 61.10 61.10 60.41 35.11 35.11 34.90 34.62 34.62 34.42 34.42 34.20 34.20 33.92 33.92 29.74 29.74 29.58 29.58 29.48 29.48 29.40 29.30 29.30 29.21 21.08 14.26 14.20 14.16
61.10 60.41
35.11 34.90 34.62 34.42 34.20 33.92 29.74 29.58 29.48 29.40 29.30 29.21 21.08 14.26 14.20 14.16
14545.69 14541.46 14131.13 14127.10 14121.35 13723.86 13716.32 13698.00 13315.15 13309.56 13288.82 13172.64 13162.69 13157.70 13151.29 13143.94 13141.32 13134.23 13131.28 13114.75 13102.42 12988.49 12976.37 12970.55 12963.56 12958.52 12951.65 12911.12 12899.24 12875.36 12865.18
f1 (ppm)

High Resolution Mass Result

Analysis Info

Sample Name **MMN-9 c _ KM-27**

Acquisition Date 3/11/2024 1:28:35 PM

Instrument / Ser# micrOTOF-Q 228888.10300

Acquisition Parameter

Source Type ESI Ion Polarity Positive Scan Begin 50 m/z Scan End 3000 m/z

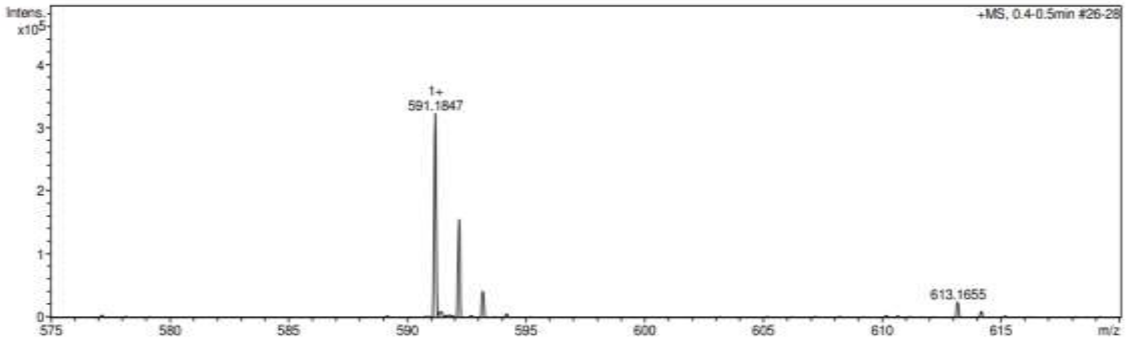

Meas. m/z	#	Ion Formula	Score	m/z	err [mDa]	err [ppm]	mSigma	rdb	e⁻ Conf	N-Rule	Adduct
591.1847	1	C37H29N4P2	65.28	591.1862	1.5	2.6	37.1	26.5	even	ok	M+H
	2	C36H33O4P2	100.00	591.1849	-0.2	-0.3	47.8	21.5	even	ok	M+H
613.1655	1	C36H32NaO4P2	100.00	613.1668	-1.3	-2.2	4.7	21.5	even	ok	M+Na
1181.3600	1	C72H65O8P4	100.00	1181.3624	2.4	2.1	101.3	42.5	even	ok	2M+H
1198.3887	1	C72H68NO8P4	100.00	1198.3890	-0.2	-0.2	505.3	41.5	even	ok	2M+NH4
1203.3425	1	C72H64NaO8P4	100.00	1203.3444	-1.9	-1.6	7.3	42.5	even	ok	2M+Na

S15: (E)-N-(4-methoxybenzyl)-3-(2-oxido-2-phenyl-1H-isophosphinolin-3-yl)acrylamide 3k

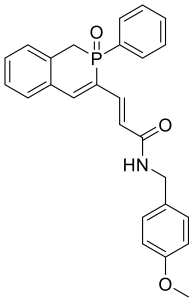

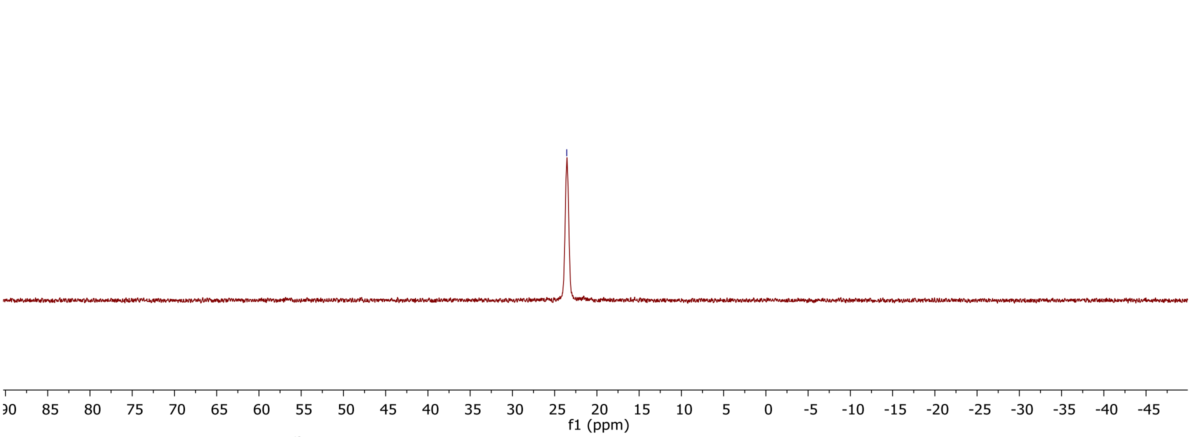

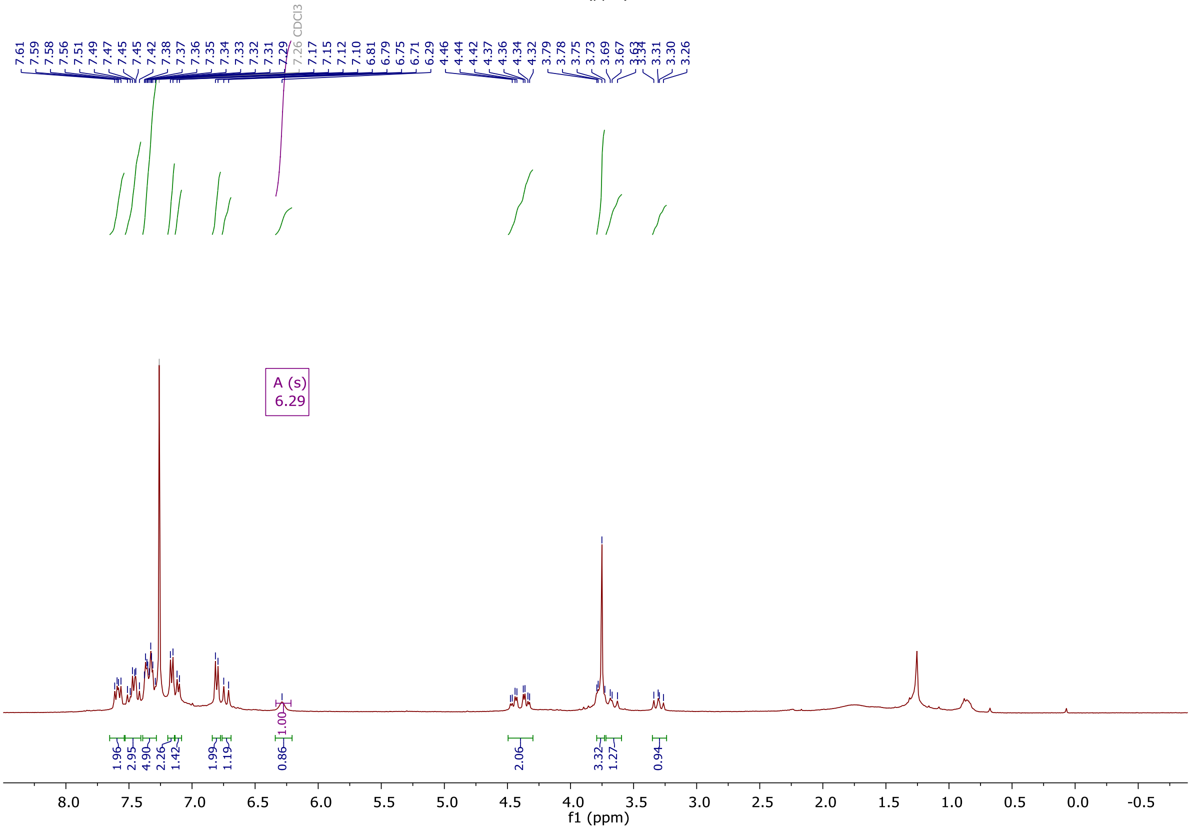

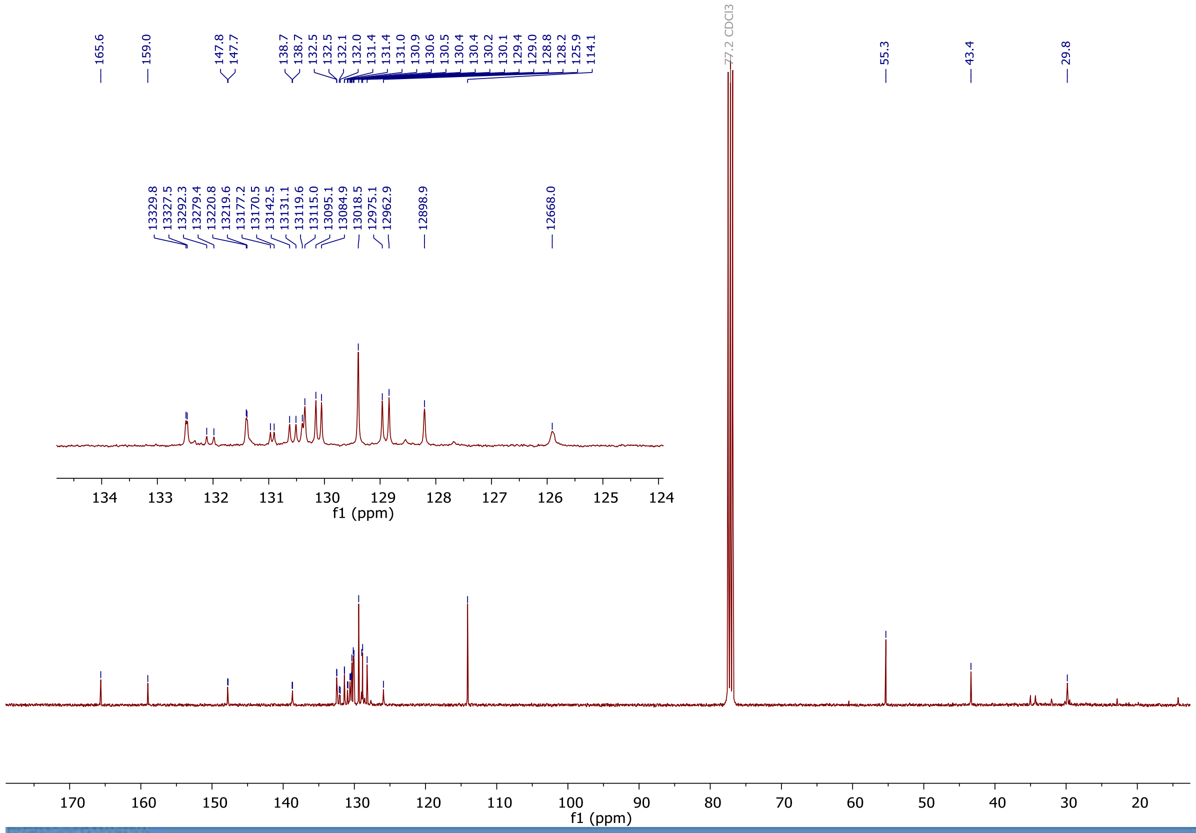
165.6
159.0
147.8
147.7
138.7
138.7
132.5
132.5
132.1
132.0
131.4
131.4
131.0
130.9
130.6
130.5
130.4
130.4
130.2
130.1
129.4
129.0
128.8
128.2
125.9
114.1
77.2 CDCl3
55.3
43.4
29.8
13329.8
13327.5
13292.3
13279.4
13220.8
13219.6
13177.2
13170.5
13142.5
13131.1
13119.6
13115.0
13095.1
13084.9
13018.5
12975.1
12962.9
12898.9
12668.0
f1 (ppm)
f1 (ppm)
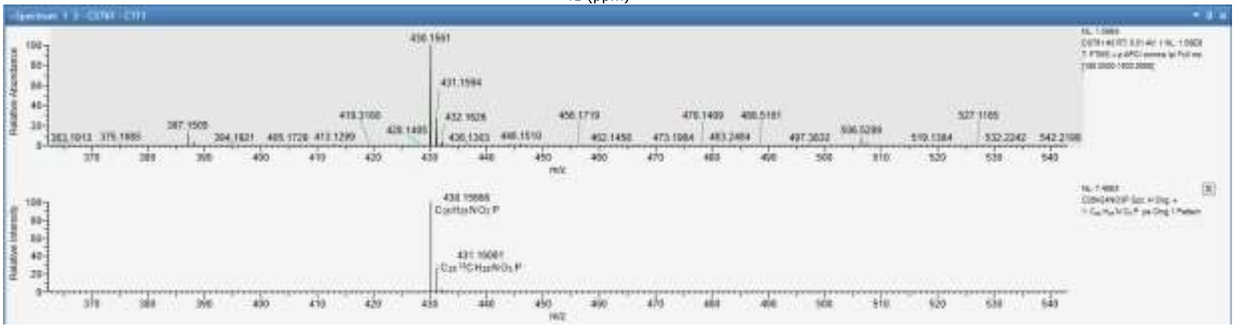
430.1591
431.1594
430.15886
431.15081
m/z

S16: (E)-2-Phenyl-3-(2-(phenylsulfonyl)vinyl)-1H-isophosphinoline 2-oxide **3l**

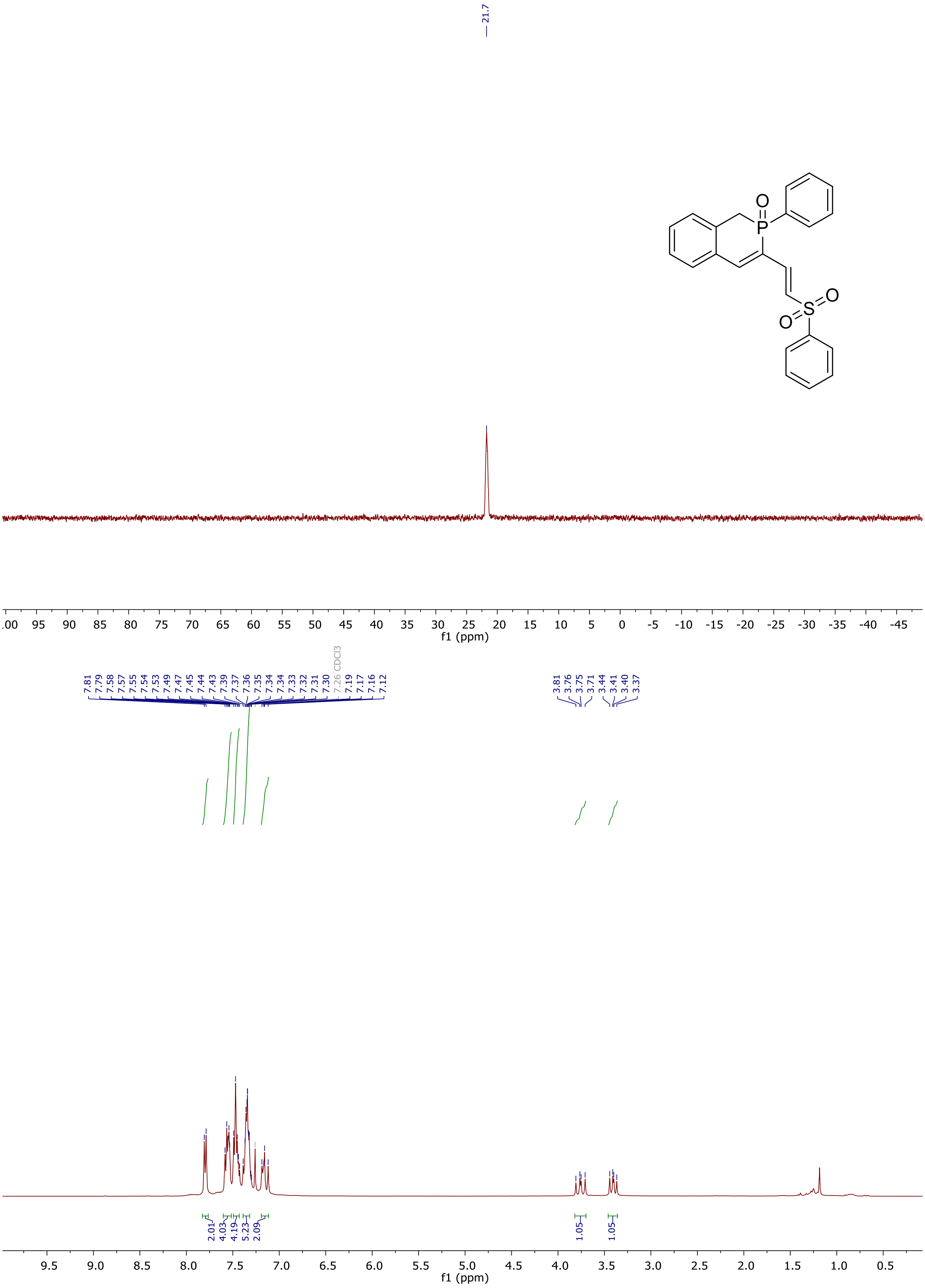

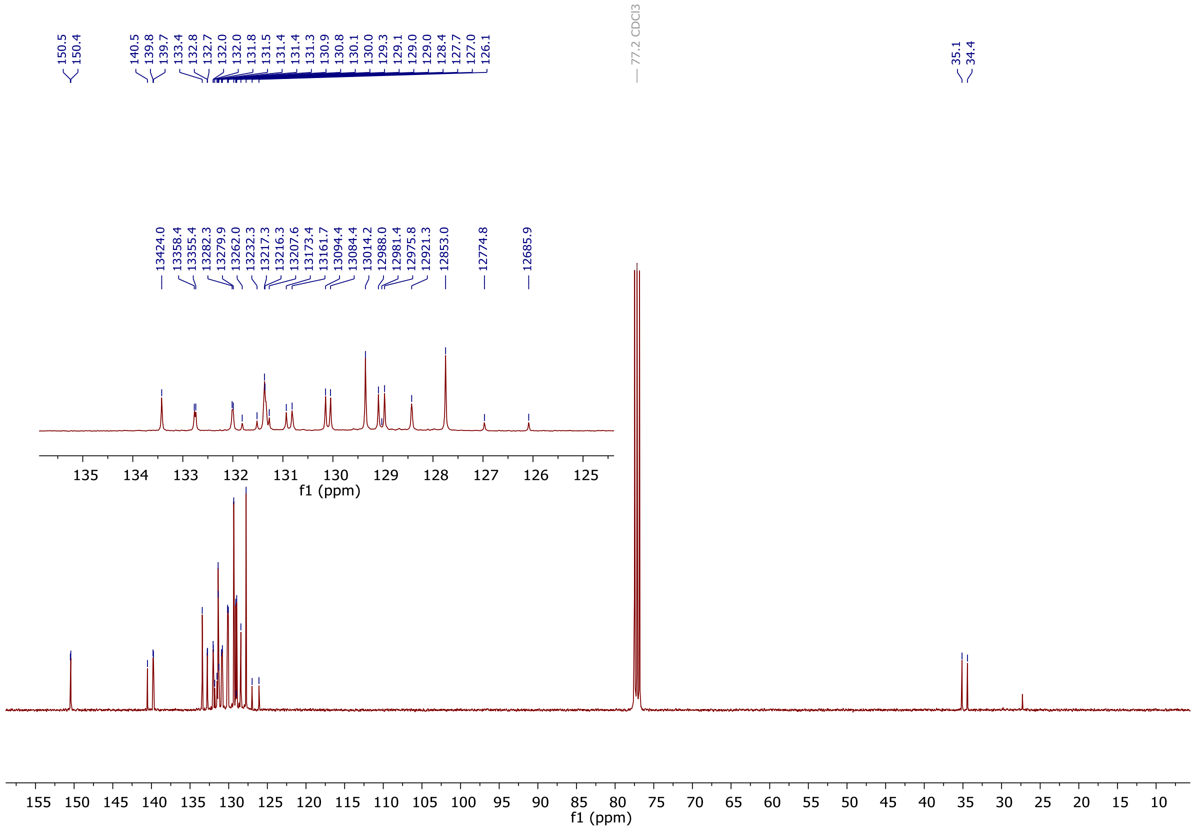
150.5
150.4
140.5
139.8
139.7
133.4
132.8
132.7
132.0
132.0
131.8
131.5
131.4
131.4
131.3
130.9
130.8
130.1
130.0
129.3
129.1
129.0
129.0
128.4
127.7
127.0
126.1
77.2 CDCl3
35.1
34.4
13424.0
13358.4
13355.4
13282.3
13279.9
13262.0
13232.3
13217.3
13216.3
13207.6
13173.4
13161.7
13094.4
13084.4
13014.2
12988.0
12981.4
12975.8
12921.3
12853.0
12774.8
12685.9
f1 (ppm)
f1 (ppm)

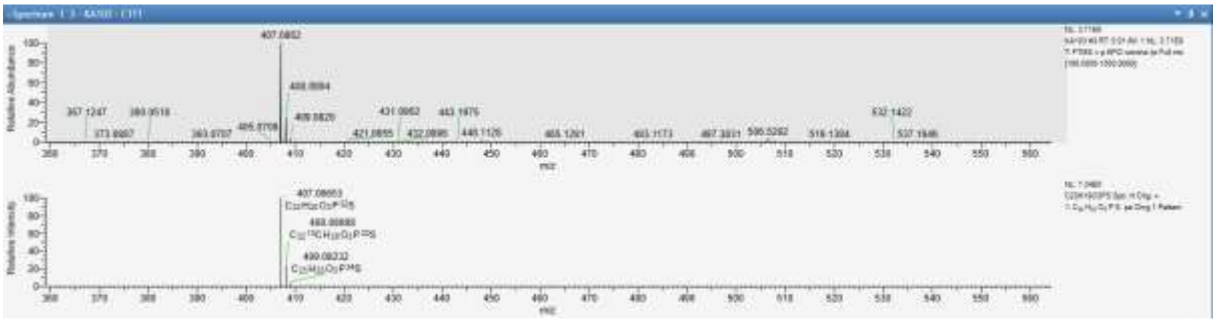
407.0852
408.0884
409.0820
407.08653
408.08988
409.08232

S17: (E)-2-(2-(2-oxido-2-phenyl-1H-isophosphinolin-3-yl)vinyl)isoindoline-1,3-dione **3m**

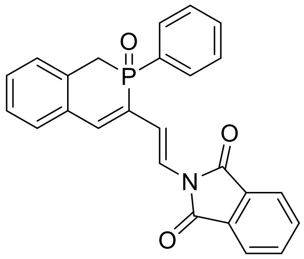

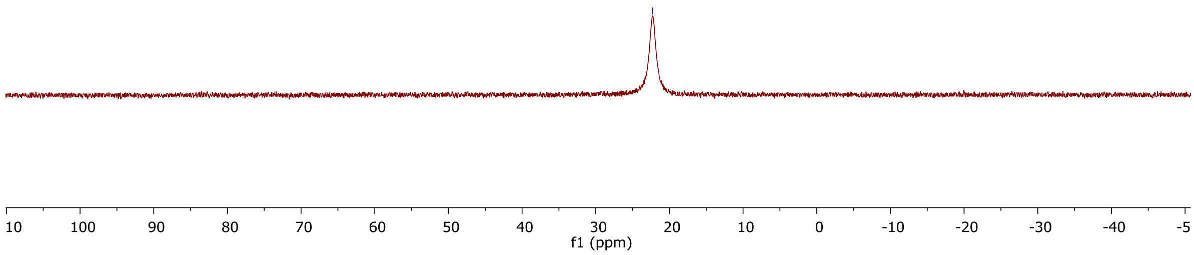

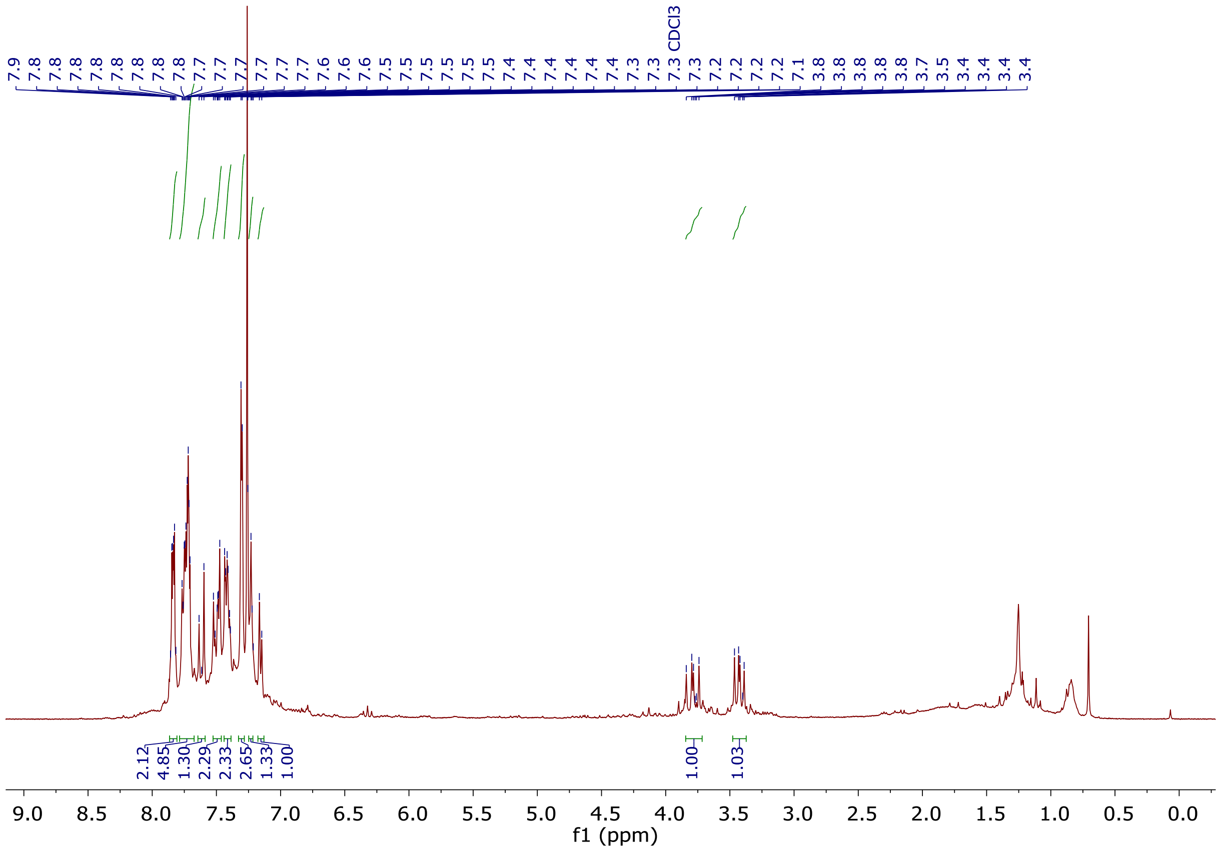

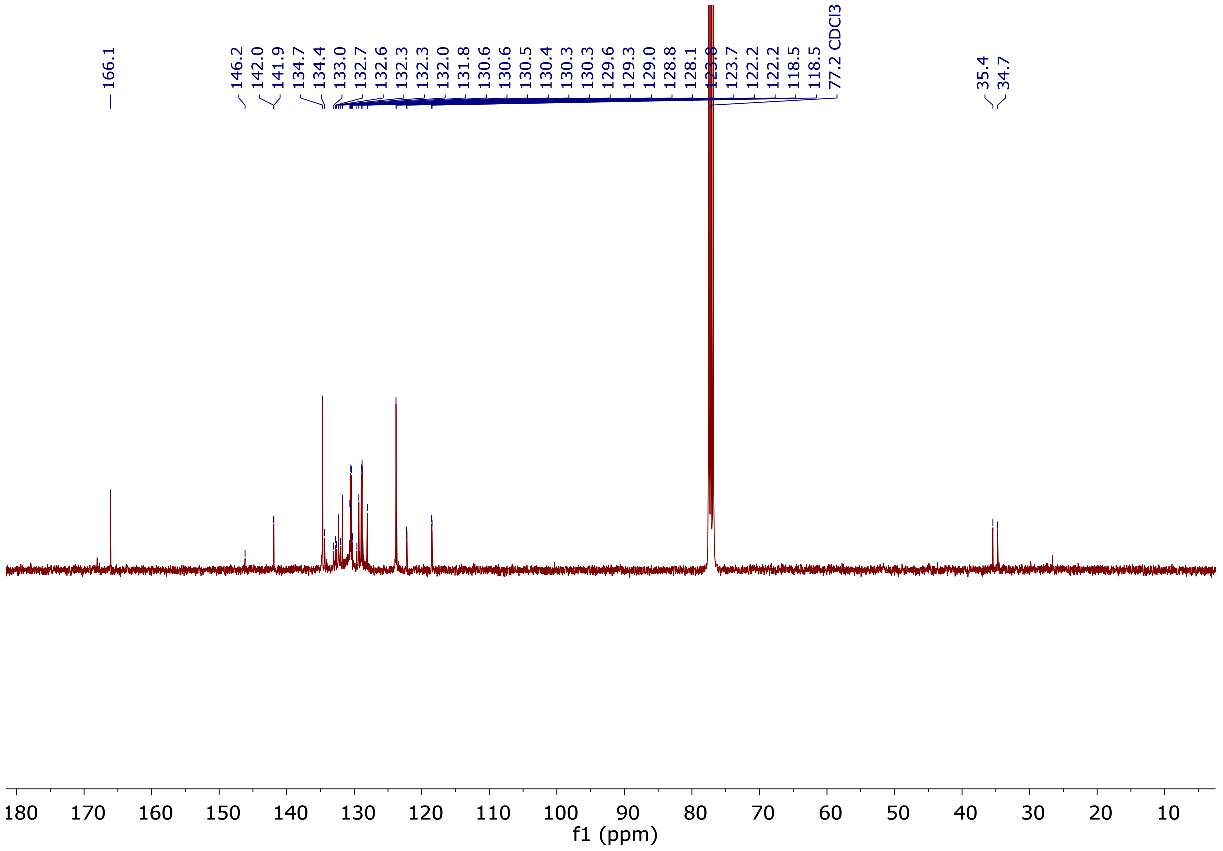
166.1
146.2
142.0
141.9
134.7
134.4
133.0
132.7
132.6
132.3
132.3
132.0
131.8
130.6
130.6
130.5
130.4
130.3
130.3
129.6
129.3
129.0
128.8
128.1
123.8
123.7
122.2
122.2
118.5
118.5
77.2 CDCl3
35.4
34.7
180
170
160
150
140
130
120
110
100
90
80
70
60
50
40
30
20
10
f1 (ppm)

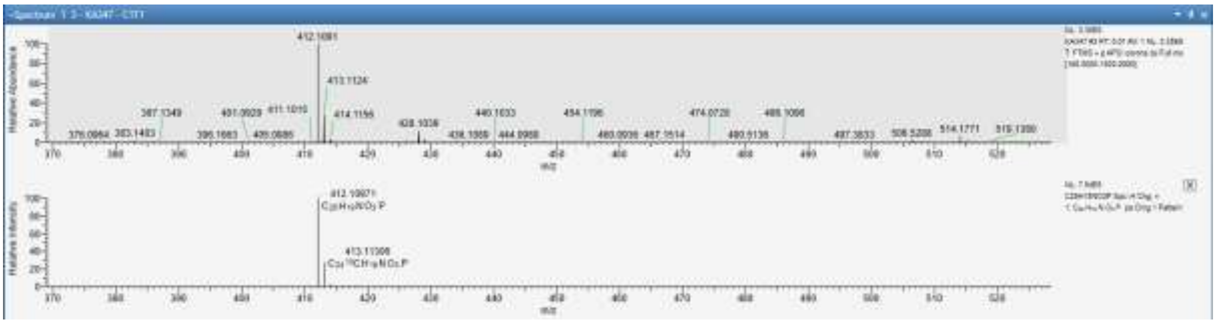

Crystallographic data of sulfonated product **3l**
